# Alternating behavior in furan-acetylene macrocycles reveals the size-dependency of Hückel’s rule in neutral molecules

**DOI:** 10.1038/s42004-023-00902-9

**Published:** 2023-05-27

**Authors:** Yuval Rahav, Shinaj K. Rajagopal, Or Dishi, Benny Bogoslavsky, Ori Gidron

**Affiliations:** Institute of Chemistry, The Center for Nanoscience and Nanotechnology, Casali Center for Applied Chemistry, The Hebrew University of Jerusalem, Edmond J. Safra Campus, Jerusalem, 9190401 Israel

**Keywords:** Organic chemistry, Physical chemistry

## Abstract

Aromaticity can be assigned by Hückel’s rule, which predicts that planar rings with delocalized (4*n* + 2) π-electrons are aromatic, whereas those with 4*n* π-electrons are antiaromatic. However, for neutral rings, the maximal value of “*n”* to which Hückel’s rule applies remains unknown. Large macrocycles exhibiting global ring current can serve as models for addressing this question, but the global ring current are often overshadowed in these molecules by the local ring current of the constituent units. Here, we present a series of furan-acetylene macrocycles, ranging from the pentamer to octamer, whose neutral states display alternating contributions from global aromatic and antiaromatic ring currents. We find that the odd-membered macrocycles display global aromatic characteristics, whereas the even-membered macrocycles display contributions from globally antiaromatic ring current. These factors are expressed electronically (oxidation potentials), optically (emission spectra), and magnetically (chemical shifts), and DFT calculations predict global ring current alternations up to 54 π-electrons.

## Abstract (web summary)

Hückel’s rule predicts that planar rings with delocalized (4n + 2) π-electrons are aromatic, whereas those with 4n π-electrons are antiaromatic, but for neutral rings the maximum value of n for which Hückel’s rule applies remains unclear. Here, the size-dependency of Hückel’s rule is studied in a series of furan-acetylene macrocycles whose neutral states display alternating contributions from global aromatic and antiaromatic ring currents.

## Introduction

Planar cyclic molecules with π-conjugation display aromaticity or antiaromaticity depending on their number of π-electrons, 4*n* + 2 or 4*n* respectively, according to Hückel’s rule^1^. While aromaticity is expressed in energetic, geometric, and optical terms^2,3^, it is most commonly quantified in magnetic terms, with either diatropic or paratropic ring currents for aromatic or antiaromatic compounds, respectively^4,5^. It is estimated that, for neutral annulenes, Hückel’s rule is effective for ≤30 π-electrons, but theoretically it can also be valid for larger annulenes^6,7^. However, distortion from planarity with increased degrees of freedom, as well as increasing numbers of stereoisomers, prevents the experimental validation of this assumption. π-Conjugated macrocycles, which are composed of smaller aromatic rings in a cyclic arrangement, are shape-persistent, often more stable than annulenes, and are expected to show similar trends and thus produce aromatic or antiaromatic global ring currents^8–11^.

**Fig. 1:**
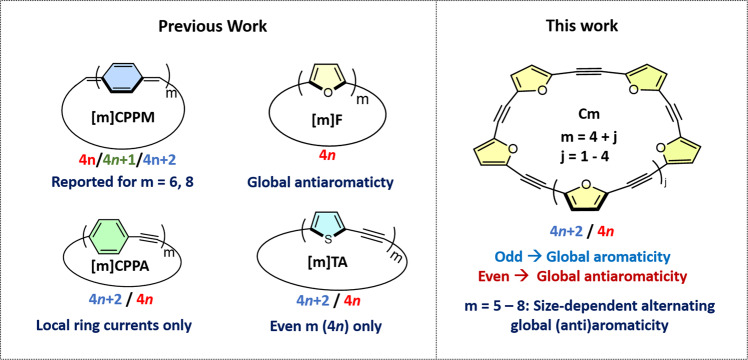
Macrocycles with alternating and non-alternating global aromaticity. The colored text by each structure refers to the number of π-electrons (*n*) that may be involved in global ring current for macrocycles composed of an odd (blue) or even (red) numbers of member rings (*m*).

Methine-spaced macrocycles, such as porphyrins, have a distinct quinoid character, which is expressed in a double bond between the methine and the α-carbon^12^. Consequently, they show aromatic or antiaromatic global ring currents, depending on their size^13,14^. For example, Wu’s group demonstrated that for [m]-cycloparaphenyl-methines (**[*m*]CPPM**, where *m* is the number of linked rings, in this case, phenyl groups, comprising the macrocycle; Fig. 1), the hexamer **[6]CPPM** displays global antiaromaticity at low temperatures, whereas **[8]CPPM** is distorted from planarity and ‘escapes’ antiaromaticity^15,16^. Anand’s group demonstrated different absorption spectra for furan-containing porphyrinoid macrocycles containing 30 π- and 40 π-electrons^17^. Anderson’s group demonstrated that acetylene-spaced porphyrin nanorings display global aromaticity in different oxidation states, even for 162 π-electrons^18^, including in their excited state^19^. However, these global ring currents were not detected in their neutral ground state^20^.

In contrast, neutral non-quinoid macrocycles, connected by ethylene, acetylene, or any other linker with an even number of carbon atoms, are not commonly reported to show global ring current^21^. For example, **[*m*]CPPA** series members have 6 π-electrons per repeat unit that can contribute to the global ring current. Therefore, global current in even-membered **[*m*]CPPA** macrocycles consist of 4*n* π-electrons, whereas odd-membered **[m]CPPA** macrocycles have 4*n* + 2 π-electrons. As a result, the series members should alternate between global aromatic and antiaromatic ring currents. However, the ^1^H-NMR chemical shift does not display the expected alternating trend for odd and even *m*^22^. One explanation is that the local aromaticity of the benzene ring overshadows any global effects. Therefore, reducing local aromaticity by replacing the benzene rings with less-aromatic thiophene or furan units is expected to increase the expression of global ring current. However, thiophene-acetylene macrocycles (**[*m*]-TA**, Fig. 1) have been reported only for even-membered macrocycles, and therefore the expected aromatic/antiaromatic alternation of global ring current cannot be explored experimentally^23^.

We have previously introduced oligofurans, which display weaker aromaticity and therefore stronger quinoid character compared with their thiophene analogs^24,25^. The lower aromaticity of furan is expressed in greater chemical reactivity (for example, as a diene in Diels-Alder cycloaddition)^26,27^, shorter interring distances^24^, as well as in magnetic ring currents. The *exo* angle of furan is predicted to favor macrocycles with significantly smaller sizes than can be obtained from thiophenes^28^. Macrocyclic furans (**[*m*]F**, Fig. 1) of smaller sizes are planar and display a contribution from globally antiaromatic ring current in their neutral state and from globally aromatic ring current in their dicationic state^29–31^. Larger macrocyclic furans are distorted out of planarity, thereby avoiding global antiaromaticity^32^. However, since each furan ring contributes 4 π-electrons to the global ring current of macrocyclic furans, they are expected to be globally antiaromatic regardless of the number of rings. Märkl et al., explored oxaporphyrin with 4*n* and 4*n* + 2 π-electrons, however the presence of multiple isomers prevented drawing clear conclusions regarding the contribution of global aromatic ring currents^33,34^. Therefore, the size-dependency of Hückel’s rule in neutral macrocycles remains an open question.

Here, we introduce a series of furan-acetylene macrocycles, **C*m*** with *m* = 5–8 furan rings (Fig. 1). Unlike previous macrocyclic furans with 4*n* π-electron systems, the 6 π-electrons per repeat unit in the **C*m*** series is expected to display alternating global aromatic and antiaromatic ring currents, for odd and even *m*, respectively. Indeed, our experimental results show that **C*m*** series members in their neutral state exhibit alternating properties for odd- and even-membered rings. All the magnetic (NMR), photophysical (absorption and fluorescence spectra), and electrochemical properties of these furan-acetylenes indicate a contribution from global antiaromaticity in even-membered macrocycles, whereas odd-membered macrocycles are globally aromatic. Nucleus independent chemical shifts (NICS) calculations support this trend, predicting that the contribution of the global ring current is significant up to 54 π-electrons in neutral **C*m***. Overall, this work highlights the prospect of furan serving as a probe for global aromatic effects in macrocycles.

## Results and discussion

### Synthesis and structure

To synthesize the **C*m*** series, comprising both odd- and even-membered macrocycles, we adopted the monomer rather than the oligomer approach. For **[*m*]TA** (Fig. 1), the monomer approach is unlikely to produce small macrocycles, since the angle between the α substituents is 150°, which is ideal for 12 membered macrocycles. In contrast, the angle between the α substituents of furan is 125°, which should be ideal for 6–7 membered macrocycles^28^. Therefore, to obtain a sequential series of small macrocycles with 6 π-electrons per unit, macrocyclization of 2-ethynyl-furan units was the method of choice.

**Fig. 2:**
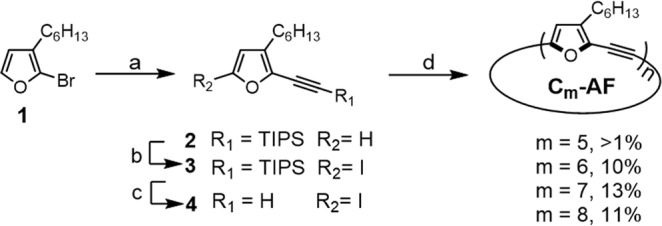
Synthesis of C*m* (*m* = 5–8 rings). Reagents and conditions: (**a**) TIPS-acetylene, (Pd(PPh_3_)_2_Cl_2_), Et_3_N, reflux, 2 days; (**b**) I_2_ (3 eq), lithium diisopropylamine (1 M), THF, -78 °C; (**c**) tetrabutylammonium fluoride (1 M), THF, 3 h; (**d**) Pd(PPh_3_)_4_ (10% mol), CuI (3% mol), toluene:diisopropylethylamine (1:1), 60 °C, 4 days. Et ethyl; Ph phenyl; TIPS triisopropylsilyl; THF tetrahydrofuran.

The synthesis of **C*m*** is depicted in Fig. 2. 2-Bromo-3-hexylfuran (**1**)^35^, was coupled to triisopropylsilyl-acetylene by Sonogashira coupling using triethylamine as a solvent in the presence of bis(triphenylphosphine)palladium(II) dichloride or copper iodide to obtain **2** with a 78% yield. The hexyl-groups were found to be important for the solubility of both the intermediates during the macrocyclization step and the final macrocycles. Lithiation of **2** with lithium-diisopropylamine followed by dropwise addition of a solution of I_2_ in tetrahydrofuran afforded **3**, and the triisopropylsilyl group was subsequently removed with tetra-*n*-butylammonium fluoride to afford **4** with an overall yield of 60%.

**Fig. 3:**
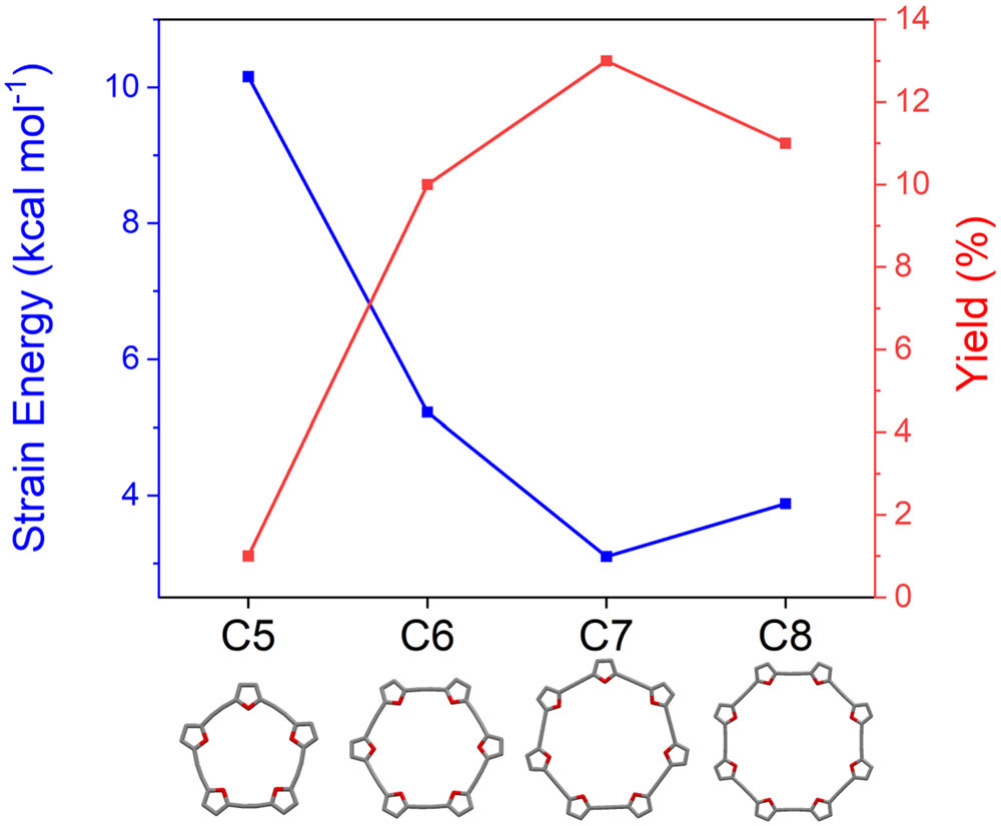
Optimized structures and calculated strain energies of the C*m* series (*m* = 5–8 rings). All structures and energies were calculated at the DFT/B3LYP/6-31 G(d) level.

For the macrocyclization of **4** using Sonogashira coupling, we attempted modification of several parameters, including solvent, base, temperature, and reactant concentration (see Supplementary Table 1). We found that heating a toluene/diisopropylethylamine solution of **4** (22 mM) to 60 °C with palladium-tetrakis(triphenylphosphine) (10% mol) and copper (I) iodide (3% mol) for four days produced **C5–C8** with an overall yield of 35%. The macrocycles were then separated using size exclusion chromatography. The size distribution is in-line with the calculated strain (Fig. 3), where **C7** displays the lowest strain energy (3.1 kcal mol^−1^) and highest yield (13%) and **C5** displays the highest strain energy (10.1 kcal mol^−1^) and lowest yield (<1%).

**Fig. 4:**
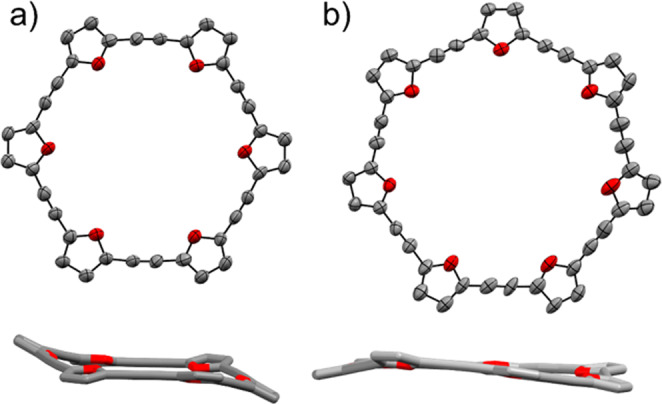
X-ray structures of an even- and odd-membered example compound from the C*m* series. X-ray structures of (**a**) **C6** and (**b**) **C7**. Hydrogen atoms and alkyl groups are omitted for clarity.

Crystals of **C6** and **C7** were grown by slow evaporation from hexane and chloroform solutions. The solid-state structures are displayed in Fig. 4. The average dihedral angle (see Supplementary X-Ray Crystallography S6.1) for **C6** is 26°, and in the solid state it adopts a chair conformation, similar to cyclohexane. **C7** is significantly more planar, with an average dihedral angle of 12°, which also corresponds to the abovementioned lower strain energy. The calculated structures for **C*m*** are nearly planar for all sizes, with an average dihedral angle of 1° or less. We note that, as the barrier to rotation around the triple bond is low, the average conformation in solution is also expected to be planar or nearly-planar, as indicated by the calculated structures. The C ≡ C bond lengths do not display any significant changes (computationally or experimentally) between the different members of the **C*m*** series, and this also applies to other bond distances in the π-conjugated backbone. This is consistent with previously reported examples, in which alternating global aromaticity in large (>20 π-electrons) π-conjugated macrocycles was not expressed in the bond distances^20^.

### Redox properties

**Fig. 5:**
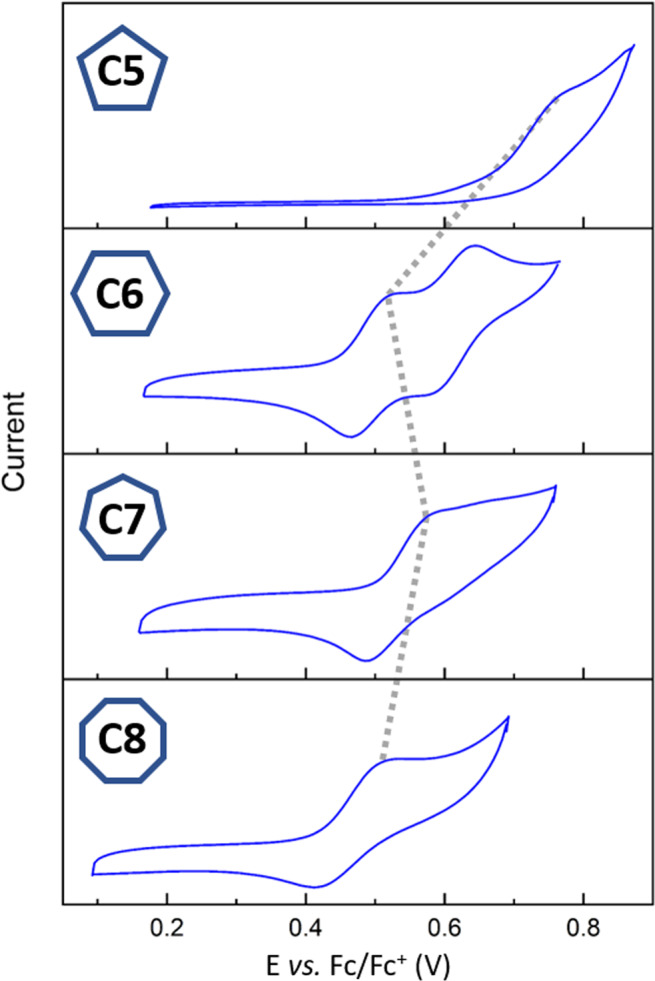
Cyclic voltammograms of the C*m* series (*m* = 5–8 rings). All measurements were calibrated *vs*. the Fc/Fc^+^ redox couple in dichloromethane solvent using 0.1 M (nBu)_4_NClO_4_ (TBAPC) as an electrolyte (scan rate 100 mV s^−1^).

Figure 5 presents the cyclic voltammograms of **C*m*** in dichloromethane. The oxidation potential (anodic peak, E^pa^) of **C5** is 0.25 V higher compared with **C6** (E^pa^ = 0.77 V and 0.52 V for **C5** and **C6**, respectively). **C6** displays two distinct reversible oxidation peaks. **C7** also exhibits a higher oxidation state compared with **C6**, although the difference is only 0.07 V (E^pa^ = 0.59 V) and the oxidation potential for **C8** is again lower than **C7** (E^pa^ = 0.52 V). **C6** stands out as the only member of the **C*m*** series that exhibits two distinct reversible oxidation peaks, and we have previously observed similar behavior from the antiaromatic 8-membered furan macrocycle^30^. Therefore, **C6** is the only member that exhibits a significant contribution from global antiaromatic ring current. The antiaromatic character of **C6** is quite pronounced, whereas the cyclic voltammogram of **C8** is not affected significantly by the global antiaromaticity.

As a result of their global aromatic character, odd-membered **C*m*** are expected to exhibit higher oxidation potentials compared with their even-membered analogs, since oxidation to the cation is expected to result in the loss of aromaticity, and further oxidation to the dication is expected to result in the emergence of an antiaromatic contribution. Thus, the alternating trend of the oxidation potentials is consistent with the expected trend of global aromaticity/antiaromaticity. We note that the redox potential of charged macrocycles was previously suggested as a tool to probe global antiaromaticity^36^.

The calculated (M06/6-311 G(d)) energy of the highest occupied molecular orbital (HOMO) of **C5** is c.a. 0.3 eV lower than that of **C6** (−5.41 eV *vs*. −5.12 eV, respectively), which corresponds to the abovementioned trend in the experimentally observed oxidation potentials. The HOMO of **C7** (−5.27 eV) is even lower compared with **C6** and the HOMO of **C8** (−5.11 eV) is higher compared with **C7**. Overall, the alternating behavior of the HOMO energy levels with size matches the trends observed experimentally in the electrochemical oxidation. The difference between the oxidation potentials of **C6** and **C8** can be explained by the difference between their calculated HOMOs, which is 0.3 eV higher for **C8**. It is interesting to note that macrocycles with formal antiaromatic systems were previously applied as active materials in batteries since they display stable redox states^37^.

To study further the stability of the oxidized species, we attempted to chemically oxidize **C*m*** with an excess of nitrosonium hexafluoroantimonate (NOSbF_6_), which was found to be an effective oxidant of macrocyclic furans^28^. Oxidation of **C6** resulted in two sets of spectra, depending on the amount of oxidant added. The first set of spectra, showing a broad absorption band at 1744 nm, and can be attributed to the cation radical, whereas the second set with bands at 1130 nm and 550 nm, can be attributed to the dication (see Supplementary Fig. 46). For **C8**, stepwise oxidation resulted in only one specie, which can be attributed to the cation radical. In contrast, attempts to oxidize **C5** and **C7** resulted in quick decomposition, even under an inert atmosphere (N_2_-filled glovebox), with no apparent absorption bands that can be attributed to cationic or dicationic species. The greater stabilization upon oxidization of **C6** and **C8** compared with **C5** and **C7** is further evidence of the aromatic character of dicationic **C6** and cationic **C8**.

### Photophysical properties

**Fig. 6:**
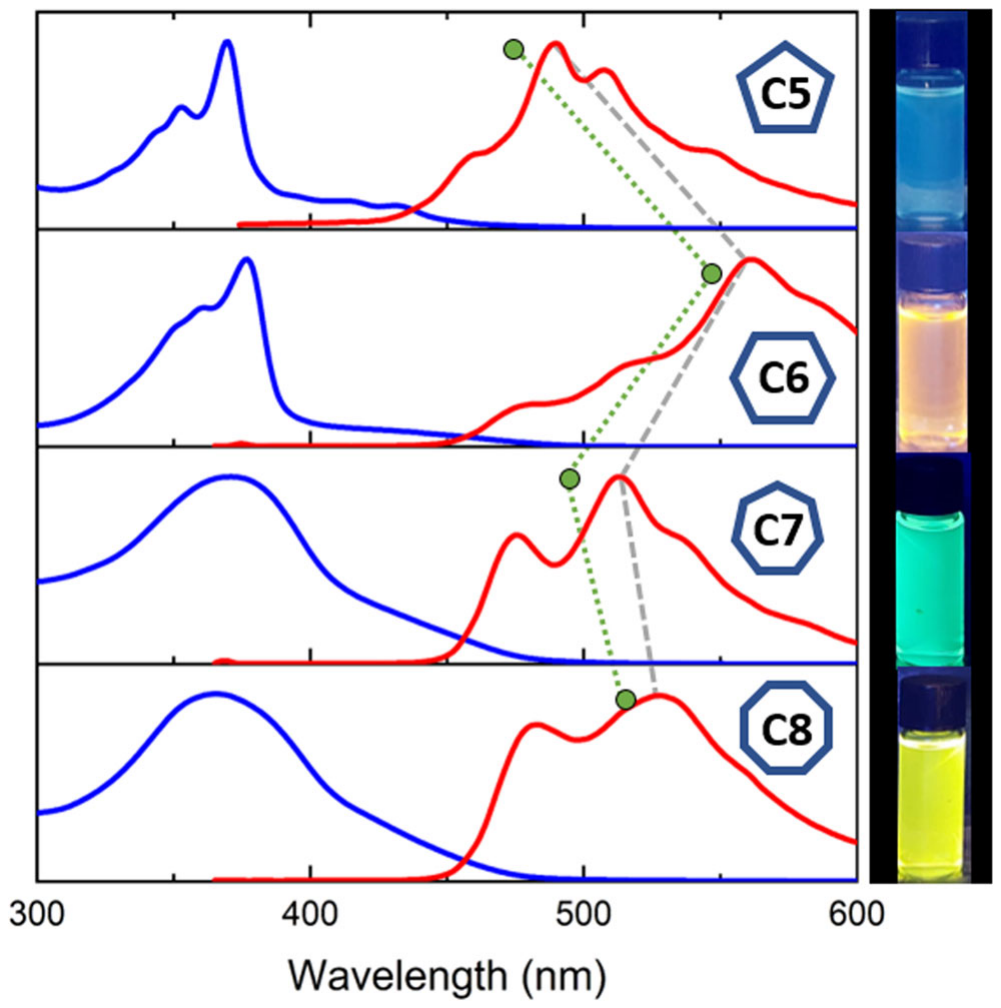
Photophysical properties of the C*m* series (*m* = 5–8 rings). Normalized absorption (blue) and emission (red) spectra of the **C*m*** series in chloroform. Right: Emission by chloroform solutions of **C*m*** irradiated at 365 nm. The gray trace follows the experimental emission maxima. The green trace represents the S1 → S0 transition calculated at the TD-DFT/CAM-B3LYP/6-311 G(d) level of theory, showing the same alternating trend.

The absorption and emission spectra of **C*m*** are depicted in Fig. 6. **C5** and **C6** display relatively sharp absorption bands, with a weak S_0_→S_1_ transition. For **C5**, the vibronic shoulders for the S_0_→S_1_ transition can be observed at 415 nm and 431 nm. We note that, although **C5** is more rigid than **C6**, the relative intensity of the S_0_→S_1_ transition is stronger (by 15% for **C5** and 8% for **C6**) compared with the S_0_→S_*n*_ transition (*n* > 1). This indicates the involvement of additional factors in the oscillator strength. **C7** and **C8** display broader absorption spectra, corresponding to their more flexible structures. In chloroform, the absorption spectra of **C6** and **C7** exhibit concentration dependence above 10^−5 ^M (see Supplementary Figs. 39a, 41). We, therefore, measured the absorption and emission spectra at lower concentrations as well as in toluene, where no concentration dependence was observed (see Supplementary Figs. 38–40, 42b).

The emission spectra for **C5** and **C6** differ markedly from each other: whereas the emission maxima (λ_em_) for **C5** is 490 nm, there is a significant bathochromic shift of 72 nm (0.32 eV) for **C6**. This also manifests in a clear difference in color upon irradiation in black light (blue for **C5** and orange for **C6**, Fig. 6). **C7** again displays a hypochromic shift compared with **C6**, with emission maxima of 513 nm, and the emission maxima for **C8** is again slightly bathochromic (λ_em_ = 533 nm). We note that, although the differences are large, this should be taken with caution, as increasing macrocycle size also results in increased flexibility, which in turn affects the emission properties. The fluorescence quantum efficiencies are small (5–6%) for the rigid **C5** and **C6**, and increase significantly for **C7** and **C8** (35 and 37%, respectively). In addition, the emission maxima seem to occur in different vibronic states. Nevertheless, time-dependent density functional theory (TD-DFT) calculations display the same alternating trend as observed experimentally, with S_1_→S_0_ transitions at longer wavelengths for even-membered macrocycles (478 nm for **C5** and 544 nm for **C6**, calculated at the CAM-B3LYP/6-31 G(d) level). The green trace in Fig. 6 represents the calculated emission maxima for **C*m***, showing the same alternating trend as the experimental emission maxima (gray trace). Overall, the emission spectra show alternating behavior, with even-membered rings bathochromically-shifted compared with the odd-membered rings.

**Fig. 7:**
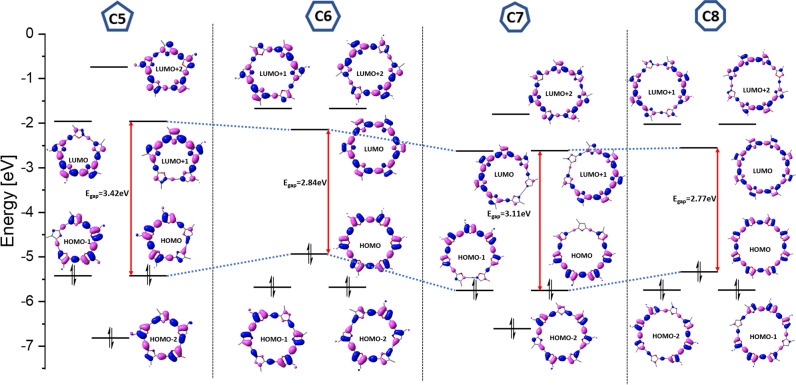
Calculated frontier molecular orbital diagrams of the C*m* (*m* = 5–8 rings) series. Frontier molecular orbitals were calculated at the M06/6-311 G(d) level. The value of the HOMO–LUMO gap is indicated beside the red double-headed arrow, with energy trends across the series indicated by the blue dotted lines.

We utilized DFT to calculate the behavior of the **C*m*** series to understand better the alternating behavior of its members. The frontier molecular orbitals for **C*m*** are depicted in Fig. 7. The gap between the highest occupied and lowest unoccupied molecular orbitals (the HOMO–LUMO gap) is smaller for compounds with a globally antiaromatic character compared with their aromatic counterparts, which is consistent with the alternating fluorescence maxima observed above. This was previously rationalized by Rabinovitz’s group for polyaromatic compounds, who reasoned that antiaromaticity is an antibonding property, and therefore the LUMO is stabilized, whereas the HOMO is destabilized^38^.

As can be observed in Fig. 7, the odd-membered macrocycles (**C5** and **C7**) display degenerate HOMO and LUMO levels, and even-membered macrocycles (**C6** and **C8**) displayed degeneracy of the HOMO-1 and LUMO + 1 levels. This is consistent with the trend for expanded porphyrins previously observed by Sessler and Kim^39^, who suggested optical properties as a parameter for aromaticity, and reported that the difference between aromaticity and antiaromaticity is expressed in red-shifted and weaker emission in globally antiaromatic macrocycles compared with globally aromatic macrocycles.

The difference in degeneracy is explained by the localized π-electron character of the structures with antiaromatic contribution compared with the delocalized electron structure of aromatic macrocycles (it was previously reported that, in antiaromatic systems, π-overlap favors localization of the double bonds)^40^. The HOMO–LUMO gap also alternates between odd and even **C*m***, with the odd-membered (aromatic) **C5** and **C7** displaying larger gaps (3.42 and 3.11 eV, respectively) compared with the even-membered (antiaromatic) **C6** and **C8** (2.84 and 2.77 eV, respectively). We note that for even **C*m***, both the HOMO and LUMO levels have the same number of nodal planes, as expected for antiaromatic compounds on the basis of basic symmetry arguments^41^.

### Magnetic ring current

**Fig. 8:**
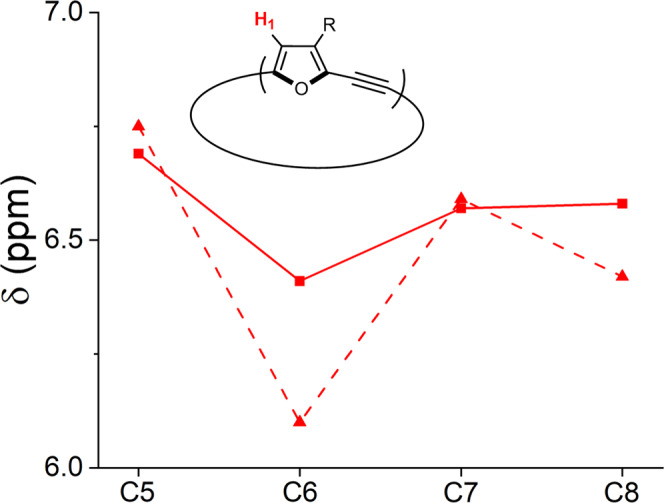
Alternating chemical shifts showing global (anti)aromatic current contribution. Calculated (M06-2X/6-311(d), dashed line) and experimental (solid line) chemical shifts of the *ß*-protons of the furan rings in the **C*m*** series (*m* = 5–8 rings), measured in CDCl_3_.

The ^1^H-NMR spectra of **C*m*** show the dependency of the chemical shifts on macrocycle size. In particular, the protons located at the *β*-position of the furan rings experience an upfield shift of 0.28 ppm for **C6** (6.41 ppm) compared with **C5** (6.69 ppm, Fig. 8 solid line). **C7** shows a downfield shift of 0.16 ppm compared with **C6**, and the chemical shift of **C8** is nearly identical to that of **C7**. The same trend is also observed for the methylene protons of the hexyl chain attached to the furan ring, and to nearly the same extent (see Supplementary Fig. 52). For example, the chemical shift for the methylene protons of **C5** is 2.65 ppm and of **C6** is 2.44 ppm. By comparison, in the acyclic compound **2** (Fig. 2), the chemical shift of the methylene protons is 2.49 ppm, which is almost identical to the value for **C8** (2.50 ppm). The calculated chemical shifts at the M06-2X/6-311 G(d) level show the same trend, with the even-membered **C*m*** members experiencing an upfield shift (Fig. 8, dashed line). This can be rationalized by the diatropic and paratropic global ring currents affecting the external protons in global aromatic and antiaromatic currents, respectively.

**Fig. 9:**
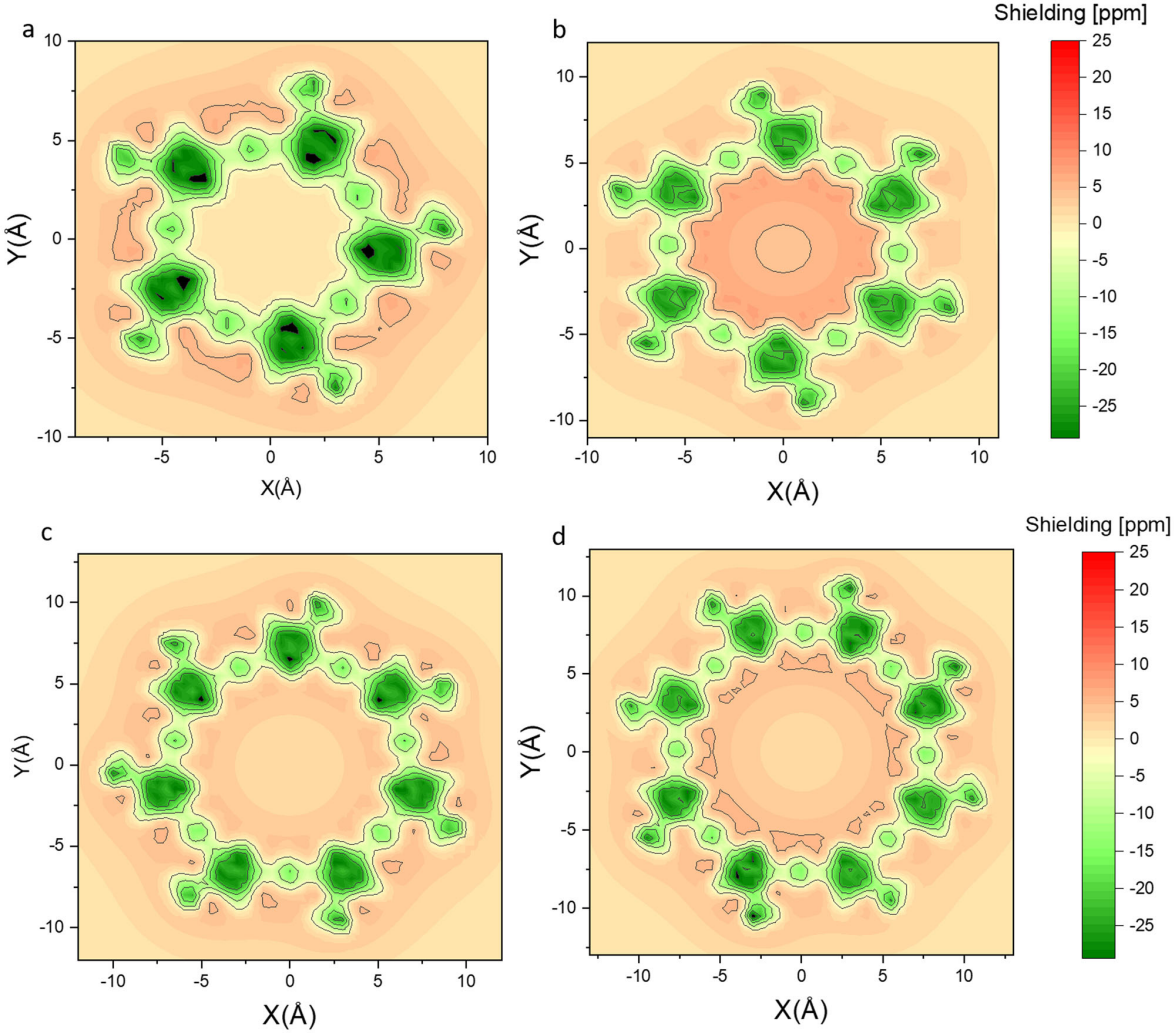
Nucleus independent chemical shift (NICS_zz_) maps of the C*m* series. NICS_zz_ maps calculated at the M06-2X/6-311 g(d) level for (**a**) **C5**, (**b**) **C6**, (**c**) **C7** and (**d**) **C8**.

To gain a better understanding of the underlying experimentally-observed trends, we evaluated the extent of the global effects by means of NICS calculations. NICS(1)_zz_ probes the ring current 1 Å above the center of the ring of interest; negative values indicate a diatropic ring current (an aromatic system), whereas positive values suggest a paratropic ring current (an antiaromatic system)^42^. We have chosen to monitor the NICS(1)_zz_ values for the furan rings, since the comparison of ring current for different ring sizes will necessarily result in different NICS values, as the distance from the dummy atom varies. First, the NICS(1)_zz_ values for each member of the **C*m*** series were calculated. Figure 9 displays a map of the zz components 1 Å above the average molecular plane. Although the effects are subtle in the neutral form, the differences between **C5,** **C6**, and **C7** are clearly observed in the interior area of the macrocycles: **C5** and **C7** show very weak aromatic (negative) values, whereas the larger red area in **C6** indicates a contribution from global antiaromatic current. The same trend also applies to **C8**, although in a lesser extent. We note that the aromatic furan units should induce an external deshielding, which can explain why the internal space of the odd-membered macrocycles is not aromatic (see Supplementary Figs. 61–64)^41^. In contrast to the NICS(1)_zz_ calculations, plots of the anisotropy of the current (induced) density (ACID) only show global ring currents for the macrocycles in their dicationic states (**C*m***^2+^). Unlike **C*m***, the NICS(1)_zz_ maps for **[*m*]CPPA** do not display any visible global aromaticity or antiaromaticity regardless of their size (see Supplementary Fig. 65).

### Size dependency of Hückel’s rule

**Fig. 10:**
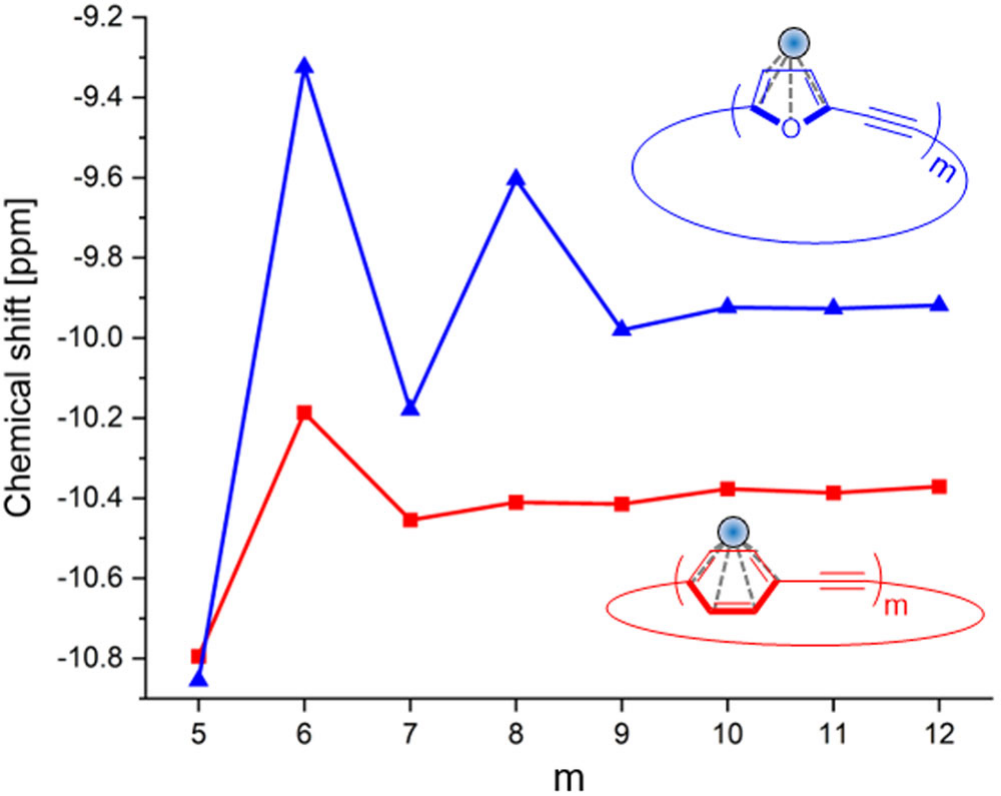
Size dependency for the attenuation of Hückel’s rule in large macrocycles. Average NICS(1)_zz_ values for the furan units in **C*m*** (blue trace) and the phenylene units in **[*m*]CPPA** (red trace), calculated at the M06-2X/6-311 G(d) level.

Although the 4*n* + 2 rule applies to small annulenes, the critical value of *n* is still debatable. For example, the aromatic stabilization energy of neutral annulenes is effective up to *n* = 6–7 (26–30 π-electrons)^6^. However, although magnetic criteria are perhaps the commonly applied tool for the quantification of aromaticity, the attenuation of chemical shifts with size was not reported for neutral macrocycles. In the current work, we show that, although the properties are clearly attenuated, alternation in magnetic and electronic properties can be observed experimentally in the **C*m*** series up to 48 π-electrons (*m* = 8). To gain insight into the rate of attenuation for sizes larger than *m* = 8, the NICS(1) values of **C*m*** members were calculated for *m* = 5–12. For comparison, we also calculated the NICS(1) for the same sized **[*m*]CPPA** series members (Fig. 10, red trace). Given that each unit of a member of the **[*m*]CPPA** series has 6 π-electrons, **[*m*]CPPA** is expected to display alternating behavior similar to that of ***Cm***. We note that attenuation with size is dependent on the functional used. It is known that π-conjugated systems suffer from over-delocalization when B3LYP is used. Following a recent discussion^43,44^, we investigated several functionals and compared the values with the experimental chemical shift. We found that M06-2X provided the best agreement with the experimental values, and therefore applied this functional in all NICS calculations (see Supplementary Table 5 and Supplementary Fig. 66 for NICS data calculated at different computational levels).

The average NICS(1) values for the furan rings are more negative for odd-members **C5** and **C7**, compared with **C6** and **C8**, indicating a stronger local aromatic character for the former. The observed alternating trend provides additional evidence of the global ring currents, even for macrocycles in the neutral form. The alternating trend decays, but still clearly persists up to *m* = 9 (54 π-electrons). This is a significantly larger value than was observed previously for neutral macrocycles in the S_0_ state. By contrast, alternation in the NICS(1) values of **[*m*]CPPA** attenuates rapidly with size, and no effective change can be observed beyond *m* = 7. The difference between the two systems can be attributed to the lower local aromaticity of furan amplifying the global properties. It is interesting to analyze these results in the context of ‘concealed aromaticity’ as recently coined by Glöcklhofer^45^. The global properties of **C*m*** are revealed predominantly upon oxidation (by their oxidation potentials and stability) and in the excited state (by fluorescence maxima), and can therefore be categorized as both type-I and type-II concealed aromaticity. However, in contrast to the **[*m*]CPPA** series, the aromatic character of **C*m*** *is* also revealed in the neutral state (by chemical shift), highlighting the importance of lowering local aromaticity to reveal global properties.

## Conclusions

In summary, we have introduced a series of π-conjugated furan-acetylene macrocycles in which the low aromaticity of furan manifests as distinct global effects in the neutral state. The odd-membered macrocycles, consisting of 4*n* + 2 π-electrons, show a global aromatic character whereas the even membered macrocycles (4*n* π-electrons) show a global antiaromatic character. These effects are expressed electronically (alternating oxidation potentials), optically (alternating emission maxima) and magnetically (alternating chemical shifts). NICS(1) calculations support the experimental observations. The differences are more pronounced for the smaller macrocycles, **C5** and **C6**, and are attenuated, yet still observable, for **C7** and **C8**. We show that the size-dependency of Hückel’s rule derives from the local aromaticity of the constituent unit. For macrocyclic furan acetylenes, this dependency can theoretically proceed up to 54 π-electrons, whereas for **[*m*]CPPA**, the alternation decays rapidly, and the effective size is 42 π-electrons. Overall, this work shows the first example of global aromaticity switching in neutral non-quinoidal macrocycles, and highlights the potential of macrocyclic furans to serve as shape persistent models for large annulenes.

## Methods

### General information

All reagents and chemicals were obtained from commercial suppliers and used as received without further purification. Flash chromatography was performed using CombiFlash SiO_2_ columns. ^1^H and ^13^C NMR spectra were recorded in solution on Brucker-AVIII 400 MHz and 500 MHz spectrometers using tetramethylsilane as the external standard. The spectra were recorded using chloroform-*d* as the solvent. Chemical shifts are expressed in *δ* units. High resolution mass spectra were measured on a high resolution quadrupole time-of-flight liquid chromatograph mass spectrometer (MS) and Waters Micromass GCT_Premier MS using electrospray ionization. Matrix-assisted laser desorption/ionization-time of flight (MALDI-TOF) spectra were acquired using an MALDI-TOF/TOF autoflex speed MS (Bruker Daltonilk GmbH, Bremen, Germany), which is equipped with a smartbeam-II solid-state laser (modified Nd:YAG laser; λ = 355 nm). The instrument was operated in positive ion, reflectron mode. The accelerating voltage was 21.0 kV. The delay time was 130 ns. Laser fluence were optimized for each sample. The laser was fired at a frequency of 2 kHz and spectra were accumulated in multiples of 500 laser shots, with 1500 shots in total. 2-[(2E)-3-(4-tert-Butylphenyl)-2-methylprop2-enylidene] malononitrile (DCTB) matrix solutions were prepared to a concentration of 20 mg mL^−1^ in dichloromethane (DCM). Sample solutions were prepared to an approximate concentration of 5 mg mL^−1^ in DCM. Sample and matrix solutions were premixed at a ratio of 1:9 or 1:40 (v/v). A volume of 1 μL of this mixture was disposed on a MALDI steel target plate. After evaporation of the solvent, the target was inserted into the mass spectrometer. Preparative size exclusion chromatography was performed in chloroform solution at room temperature using a Recycling Preparative HPLC LaboACE LC-7080 through JAIGEL-2HR and JAIGEL-2.5HR columns connected in series. 2-Bromo-3-hexylfuran (**1**) was synthesized according to literature procedures^35^. Absorption spectra were recorded on an Agilent Technologies Cary 5000 UV-Vis-NIR spectrophotometer. Fluorescence and excitation spectra were performed on Horiba Scientific Fluoromax-4 spectrofluorometer. All spectroscopic experiments were performed using standard quartz cuvettes of path length 1 cm and solutions prepared in spectroscopic grade solvents. The excitation laser used was 330 nm with a pulse width of less than 1.4 ns. The synthetic pathway for **4** is described in the Supplementary Synthesis section (Supplementary Fig. 1). NMR and MS spectra are provided for all compounds: see Supplementary Figs. 2–37. Absorption and emission spectra for **C5**-**C8** are provided in Supplementary Figs. 38–46. Circular voltammograms are provided in Supplementary Figs. 47–50. Calculated chemical shifts, ACID, NICS, and VIST plots for **C5**-**C8** are provided in Supplementary Figs. 51–71. Optimization conditions for macrocyclization are provided in Supplementary Table 1. Photophysical properties for **C5**-**C8** are provided in Supplementary Table 2. Calculated formation energies and MO energies are provided in Supplementary Tables 3–4. Calculated NICS values, strain energies and emission spectra are provided in Supplementary Tables 5–7, respectively. Crystallographic data for **C6** and **C7** is provided in Supplementary Table 8.

### Synthetic procedure for **C*m***

2-Ethynyl-3-hexyl-5-iodo-furan (**4**) (1 g, 3.31 mmol), tetrakis(triphenylphosphine)palladium (390 mg, 10%mol) and CuI (18 mg, 3%mol) were dissolved in a mixture of 75 mL of dry toluene and 75 mL of distilled N,N-diisopropylethylamine under an inert atmosphere, heated to 60 °C, and the reaction mixture was stirred for 3 days. Afterward, the reaction mixture was allowed to reach room temperature and was subsequently filtered over celite. The filtrate was evaporated and the residue obtained was purified by flash column chromatography on silica gel using a mixture of DCM and hexane (9:1) as eluent to give a mixture of macrocycles. The mixture was separated using gel permutation chromatography and chloroform as the eluent to give **C5** (1.38 mg, <1%), **C6** (10.4 mg, 10.5%), **C7** (10.9 mg, 13%), and **C8** (8.5 mg, 11.5%).

## Supplementary information

Supplementary Information

Description of Additional Supplementary Files

Supplementary Data 1

Supplementary Data 2

Supplementary Data 3

## Data Availability

Crystallographic data for the structures reported in this article have been deposited at the Cambridge Crystallographic Data Center, under deposition numbers CCDC 2191813 (**C6**) and 2191812 (**C7**). Copies of the data can be obtained free of charge via https://www.ccdc.cam.ac.uk/structures/. Synthetic and characterization data for all reported compounds as well as computational details are included in the Supplementary Information file under “Supplementary Methods”. Cartesian coordinates for calculated structures are included in Supplementary Data 1. The crystallographic information file (CIF) for C6 and C7 are included in Supplementary Data 2 and 3, respectively.
