# Supplementary material for: Alternating behavior in furan-acetylene macrocycles reveals the size-dependency of Hückel’s rule in neutral molecules
Source: Commun Chem. 2023 May 27;6:100. doi: 10.1038/s42004-023-00902-9 (PMC10224948; doi:10.1038/s42004-023-00902-9)
Supplement: Supplementary file 1 — Supplementary Information [file 42004_2023_902_MOESM1_ESM.pdf]

Alternating behavior in furan-acetylene macrocycles reveals the size-dependency of Hückel's rule in neutral molecules

Yuval Rahav‡, Shinaj K. Rajagopal‡, Or Dishi, Benny Bogoslavsky and Ori Gidron*

†*Institute of Chemistry, The Center for Nanoscience and Nanotechnology, Casali Center for Applied Chemistry, The Hebrew University of Jerusalem, Edmond J. Safra Campus, Jerusalem 9190401, Israel.*
*E-mail: ori.gidron@mail.huji.ac.il
‡These authors contributed equally.

Supporting Information (SI)

Table of Content

Table of Contents

S1. General Information 3
S.1.1 Materials and Methods 3
S.1.2. Spectral Measurements 3
S2. Synthesis 4
S2.1 Synthetic scheme 4
S2.2 Procedures 4
S3. Spectra 9
S3.1. NMR 9
S3.2 MALDI-TOF 41
S3.3. Experimental Absorption, Emission, and Excitation Spectra 45
S4. Electrochemistry 51
S5. Computational Results 55
S5.1. Absolute Energies and Comparison of Different Functionals 55
S5.2ACID plots 58
S5.3. NICS 66
S.5.4 VIST 75
S5.4. Strain Energies 75
S5.5. TD spectra 78
S6. X-Ray Crystallography 79
S6.1. Calculation of the Interplanar Angles 80
References 80

Supplementary Methods

S1. General Information

S.1.1 Materials and Methods

All reagents and chemicals were obtained from commercial suppliers and used as received without further purification. Flash chromatography (FC) was performed using CombiFlash SiO_2 columns. ^1H and ^{13}C NMR spectra were recorded in solution on a Brucker-AVIII 400 MHz and 500 MHz spectrometers using tetramethylsilane (TMS) as the external standard. The spectra were recorded using chloroform-d as the solvent. Chemical shifts are expressed in δ units. High resolution mass spectra were measured on a HR Q-TOF LCMS and Waters Micromass GCT_Premier Mass Spectrometer using ESI. MALDI-TOF MS spectra were acquired using an MALDI-TOF/TOF autoflex speed mass spectrometer (Bruker Daltonilk GmbH, Bremen, Germany), which is equipped with a smartbeam-II solid-state laser (modified Nd:YAG laser) λ = 355 nm. The instrument was operated in positive ion, reflectron mode. The accelerating voltage was 21.0 kV. The delay time was 130 ns. Laser fluence were optimized for each sample. The laser was fired at a frequency of 2 kilohertz and spectra were accumulated in multiples of 500 laser shots, with 1500 shots in total. Sample preparation: 2-[(2E)-3-(4-tert-Butylphenyl)-2-methylprop2-enylidene] malononitrile (DCTB) matrix solutions were made to a concentration of 20 mg/ml in dichloromethane (DCM). Sample solutions were made to an approximate concentration of 5 mg/ml in DCM. Sample and matrix solutions were premixed in ratio of 1:9 or 1:40 (v/v). A volume of 1 μl of this mixture was disposed on MALDI steel target plate. After evaporation of the solvent the target was inserted into mass spectrometer. Preparative size exclusion chromatography was performed in chloroform solution at room temperature using a Recycling Preparative HPLC LaboACE LC-7080 through JAIGEL-2HR and JAIGEL-2.5HR columns connected in series. 2-bromo-3-hexylfuran (**1**) was synthesized according to literature procedures.[1]

S.1.2. Spectral Measurements

Absorption spectra were recorded on Agilent Technologies Cary 5000 UV-Vis-NIR spectrophotometer while fluorescence and excitation spectra were performed on Horiba Scientific Fluoromax-4 spectrofluorometer respectively. All spectroscopic experiments were performed by using standard quartz cuvettes of path length 1cm for solution in spectroscopic grade solvents. The excitation laser used is 330 nm with a pulse width of less than 1.4 ns.

S2. Synthesis

S2.1 Synthetic scheme

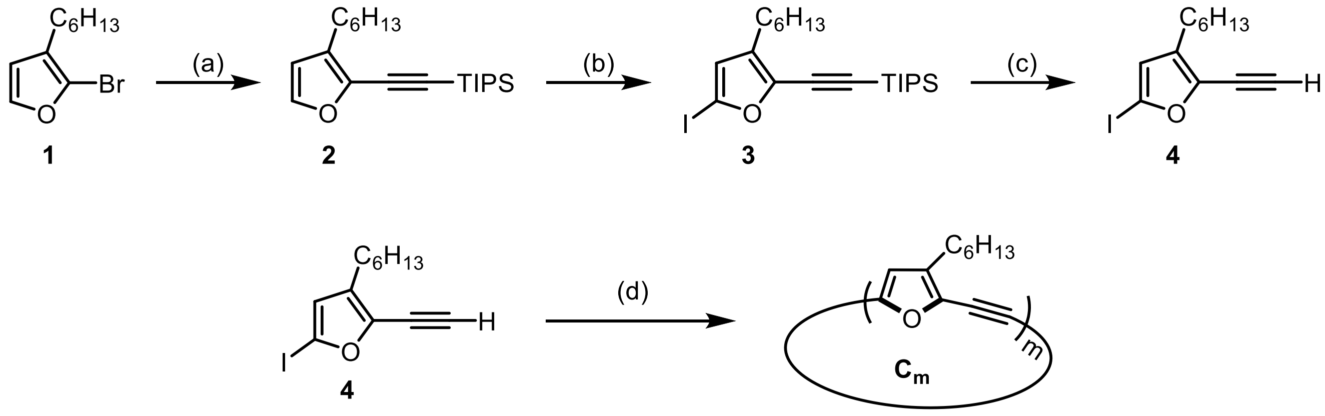

m = 5, <1%
m = 6, 10%
m = 7, 13%
m = 8, 11%

Supplementary Figure 1. Synthesis of **Cm**. a) triisopropyl silyl (TIPS) acetylene, Bis(triphenylphosphine)palladium(II) dichloride ($Pd(PPh_3)_2Cl_2$), Et_3N, reflux, 2d; b) I_2 (3 eq), lithium diisopropylaminde (LDA) (1M), tetrahydrofuran (THF), -78 °C, c) tetra-*n*-butylammonium fluoride (1M), THF, 3h; d) Pd(PPh3)4 (10% mol), CuI (3% mol), toluene:diisopropylethylamine (DIPEA) (1:1), 60 °C, 3d.

S2.2 Procedures

Synthesis of 3-hexyl-2-triisopropylsillylfuran (2)

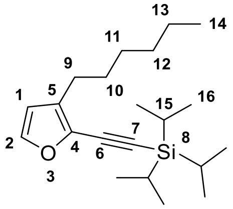

CuI (325 mg, 1.7 mmol), $Pd(PPh_3)_2Cl_2$ (625 mg, 0.9 mmol) and **1** (6.01 g, 26.1 mmol) were dissolved in 36 mL of dry Et_3N under an inert atmosphere with a reflux condenser attached, and the mixture was heated to 80 °C. After the target temperature was obtained triisopropylsilyl (TIPS) acetylene (10 mL, 44.5mmol) was added and the reaction was heated to reflux and left to stir for 48 hours. The reaction mixture was then allowed to cool to room temperature and was then filtered over celite. The filtrate was concentrated and the residue was and purified by column chromatography on silica gel using hexane the eluent to give **2** as a yellowish liquid (7 g. 78%). NMR (400MHz, $CDCl_3$) δ 7.24(d, J = 1.9Hz, 1H, H-C(2)), 6.26(d, J = 1.9Hz, 1H, H-C(1)), 2.49 (t, J = 7.6Hz, 2H, H-C(9)), 1.52-1.60 (m, 2H, H-C(10)), 1.24-1.31 (m, 6H, H-C(11-13)), 1.09-1.15 (m, 21H, H-C15,C16)) 0.85-0.89 (m, 3H). ^{13}C-NMR (126MHz, $CDCl_3$) δ142.55 (C2), 134.55 (C4), 131.79 (C5), 111.86 (C1) , 98.09 (C7), 95.92 (C6), 31.59, 29.75, 28.86, 25.39 (C9), 22.60, 18.64, 14.06, 11.24 (C14). HRMS (ESI) calculated for $C_{21}H_{36}OSi$ 333.2608, found 333.2604 $(M+H)^+$.

Synthesis of 3-hexyl-5-iodo-2-triisopropylsillylfuran (3)

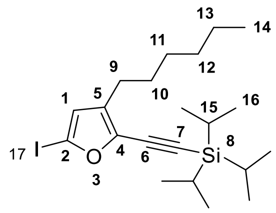

Lithium diisopropylamine 2M (LDA, 36 mL, 72 mmol) was added to 400 mL of dry tetrahydrofuran (THF) at -78 °C under inert atmosphere and stirred for 10 minutes. **2** (6.67 g, 20.3 mmol) dissolved in 50 ml of dry THF was added dropwise to the reaction mixture over 1 hour. After the addition was completed, the mixture was left to stir for 1.5 additional hours, during which the reaction turned yellow. Iodine (22.2 g, 86.6 mmol) was dissolved in 110 mL of dry THF and then added dropwise over 1 hour while maintaining the temperature (-78 °C). The resulting brownish solution was left to stir for 3 more hours at -78 °C and then allowed to reach room temperature overnight while stirring. The reaction mixture was quenched using a saturated solution of $Na_2S_2O_3$ stirred for 2 hours at room temperature. Then, a saturated solution of $NHCl_4$ was added and aqueous phase was separated and washed with diethyl ether (4 x 60 mL). The organic phase was combined, dried with Na_2SO_4 and concentrated. The residue obtained was purified by flash column chromatography on silica gel using hexane the eluent to give **3** as a brown liquid (6.5 g, 70%). ^{1}H-NMR (400MHz, $CDCl_3$) δ 6.42 (s, 1H, H-C(1)), 2.45 (t, J = 7.4Hz, 2H, H-C(9)), 1.47-1.57 (m, 2H, H-C(10)), 1.24-1.32 (m, 6H, H-C(11-13)) 1.09-1.12 (m, 21H, H-C(15,16)), 0.84-0.89 (m, 3H, H-C(14)). ^{13}C-NMR (126MHz, $CDCl_3$) δ140.10 (C4) 134.63 (C5), 122.49 (C1) , 100.28 (C7), 94.57 (C6), 87.89 (C2), 31.54, 29.55, 28.77, 25.27, 22.57, 18.62, 14.06, 11.21. HRMS (ESI) calculated for $C_{21}H_{35}IOSi$ 459.1575 , found 459.1571 $(M+H)^+$.

Synthesis of 3-hexyl-5-iodo-2-acetylenefuran (4)

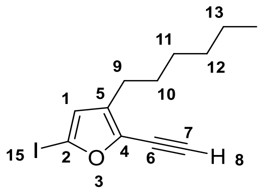

3 (6.5 g, 14.19 mmol) was dissolved in 280 mL of dry THF cooled to 0 °C. 20 mL of tetra-*n*-butylammonium fluoride (1M in THF, 20 mmol) were added and the solution was stirred 2 hours while maintaining the temperature using an ice bath. The reaction mixture was quenched using a saturated solution of NH_4Cl and aqueous phase was separated and washed with diethyl ether (4 x 40 mL). The organic phase was combined, dried with Na_2SO_4 and concentrated and the residue obtained was purified by flash column chromatography on silica gel using hexane the eluent to give **4** as a dark-red liquid (3.5 g, 82%). ^{1}H-NMR (400MHz, $CDCl_3$) δ 6.44 (s, 1H, H-C(1)), 3.60 (s, 1H, H-C(7)), 2.45 (t, J = 7.5, 2H, H-C(9)), 1.49-1.56 (2H, H-C(10)), 1.26-1.33 (m, 6H, H-C(11-13)), 0.86-0.90 (m, 3H, H-C(14)). ^{13}C-NMR (126MHz, $CDCl_3$) δ138.77 (C2), 135.01 (C4), 122.56 (C5), 88.91 (C1), 85.76 (C7), 72.65 (C6), 31.65, 29.57, 28.87, 25.09 (C9), 22.71, 14.21 (C14). HRMS (ESI) calculated for $C_{12}H_{15}IO$ 303.0240 , found 303.0244 $(M+H)^+$.

Synthesis of Cm

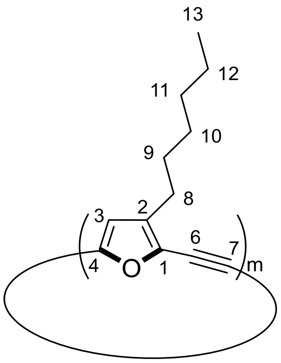

4 (1 g, 3.31 mmol), $Pd(PPh_3)_4$ (390 mg, 10%mol) and CuI (18 mg, 3%mol) were dissolved in a mixture of 75 mL of dry toluene and 75 mL of distilled N,N-diisopropylethylamine (DIPEA) under an inert atmosphere, heated to 60 °C, and the reaction mixture was stirred for 3 days. Afterward, the reaction mixture was allowed to reach room temperature and was subsequently filtered over celite. The filtrate was evaporated and the residue obtained was purified by flash column chromatography on silica gel using a mixture of dichloromethane and hexane (9:1) as eluent to give a mixture of macrocycles. The mixture was separated using gel permutation chromatography (GPC) and chloroform as the eluent to give **C5** (1.38 mg, <1%), **C6** (10.4 mg, 10.5%), **C7** (10.9 mg, 13%), and **C8** (8.5 mg, 11.5%).

C5:

NMR (400MHz, $CDCl_3$) δ6.69(s, 1H, H-(C3))), 2.65 (t, J=7.6Hz, 2H, H-(C8)), 1.61-1.71 (m, 6H, H-(C10-12)), 0.88 (m, 3H, H-(C13)**)).** ^{13}C-NMR (126MHz, $CDCl_3$) δ137.82 (C4), 135.22 (C1), 130.50 (C2), 114.37 (C3), 89.07 (C7), 86.73 (C6), 31.75, 29.86, 28.93, 25.18, 22.80, 14.27. MALDI-TOF calculated for $C_{60}H_{70}O_5$ 870.527, found 870.548(M^+).

C6:

^{1}H-NMR (400MHz, $CDCl_3$) δ6.41 (s, 1H, H-(C3))), 2.44 (t, J=7.5Hz, 2H, H-(C8)), 1.51-1.60 (m,2H, H-(C9)), 1.25-1.35 (m, 6H, H-(C10-12)), 0.86-0.90(m, 3H, H-(C13)). ^{13}C-NMR (126MHz, $CDCl_3$) δ137.78 (C4), 135.29 (C1), 131.97 (C2), 115.99 (C3), 87.65 (C7), 85.05 (C6), 31.70, 29.77, 28.87, 25.27, 22.72, 14.25. MALDI-TOF calculated for $C_{72}H_{84}O_6$ 1044.626, found 1044.626 (M^+).

C7:

^{1}H-NMR (400MHz, $CDCl_3$**)** δ6.58 (s, 1H, H-C(3)), 2.52 (t, 7.5Hz, 2H, H-(C8), 1.56-1.63 (m, 2H, H-(C9)), 1.28-1.38 (m, 6H, H-(C10-12)), 0.86-0.91 (m, 3H, H-(C13)). ^{13}C-NMR (126MHz, $CDCl_3$) δ137.39 (C4), 135.01 (C1), 132.88 (C2), 117.06 (C3), 86.94 (C7), 84.19 (C6), 31.70, 29.78, 28.89, 25.37, 22.72, 14.25. MALDI-TOF calculated for $C_{84}H_{98}O_7$ 1218.729, found 1218.729 (M^+).

C8:

^{1}H-NMR (400MHz, $CDCl_3$) δ6.58 (s, 1H, H-(C3)), 2.50 (t, J=7.5Hz, 2H, H-(C8)), 1.55-1.61 (m, 2H, H-(C9)), 1.27-1.37 (m, 6H, H-(C10-12)), 0.87-0.90 (m, 3H, H-(C13)). ^{13}C-NMR (126MHz, $CDCl_3$) δ136.11 (C4), 133.78 (C1), 132.39, 116.71

(C2), 85.37 (C7), 82.7 (C6), 30.52, 28.68, 28.49, 27.71, 24.18, 21.55. MALDI-TOF calculated for $C_{96}H_{112}O_8$ 1393.839, found 1393.843 (M^+).

Supplementary Table1. Optimization reaction conditions for macrocyclization of **4**. DCM ≡ dichloromethane, THF ≡ tetrahydrofuran, TBAF ≡ tetra-*n*-butylammonium fluoride, DIPEA ≡ diisopropylethylamine.

	**Reaction conditions**	**Molarity**	**Time**	**Remarks**
RXN-1	Pd(0)[3.5 mol%], CuI [3 mol%], Et_3N, DCM, RT	100 mg, 8 mM<br>20 mL : 20 mL Et_3N : DCM	2 days	No reaction
RXN-2	Pd(0)[3.5 mol%], CuI [3 mol%], Et_3N, THF, RT	100 mg, 8 mM<br>20 mL : 20 mL Et_3N : THF	2 days	2% yield of **C5**, **C6**, **C7**, **C8**
RXN-3	Pd(0)[3.5 mol%], CuI [3 mol%], Et_3N, Toluene, RT	100 mg. 3 mM<br>50 mL : 50 mL Et_3N : Toluene	2 days	5% yield of **C6**, **C7**, **C8**
RXN-4	Pd(0)[3.5 mol%], CuI [3 mol%], Et_3N, DCM, RT	100 mg, 3 mM<br>50 mL : 50 mL Et_3N : THF	2 days	No reaction
RXN-5	Pd(0)[3.5 mol%], CuI [3 mol%], Et_3N, DCM, RT	100 mg, 3 mM<br>50 mL : 50 mL Et_3N : DCM	2 days	No reaction
RXN-6	Pd(0)[3.5 mol%], CuI [3 mol%], Et_3N, DCM, 40 °C	100 mg, 3 mM<br>50 mL : 50 mL Et_3N : DCM	2 days	To the mixture of 50 mL of Et3N and 40 mL of DCM maintained at 40°C, was added dropwise a solution of the monomer (4) (100 mg dissolved in 10 mL of DCM) – No reaction
RXN-7	Pd(0)[3.5 mol%], CuI [3 mol%], Et_3N, RT	100 mg, 8 mM<br>40 mL Et_3N	2 days	No reaction
RXN-8	Pd(0)[3.5 mol%], CuI [3 mol%], Et_3N, RT → 60 °C	100 mg, 1 mM<br>75 mL : 75 mL Et_3N : THF	3 days	TBAF dropwise – deprotection in situ (6 hours), only linear polymer observed
RXN-9	Pd(0)[3.5 mol%], CuI [3 mol%], Et_3N, 30 °C-35 °C	100 mg, 1 mM<br>75 mL : 75 mL Et_3N : DCM		Dropwise addition (2.5 hours)<br>No reaction
RNX-10	Pd(0)[15 mol%], CuI [3 mol%], DIPEA, Toluene, RT	106 mg, 1 mM<br>75 mL : 75 mL DIPEA : Toluene	4 days	Glovebox – No reaction
RNX-11	Pd(0)[10 mole %], CuI [3 mole %], DIPEA, Toluene, RT	10 mg, 2.2 mM<br>7.5mL:7.5mL – DIPEA : Toluene	4 days	Glovebox – No reaction
RNX-12	Pd(0)[10 mol%], CuI [3 mol%], DIPEA, Toluene, RT → 60 °C	20 mg, 22 mM<br>1.5 mL : 1.5mL DIPEA : Toluene	6 days	4 days in Glovebox – no reaction, heating to 60°C for 2 days in hood – 2mg of mixture crude
RNX-13	Pd(0)[10 mol%], CuI [3 mol%], DIPEA, Toluene, RT	10 mg, 2.2 mM<br>7.5 mL : 7.5mL DIPEA : DCM	4 days	Glovebox – No reaction
RNX-14	Pd(0)[10 mol%], CuI [3 mol%], DIPEA, Toluene, RT → 60 °C	20 mg, 22 mM<br>1.5 mL : 1.5 mL DCM : Toluene	6 days	4 days in Glovebox – no reaction, heating to 60°C for 2 days outside the glovebox – still no reaction
RNX-15	Pd(0)[10 mol%], CuI [3 mol%], DIPEA, Toluene, RT	100 mg, 22 mM<br>7.5 mL : 7.5 mL DIPEA : Toluene	2 days	30% yield for mixture of **C6**, **C7**, **C8**

RNX-16	Pd(0)[10 mol%], CuI [3 mol%], DIPEA, Toluene, 60 °C	100 mg, 22 mM<br>7.5 mL : 7.5 mL DIPEA: Toluene	2 days	Reaction in hood under inert conditions; 30% yield for mixture of **C6**, **C7**, **C8**
RNX-17	Pd(0)[10 mol%], CuI [3 mol%], DIPEA, Toluene, 60 °C	100 mg, 2.2 mM<br>50 mL : 50 mL DIPEA : Toluene	2 days	No reaction
RNX-18	Pd(0)[10 mol%], CuI [3 mol%], DIPEA, Toluene, 80 °C	1 g, 22 mM<br>75 mL : 75 mL DIPEA : Toluene	2 days	25% Yield for mixture of **C6**, **C7**, **C8**
RNX-19	Pd(0)[10 mol%], CuI [3 mol%], DIPEA, Toluene, 60 °C	1 g, 22 mM<br>75 mL : 75 mL DIPEA: Toluene	4 days	Optimal conditions, 33% yield for mixture of **C5**, **C6**, **C7**, **C8**

S3. Spectra

S3.1. NMR

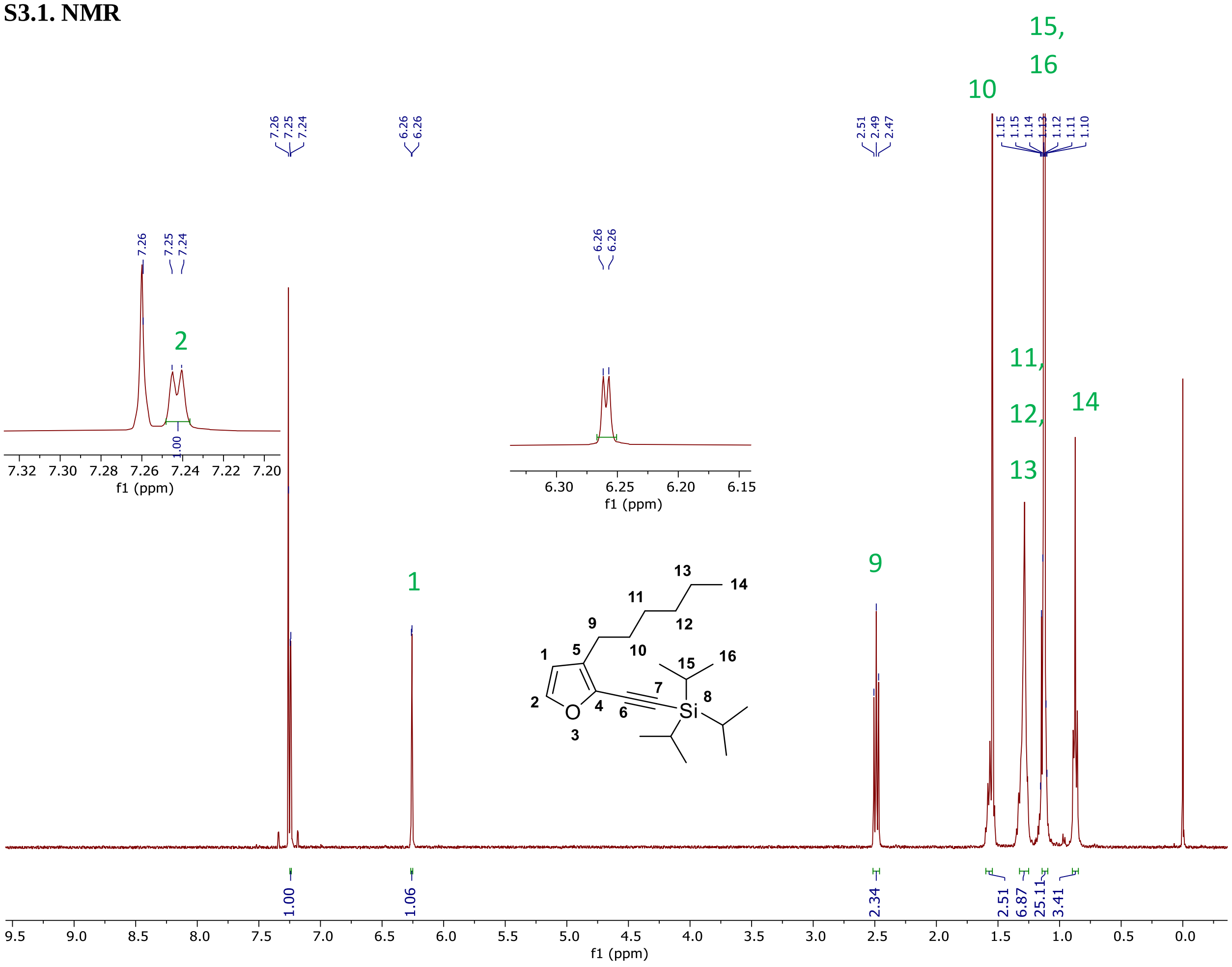

Supplementary Figure 2. ^{1}H-NMR spectrum of **2** in $CDCl_3$ measured at 298 K.

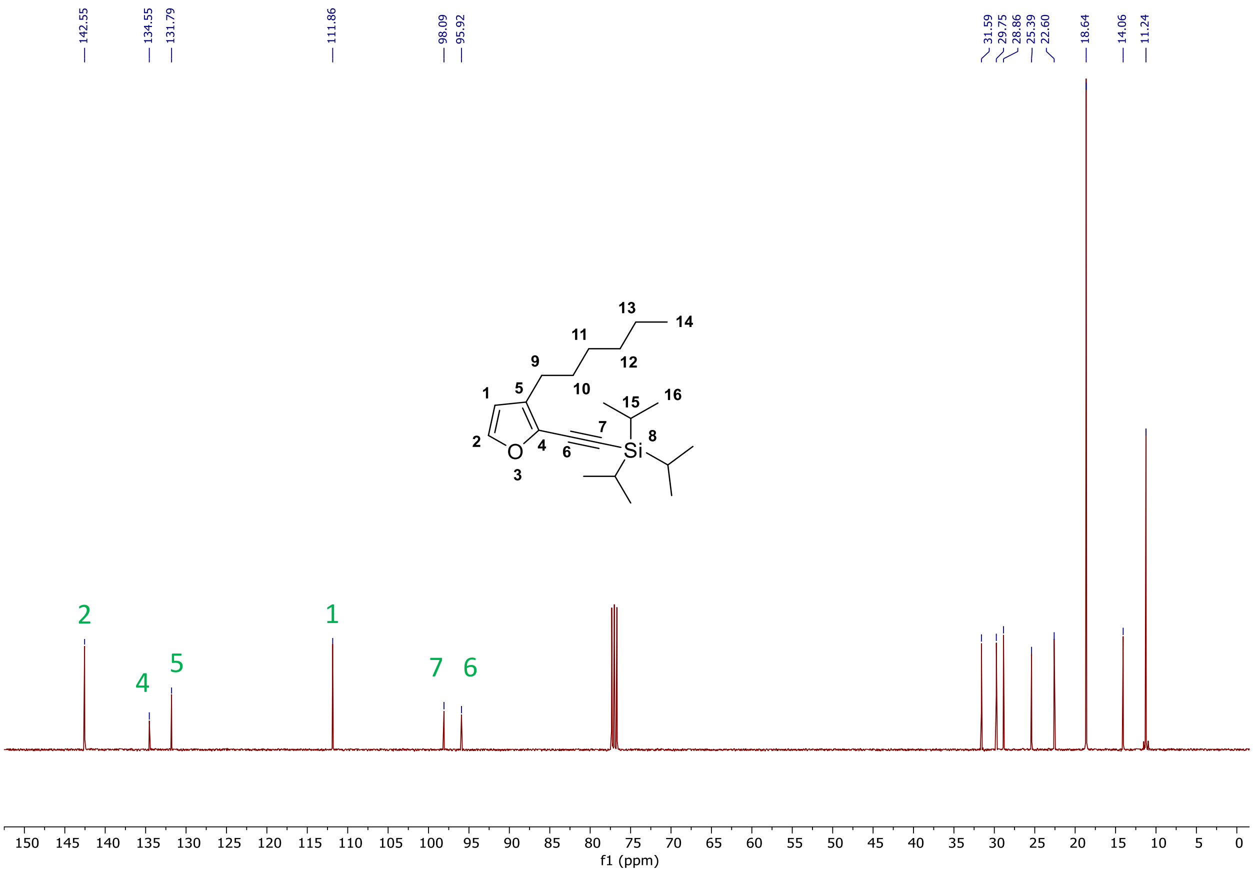

Supplementary Figure 3. ^{13}C-NMR spectrum of **2** in $CDCl_3$ measured at 298 K.

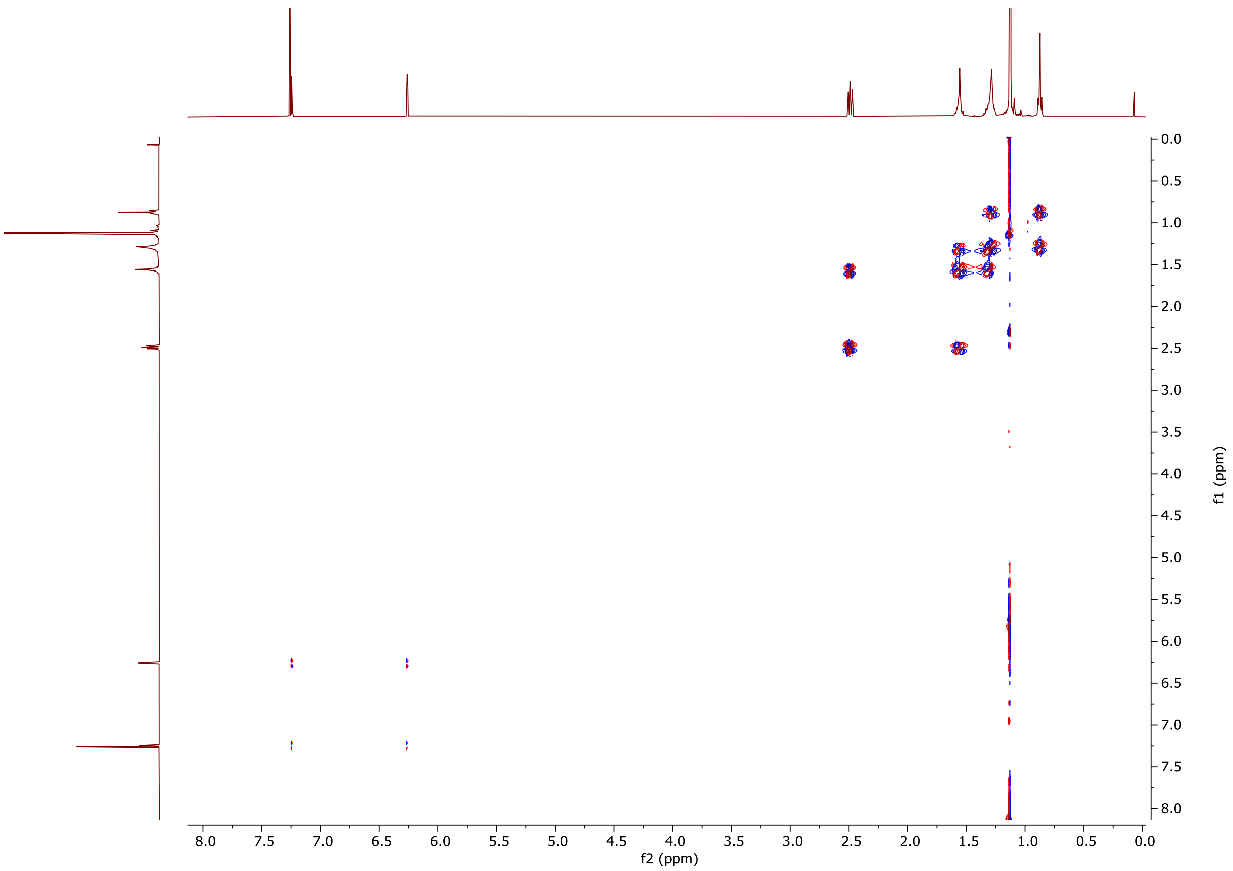

Supplementary Figure 4. COSY-NMR spectrum of **2** in $CDCl_3$ measured at 298 K.

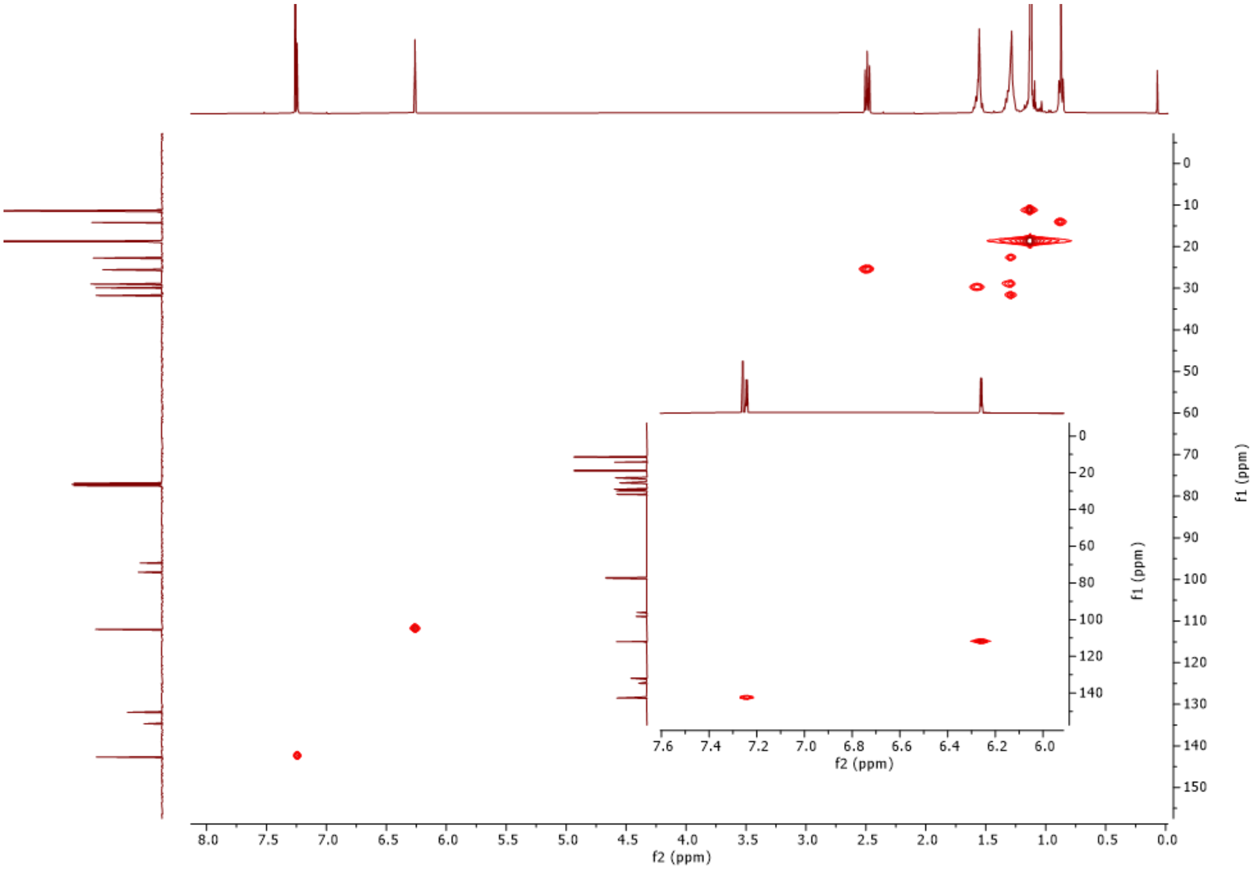

Supplementary Figure 5. HSQC-NMR spectrum of **2** in $CDCl_3$ measured at 298 K.

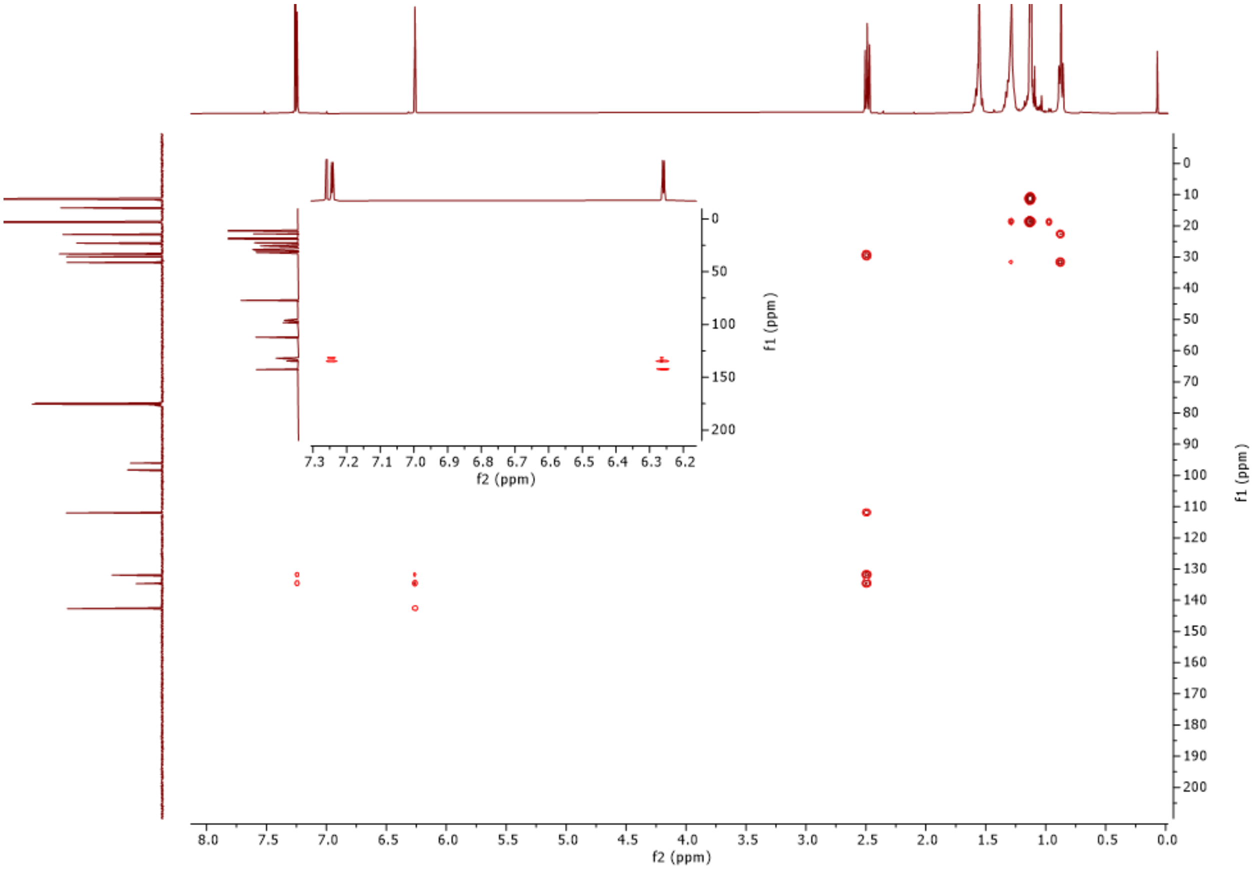

Supplementary Figure 6. HMBC-NMR spectrum of **2** in $CDCl_3$ measured at 298 K.

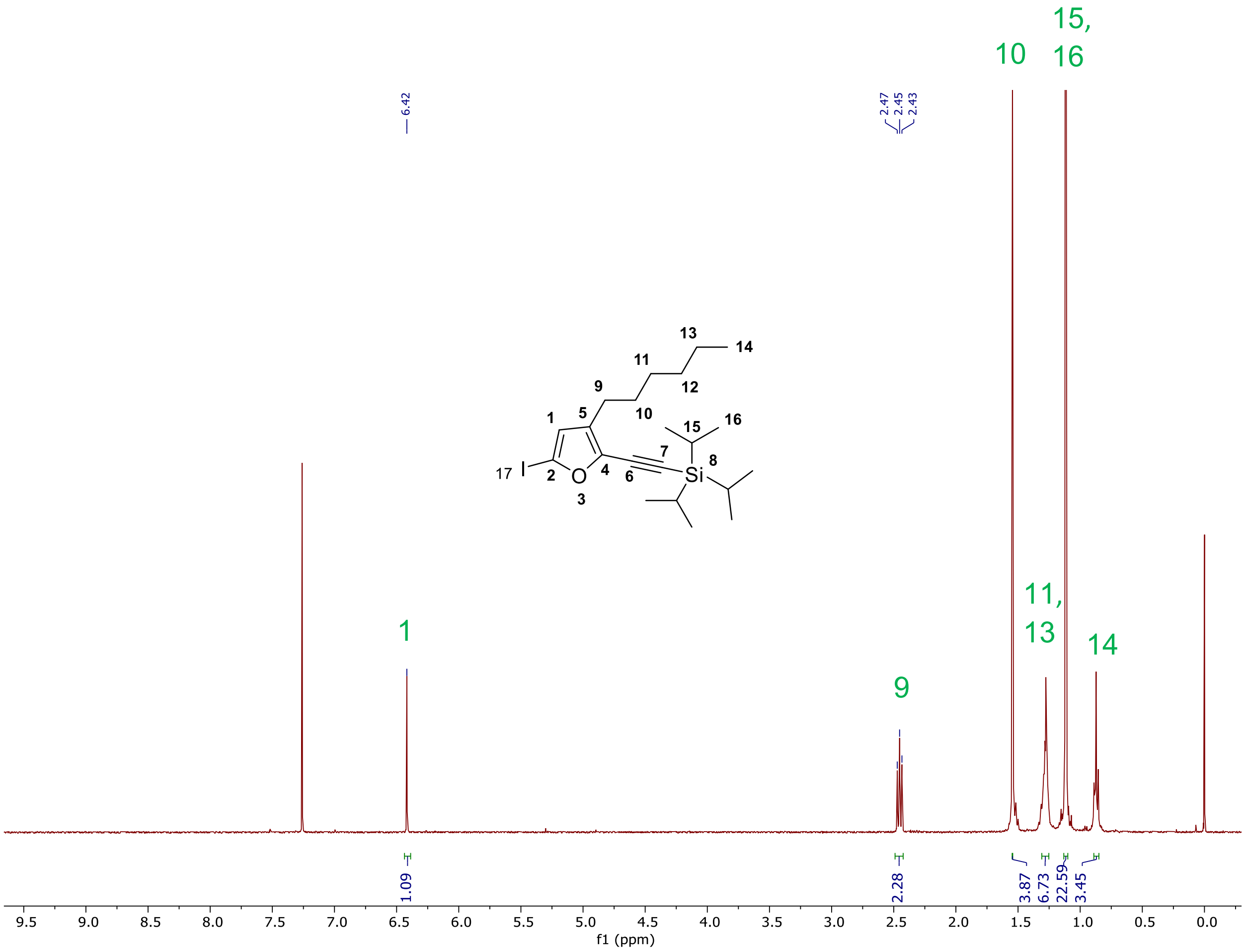

Supplementary Figure 7. ^{1}H-NMR spectrum of **3** in $CDCl_3$ measured at 298 K.

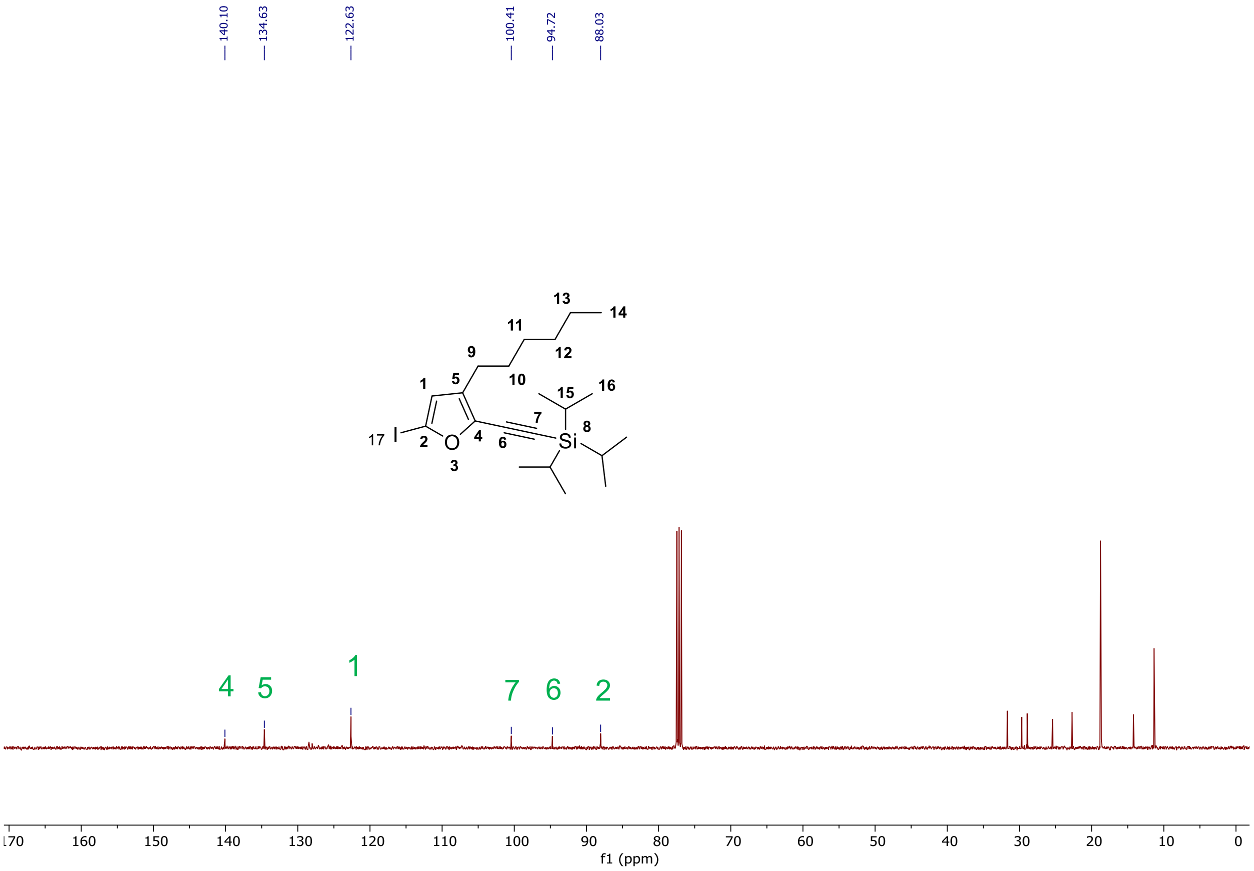

Supplementary Figure 8. ^{13}C-NMR spectrum of **3** in $CDCl_3$ measured at 298 K.

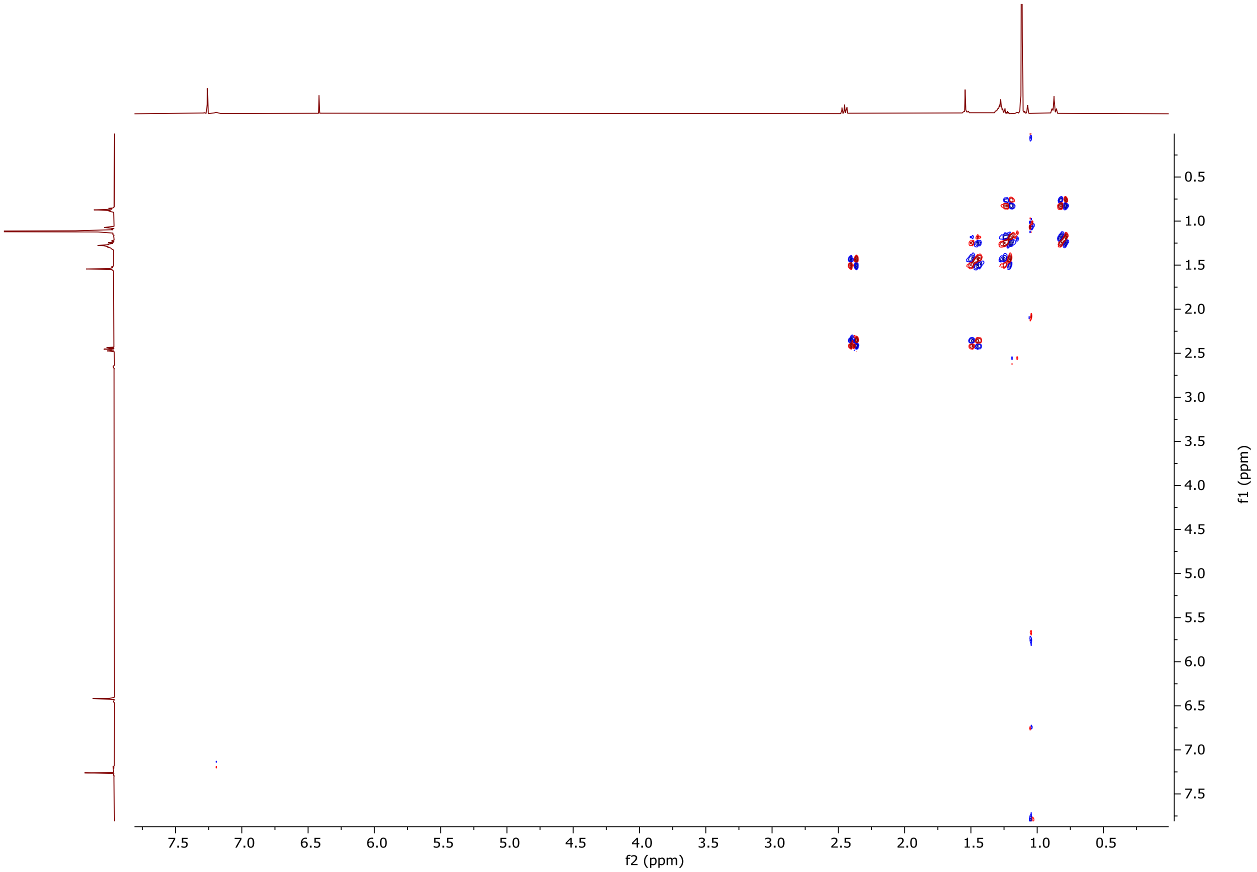

Supplementary Figure 9. COSY-NMR spectrum of **3** in $CDCl_3$ measured at 298 K.

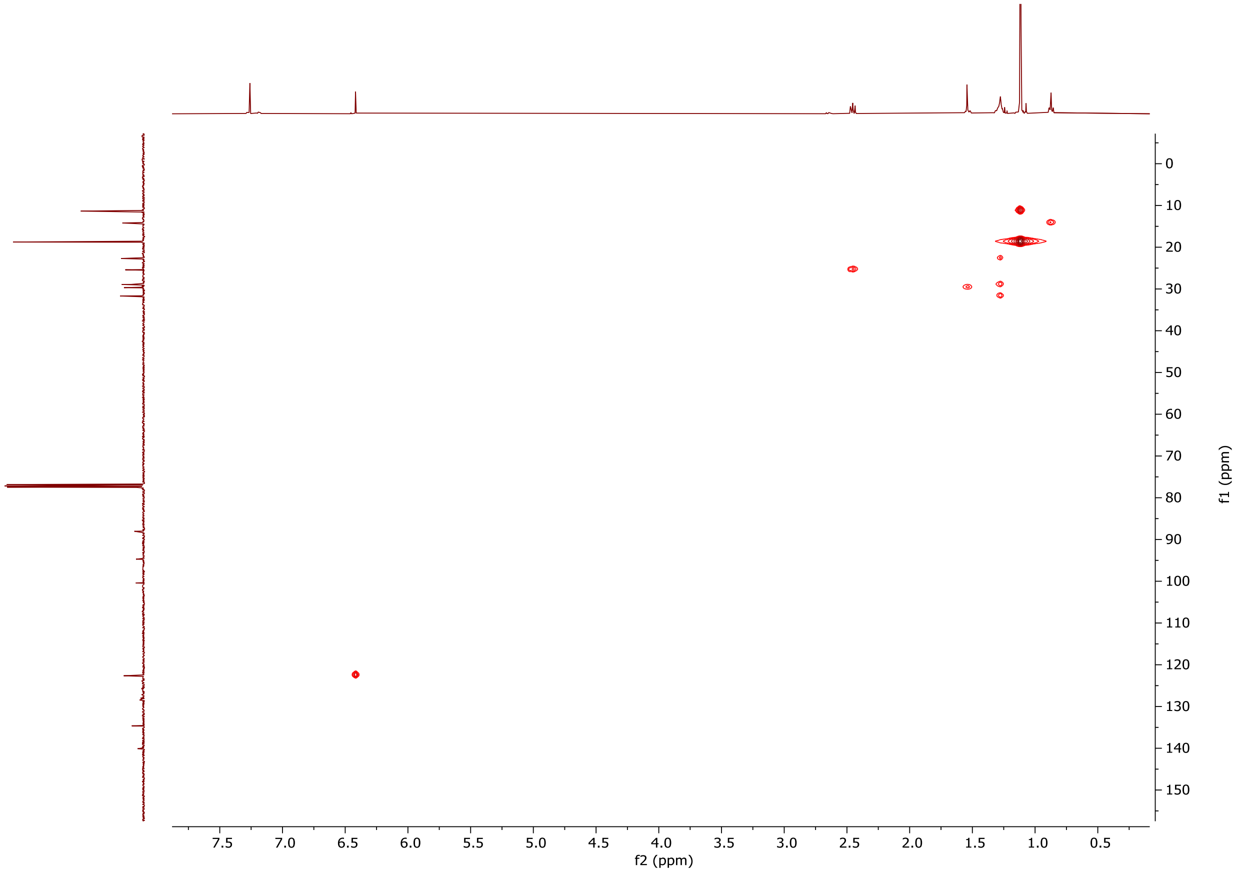

Supplementary Figure 10. HSQC-NMR spectrum of **3** in $CDCl_3$ measured at 298 K.

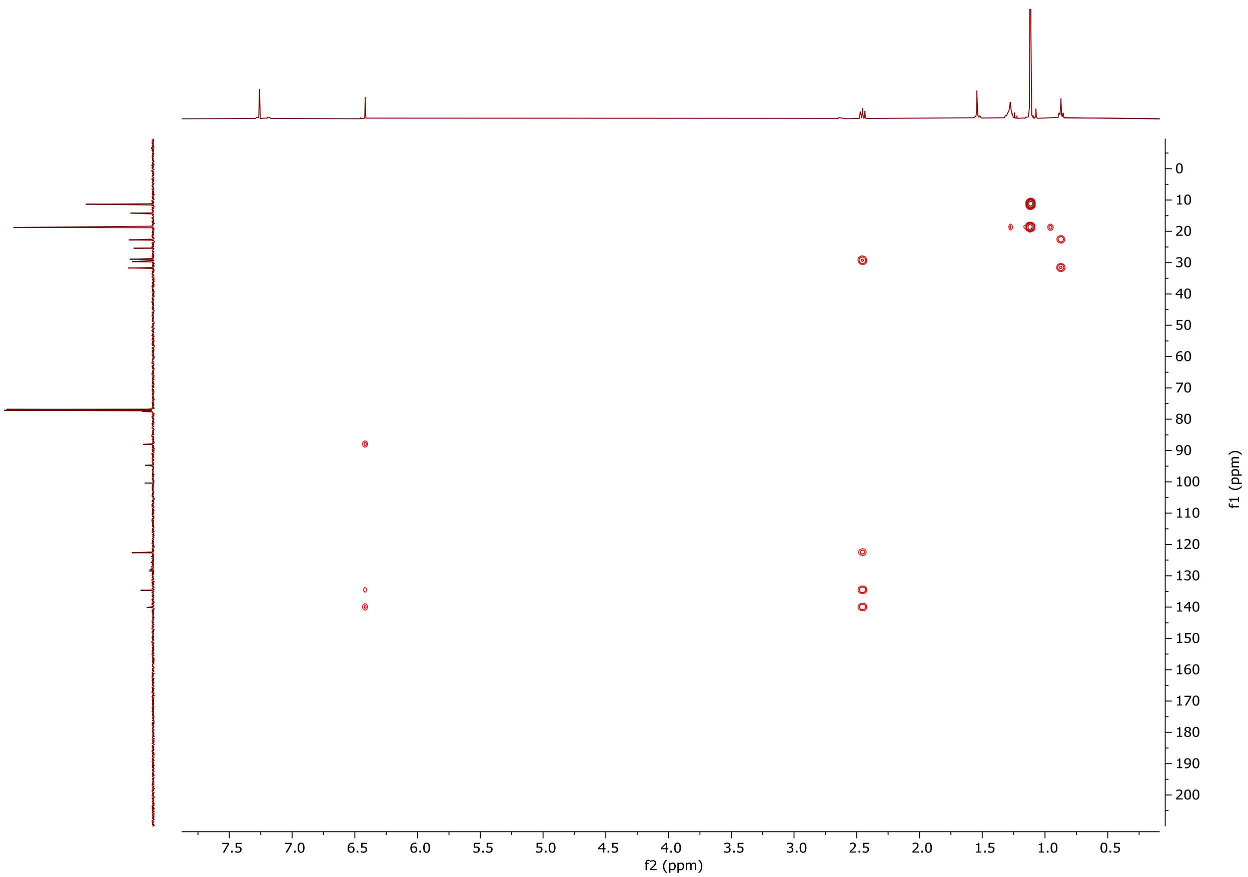

Supplementary Figure 11. HMBC-NMR spectrum of **3** in $CDCl_3$ measured at 298 K.

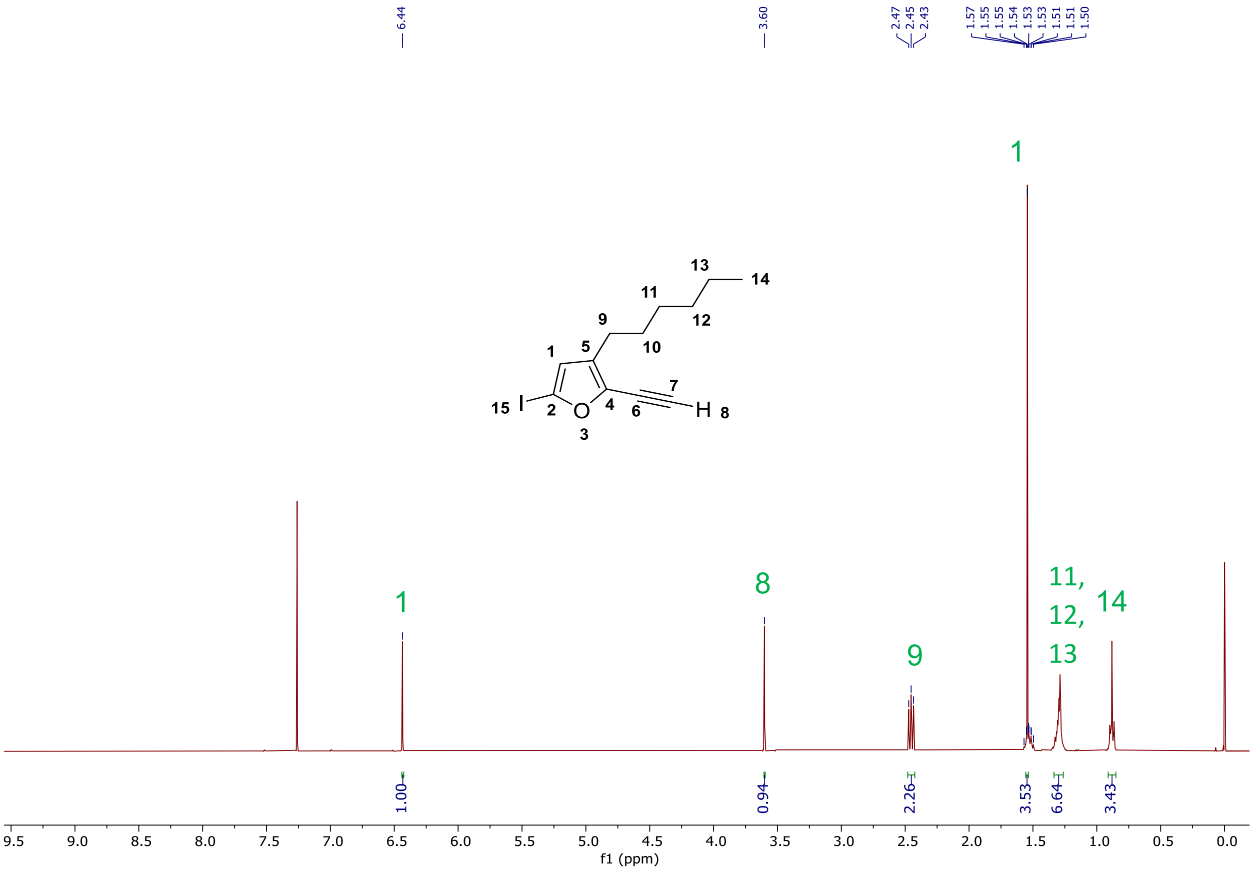

Supplementary Figure 12. ^{1}H-NMR spectrum of **4** in $CDCl_3$ measured at 298 K.

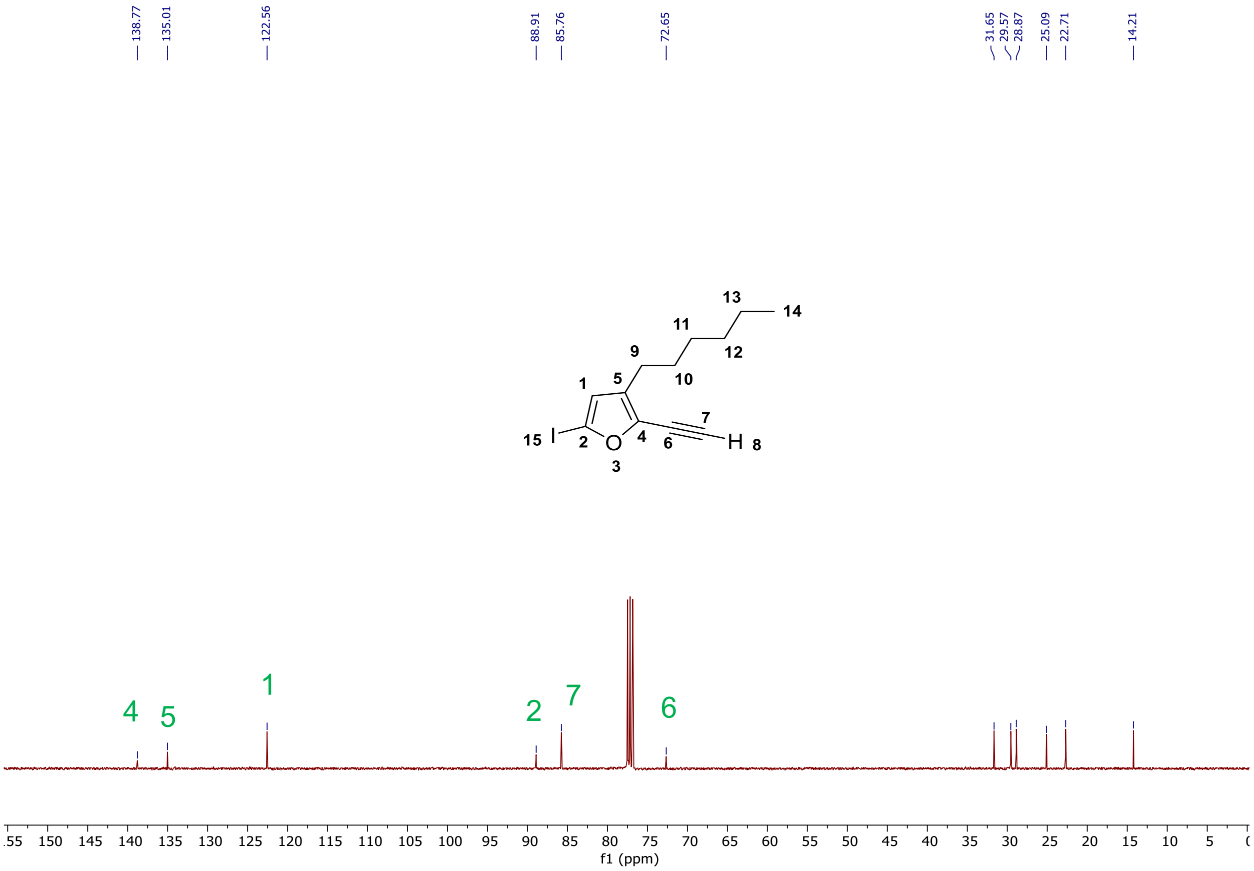

Supplementary Figure 13. ^{13}C-NMR spectrum of **4** in $CDCl_3$ measured at 298 K.

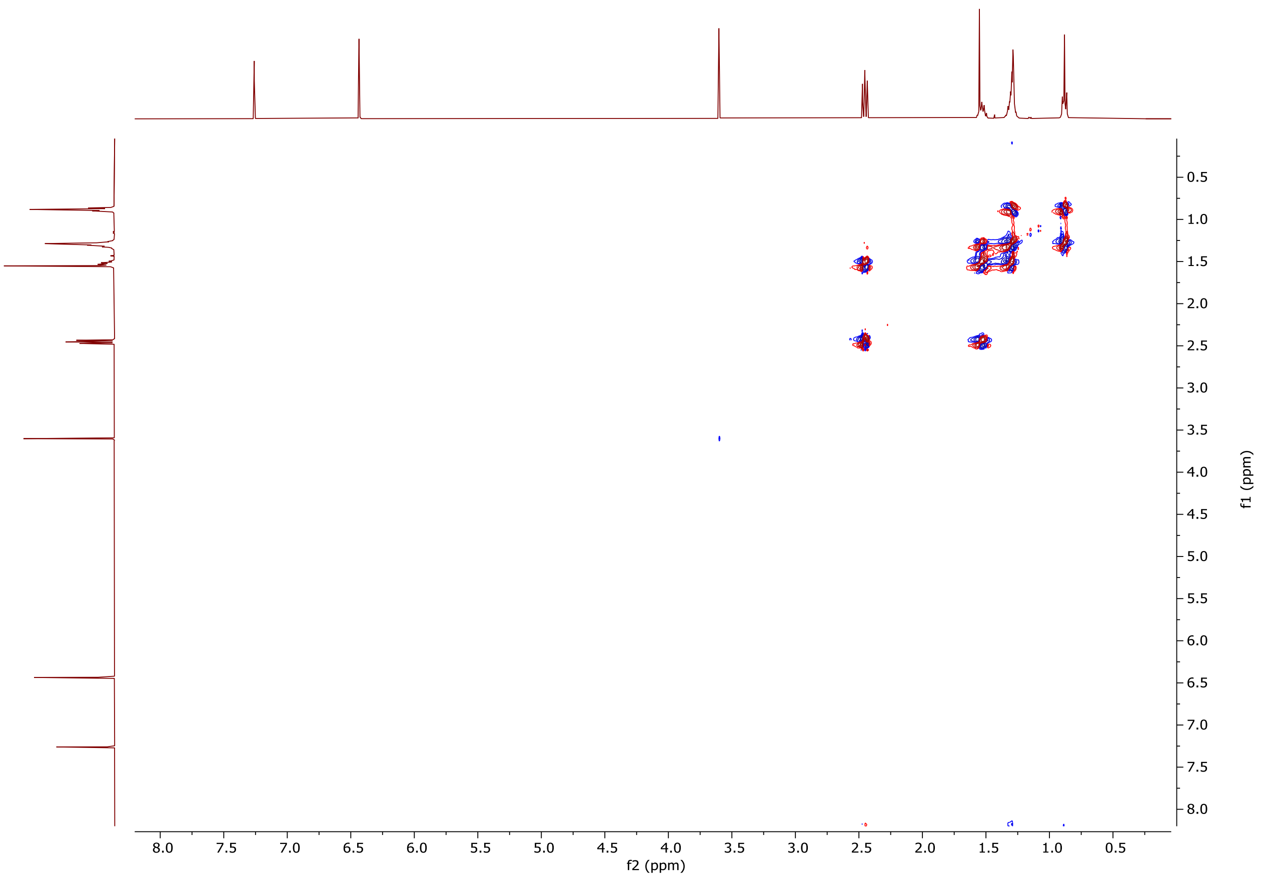

Supplementary Figure 14. COSY-NMR spectrum of **4** in $CDCl_3$ measured at 298 K.

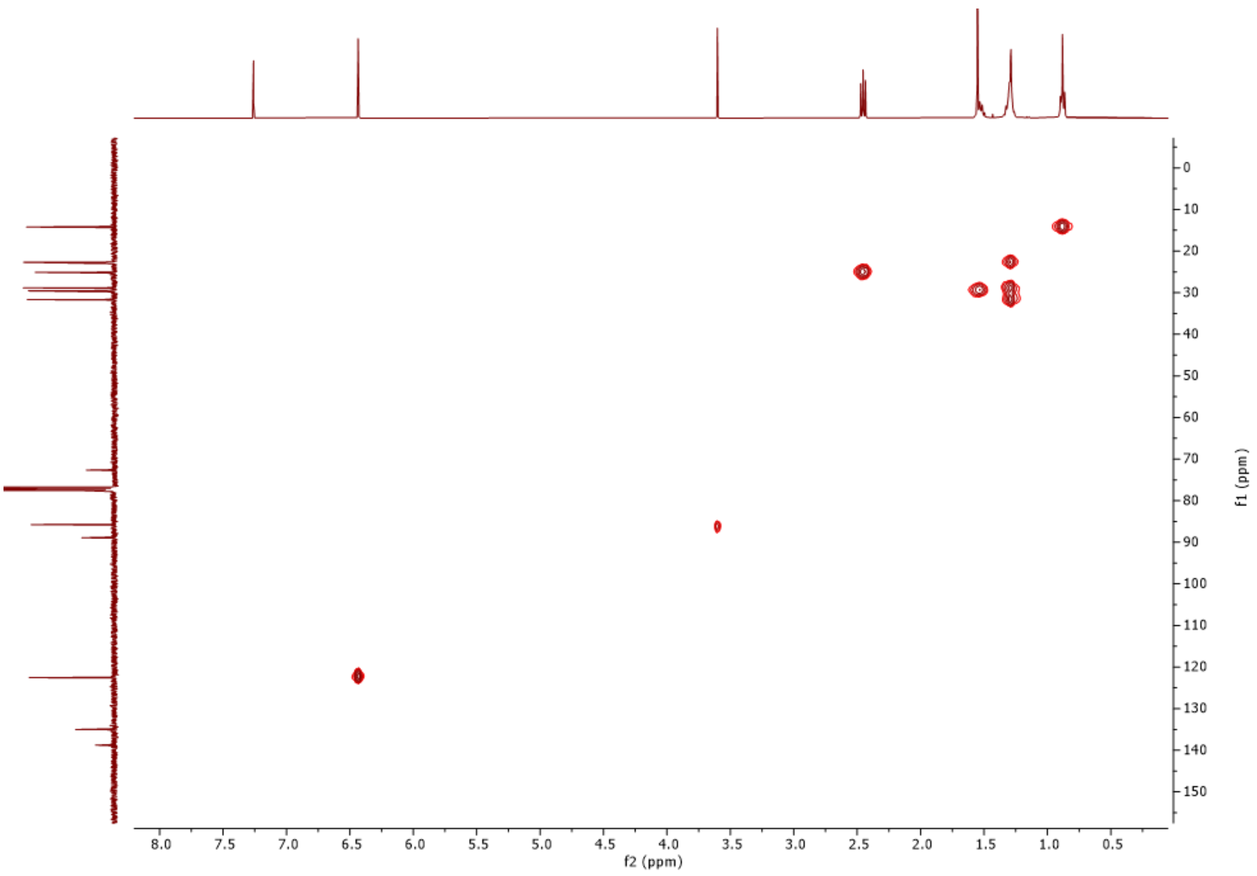

Supplementary Figure 15. HSQC-NMR spectrum of **4** in $CDCl_3$ measured at 298 K.

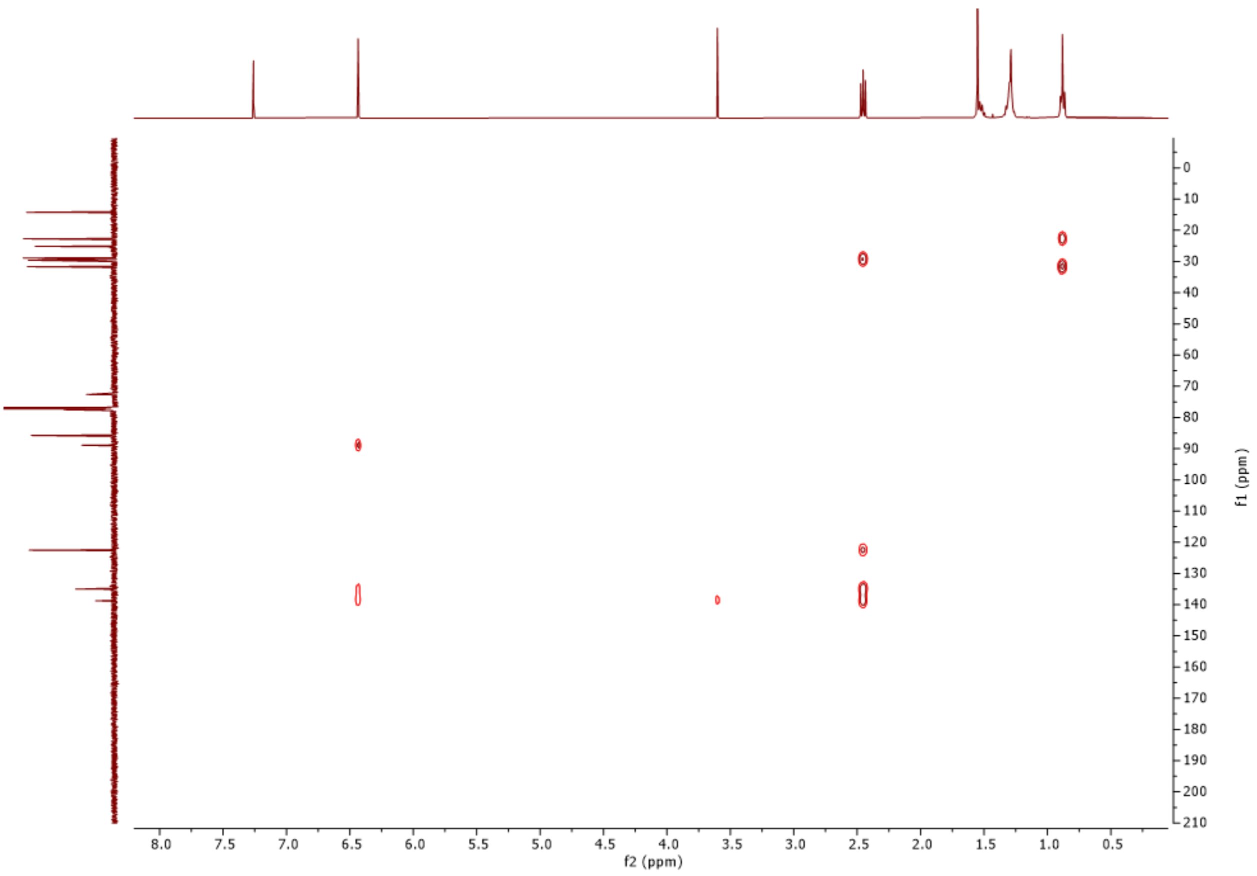

Supplementary Figure 16. HMBC-NMR spectrum of **4** in $CDCl_3$ measured at 298 K.

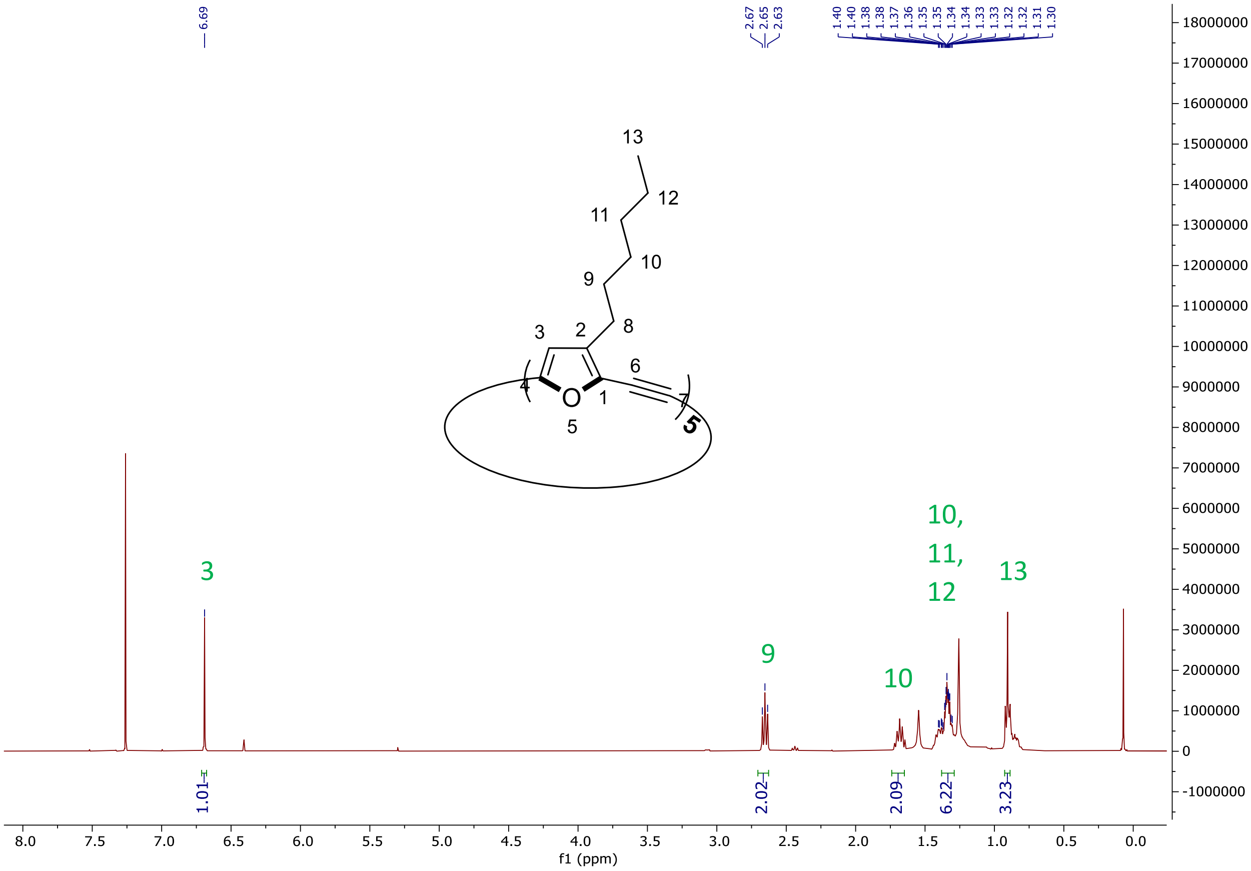

Supplementary Figure 17. ^{1}H-NMR spectrum of **C5** in $CDCl_3$ measured at 298 K.

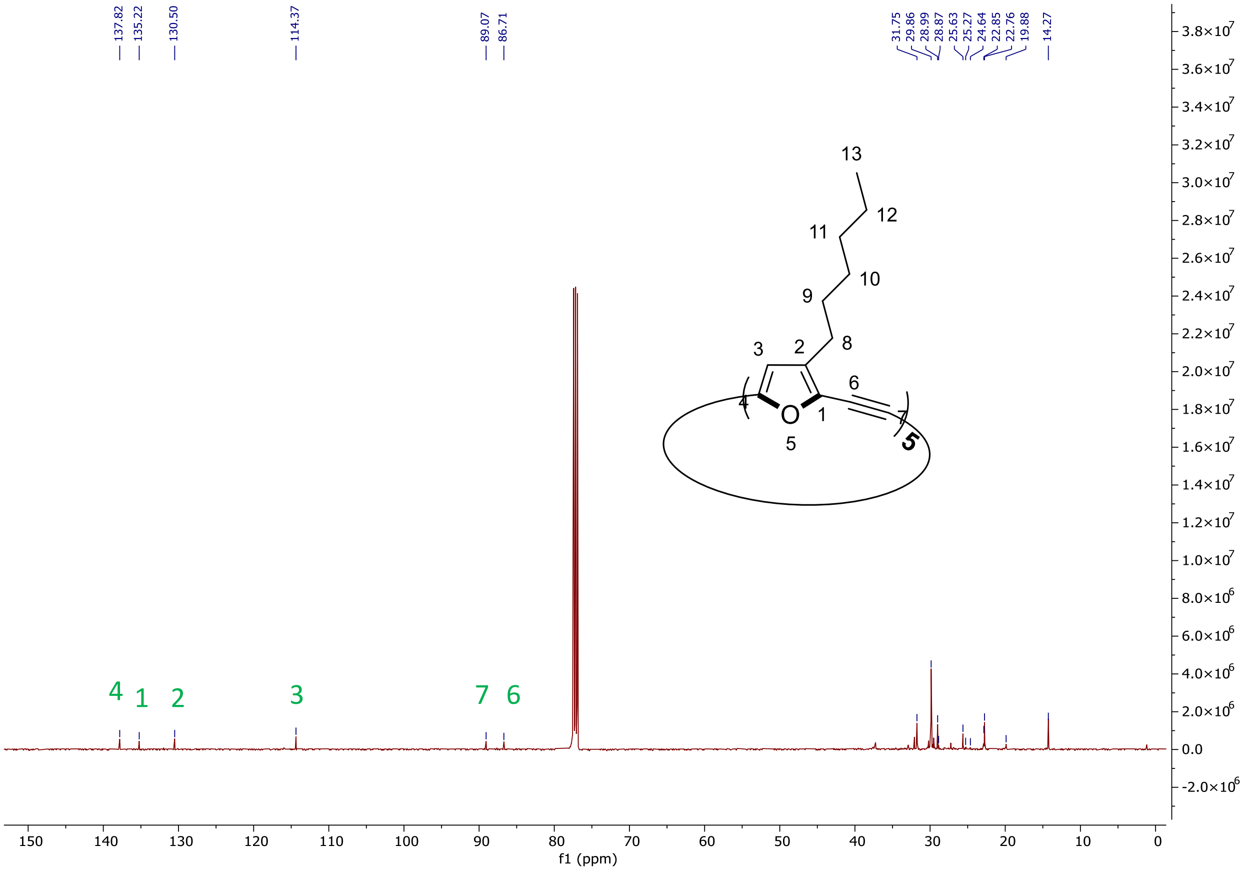

Supplementary Figure 18. ^{13}C-NMR spectrum of **C5** in $CDCl_3$ measured at 298 K.

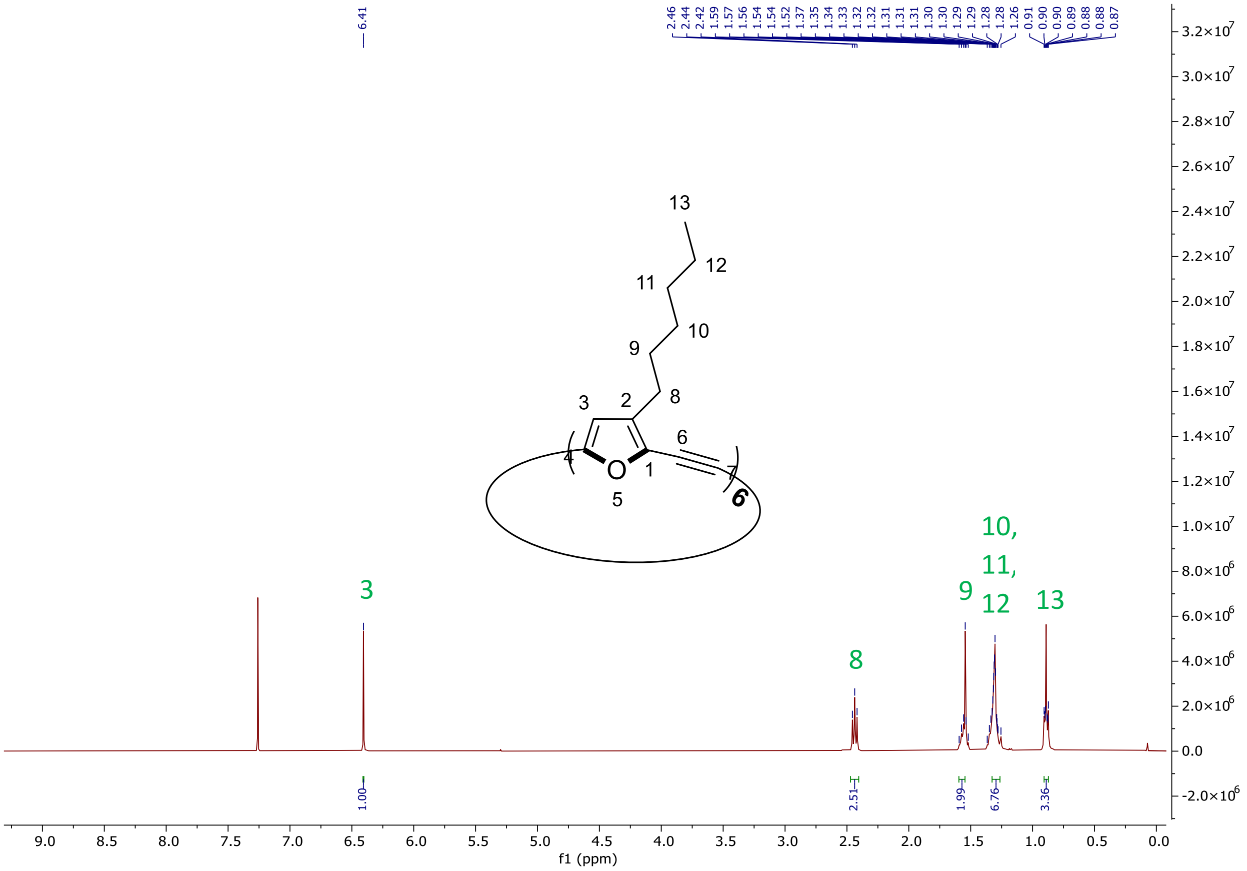

Supplementary Figure 19. ^{1}H-NMR spectrum of **C6** in $CDCl_3$ measured at 298 K.

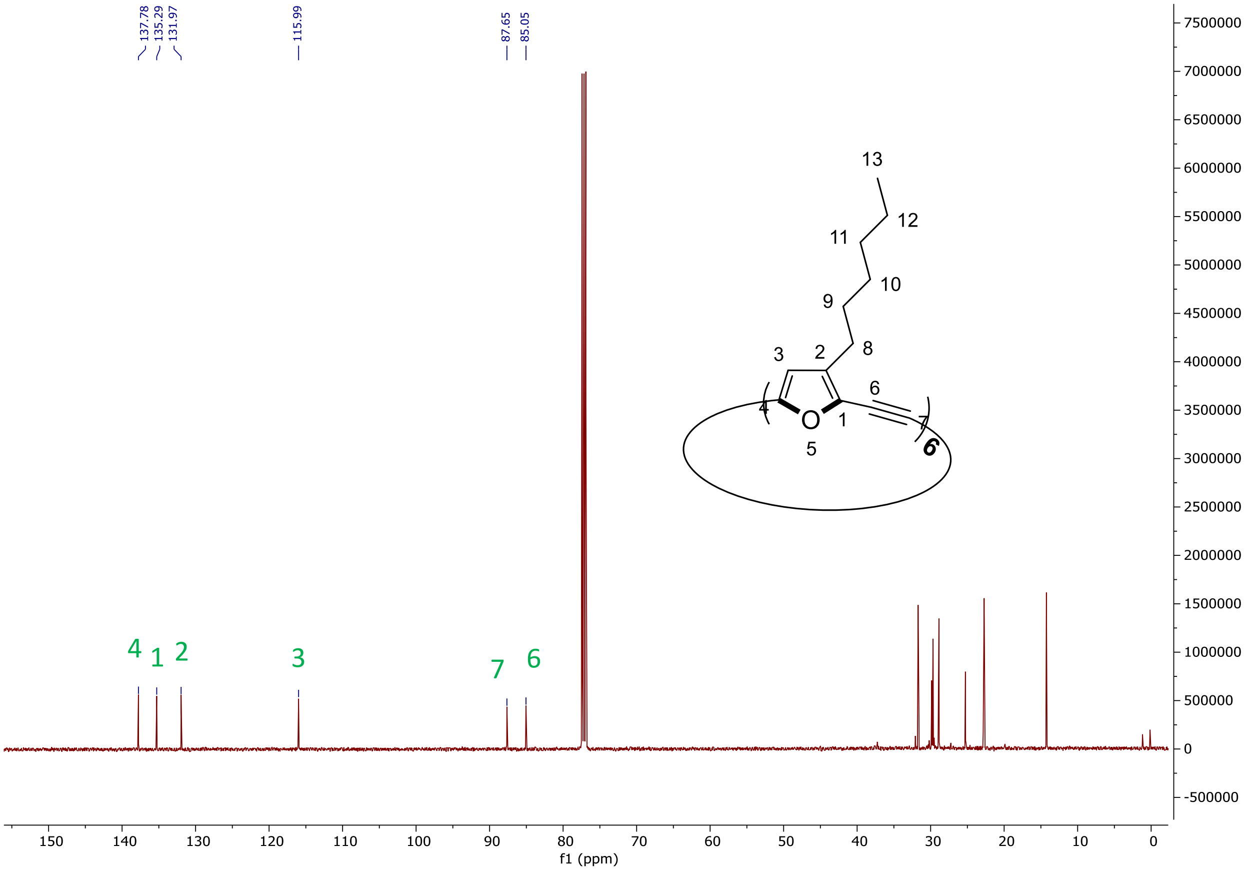

Supplementary Figure 20. ^{13}C-NMR spectrum of **C6** in $CDCl_3$ measured at 298 K.

1

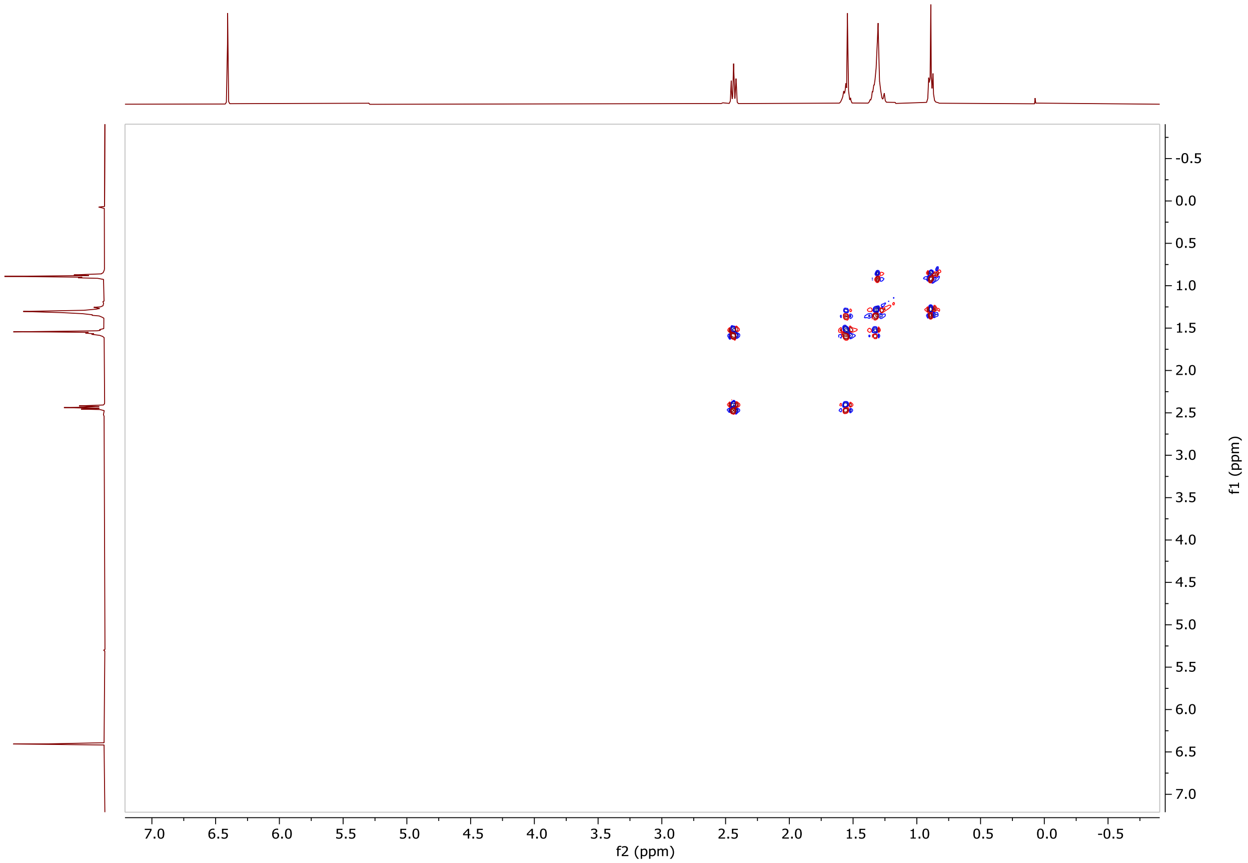

Supplementary Figure 21. COSY-NMR spectrum of **C6** in $CDCl_3$ measured at 298 K.

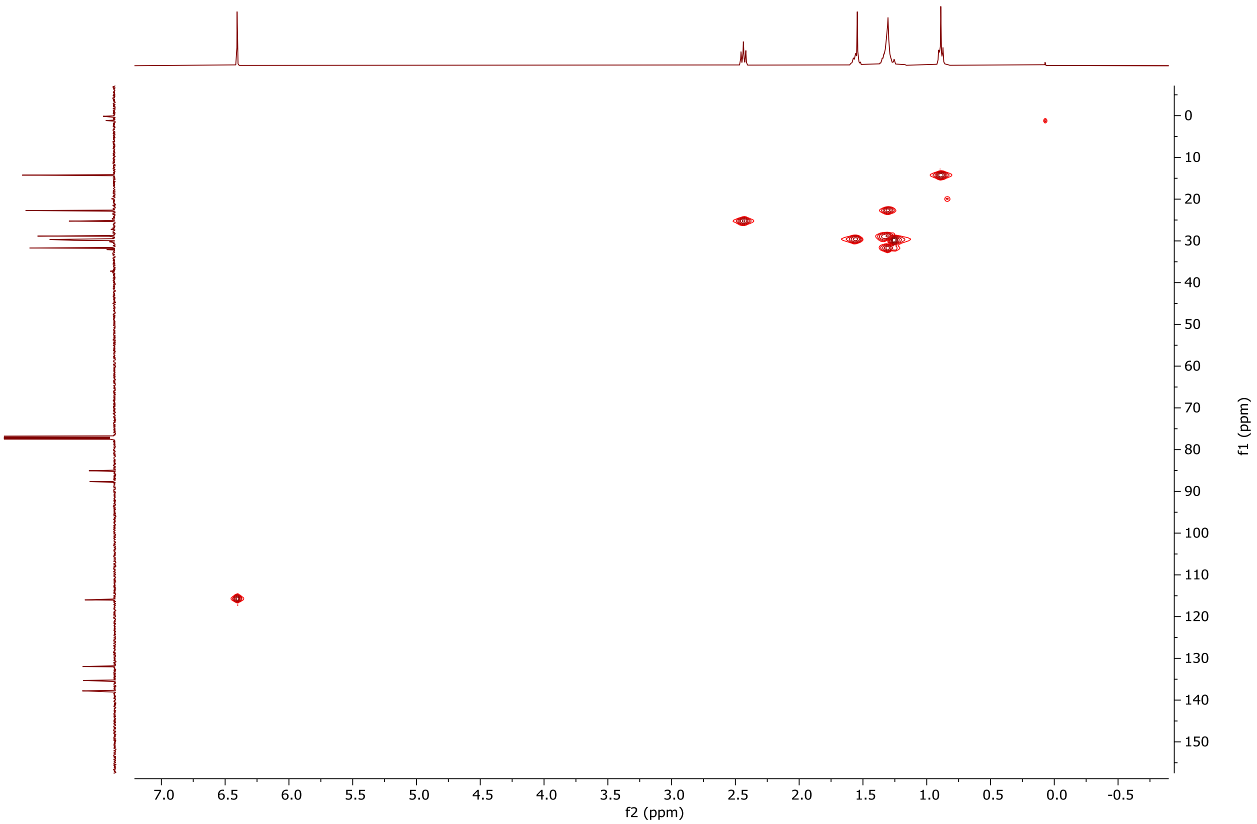

Supplementary Figure 22. HSQC-NMR spectrum of **C6** in $CDCl_3$ measured at 298 K.

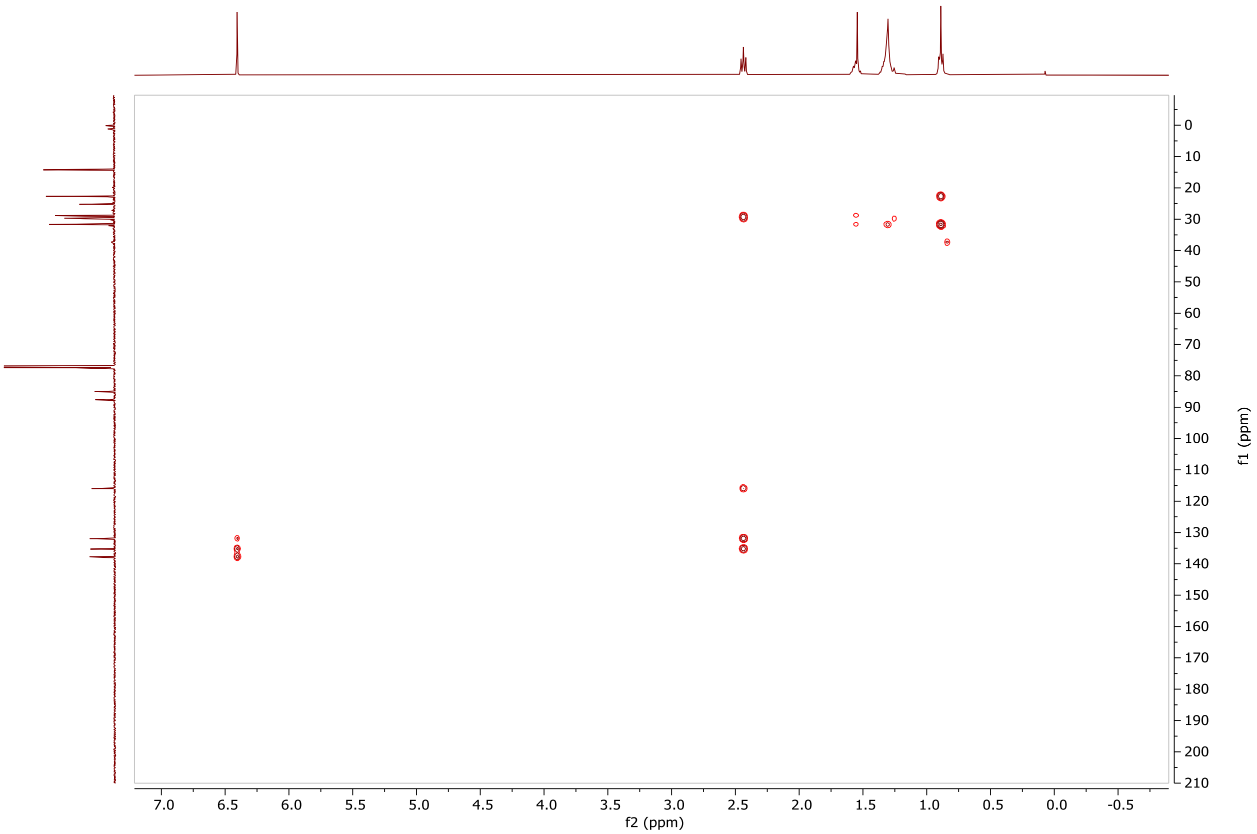

Supplementary Figure 23. HMBC-NMR spectrum of **C6** in $CDCl_3$ measured at 298 K.

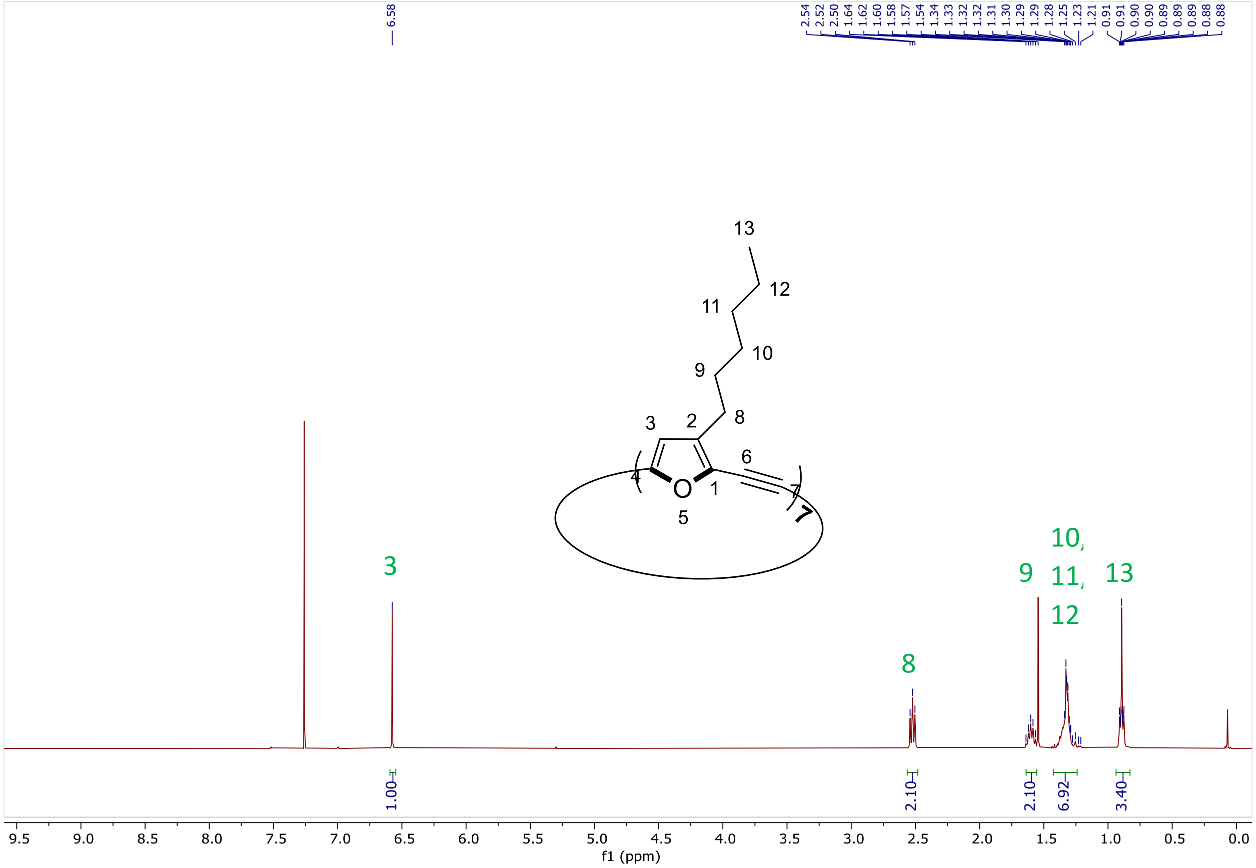

Supplementary Figure 24. ^{1}H-NMR spectrum of **C7** in $CDCl_3$ measured at 298 K.

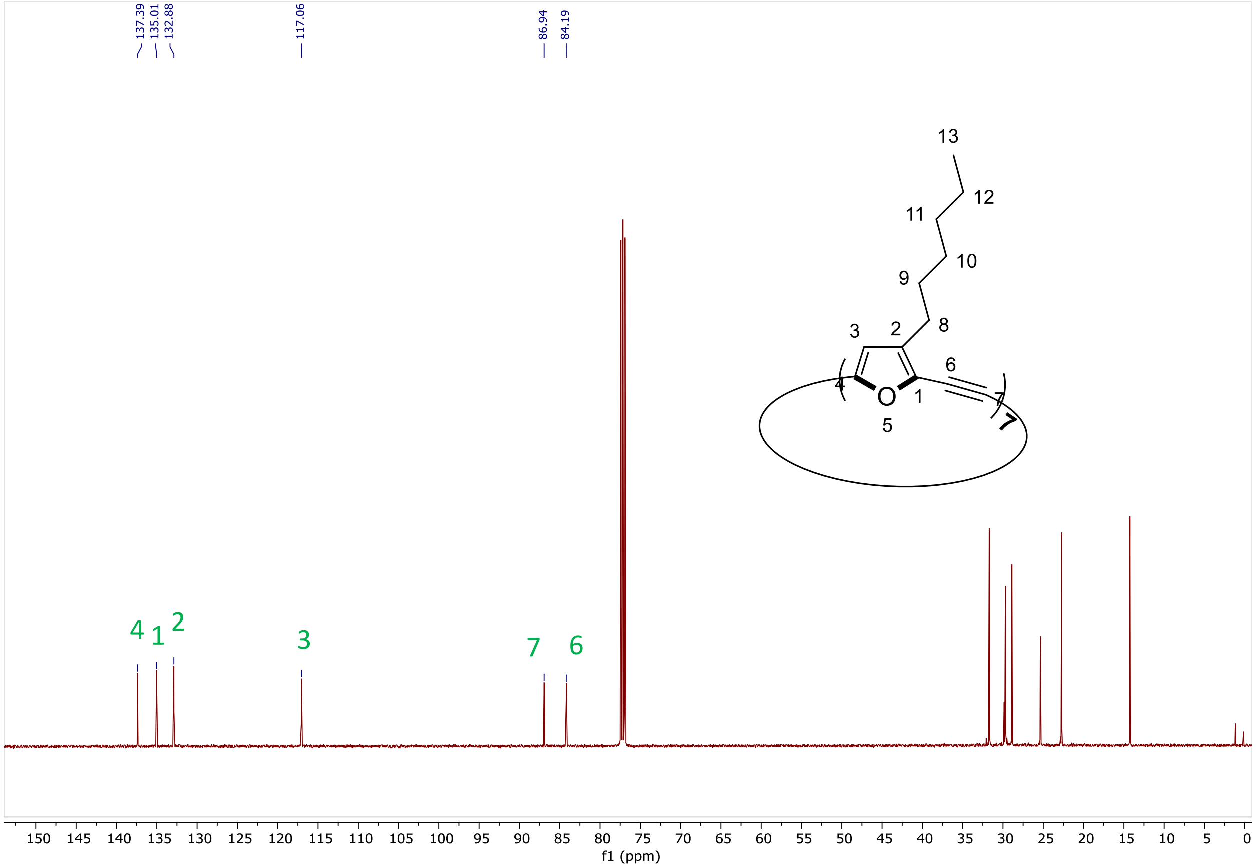

Supplementary Figure 25. ^{13}C-NMR spectrum of **C7** in $CDCl_3$ measured at 298 K.

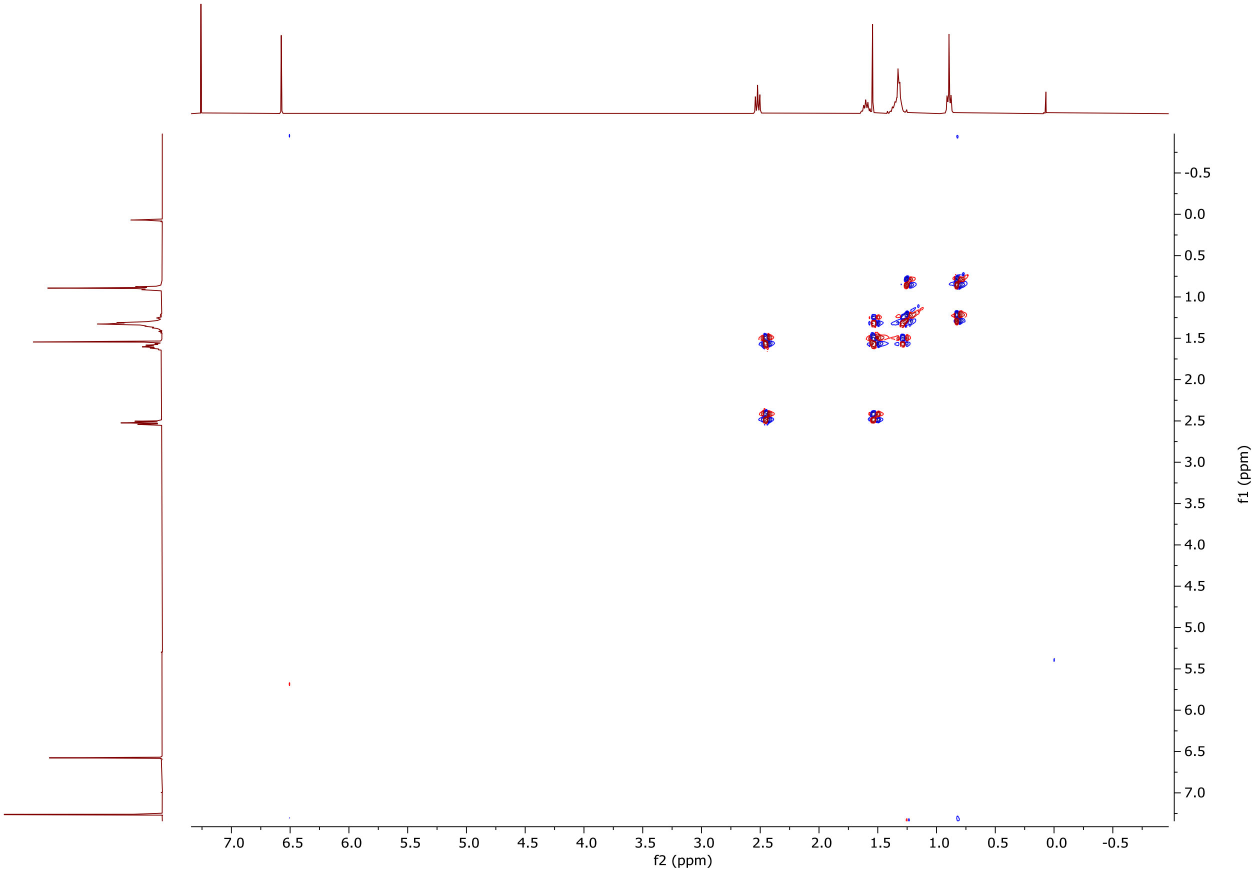

Supplementary Figure 26. COSY-NMR spectrum of **C7** in $CDCl_3$ measured at 298 K.

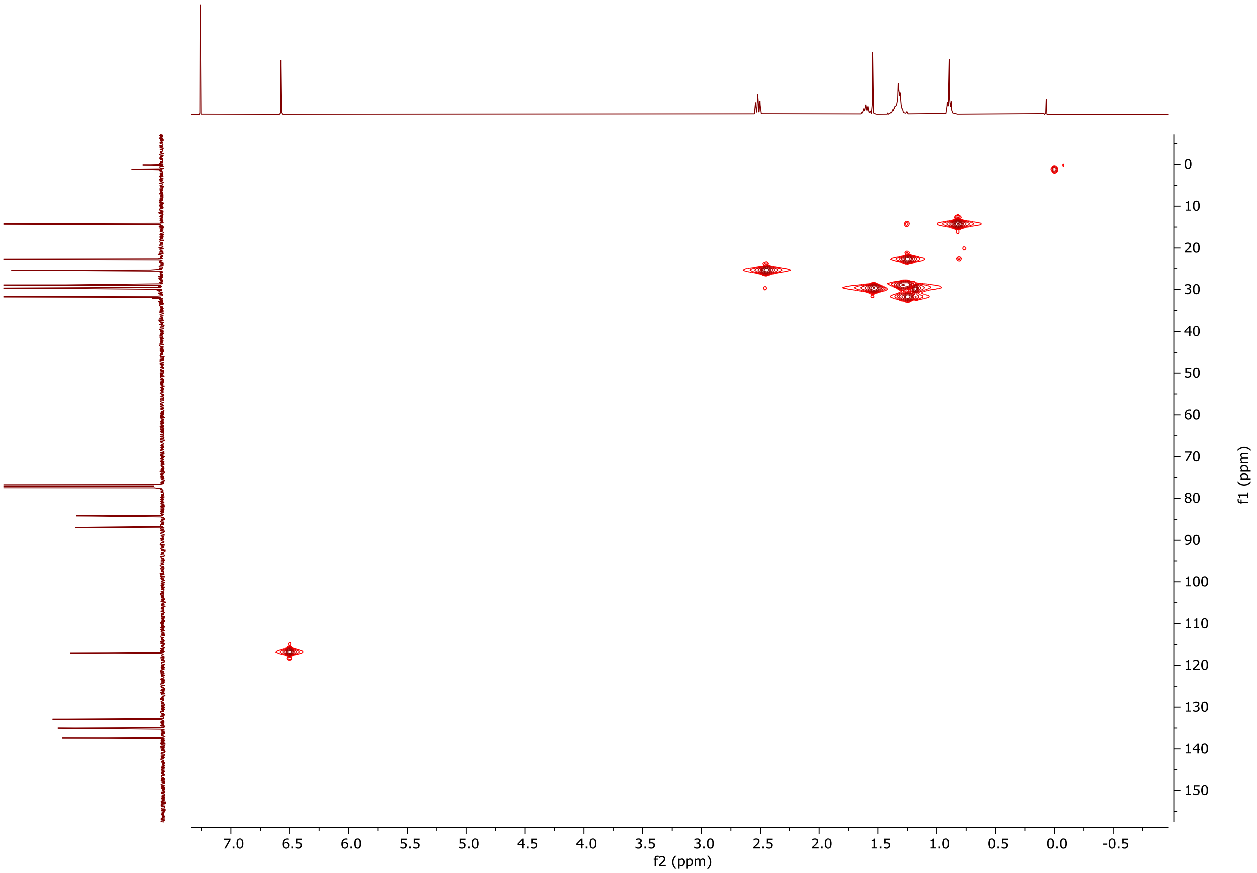

Supplementary Figure 27. HSQC-NMR spectrum of **C7** in $CDCl_3$ measured at 298 K.

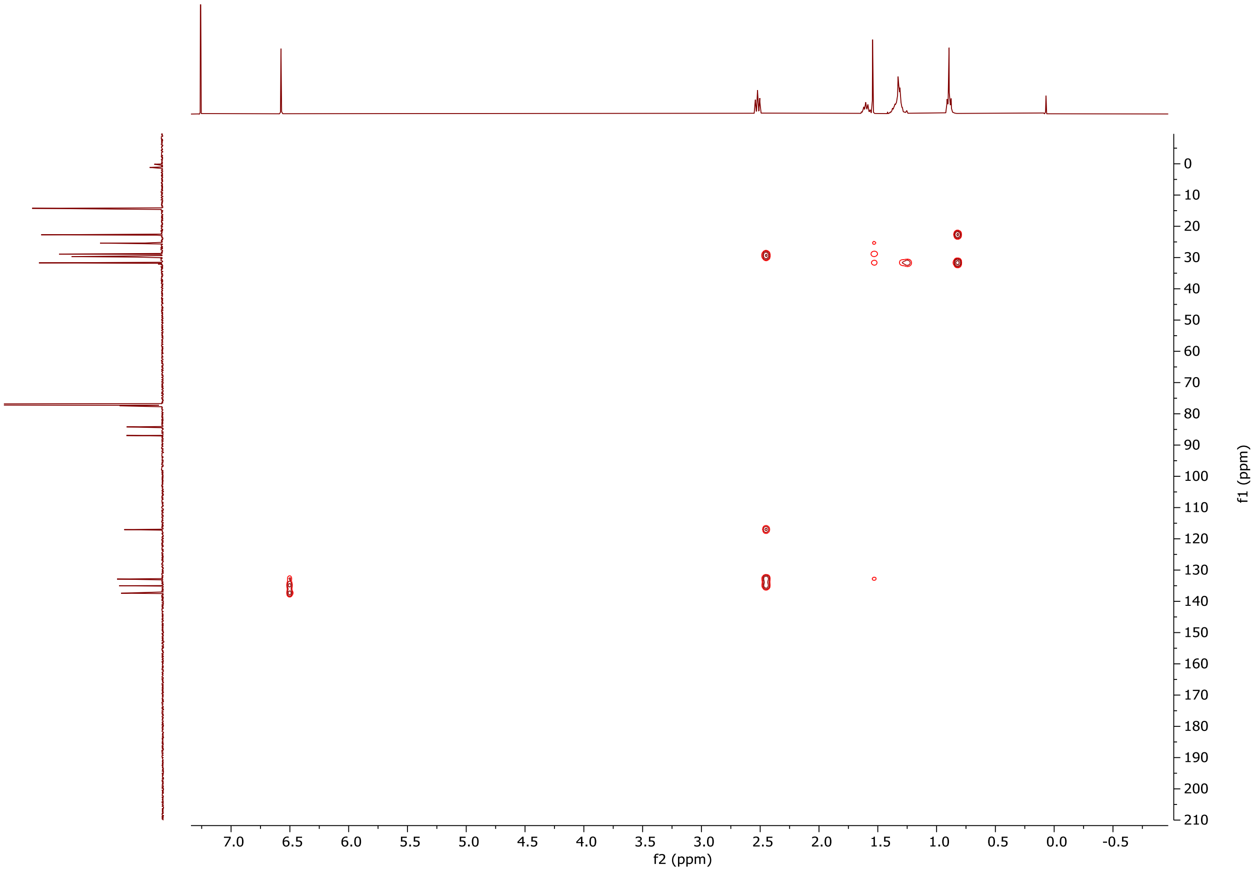

Supplementary Figure 28. HMBC-NMR spectrum of **C7** in $CDCl_3$ measured at 298 K.

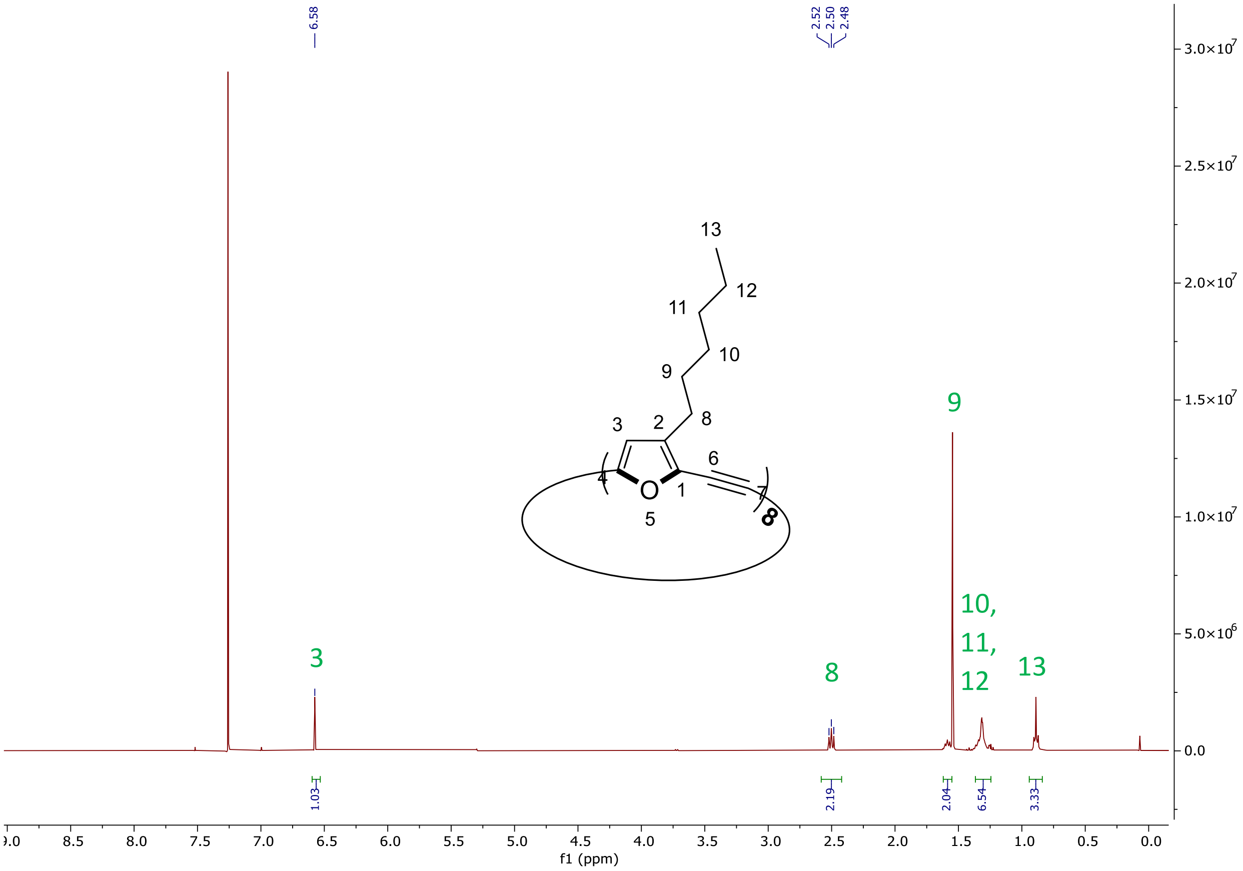

Supplementary Figure 29. ^{1}H-NMR spectrum of **C8** in $CDCl_3$ measured at 298 K.

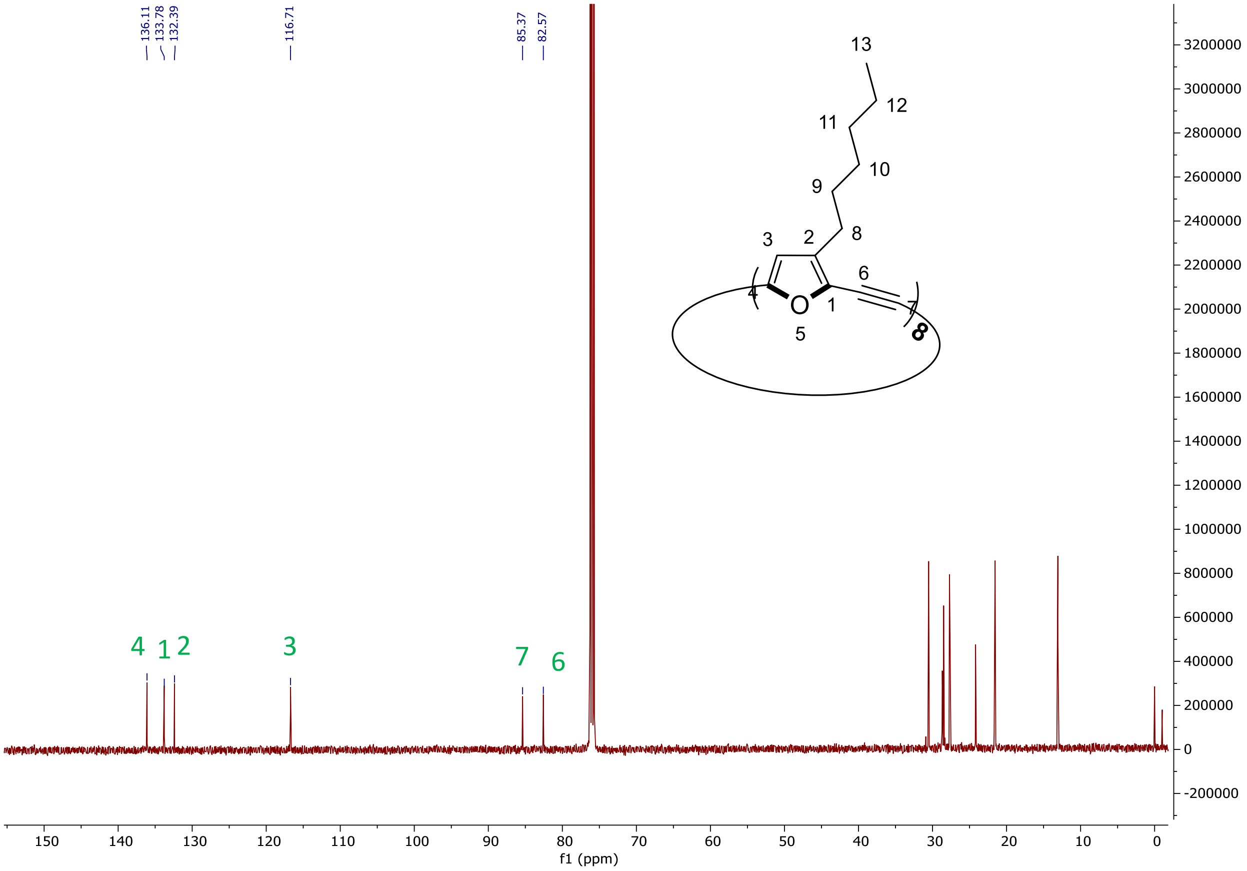

Supplementary Figure30. ^{13}C-NMR spectrum of **C8** in $CDCl_3$ measured at 298 K.

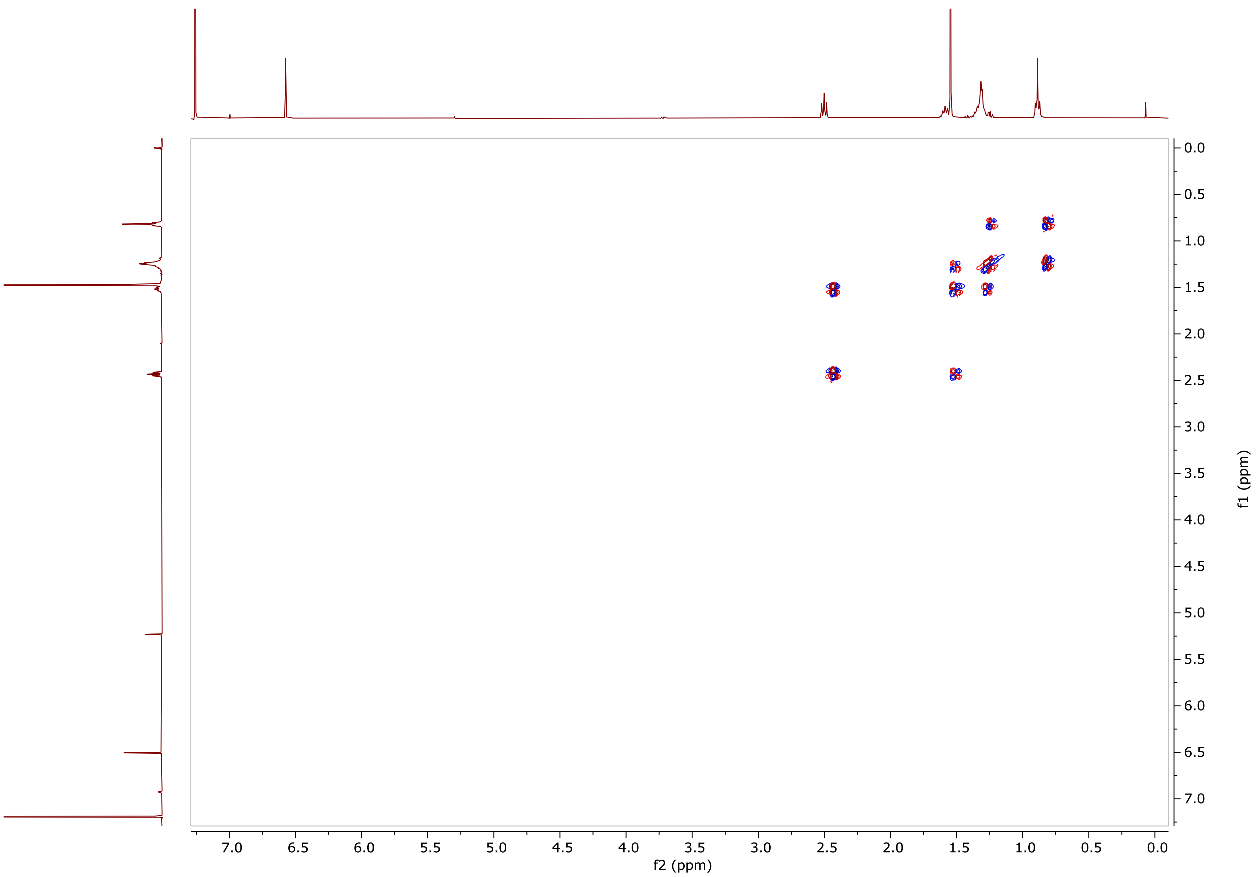

Supplementary Figure 31. COSY spectrum of **C8** in $CDCl_3$ measured at 298 K.

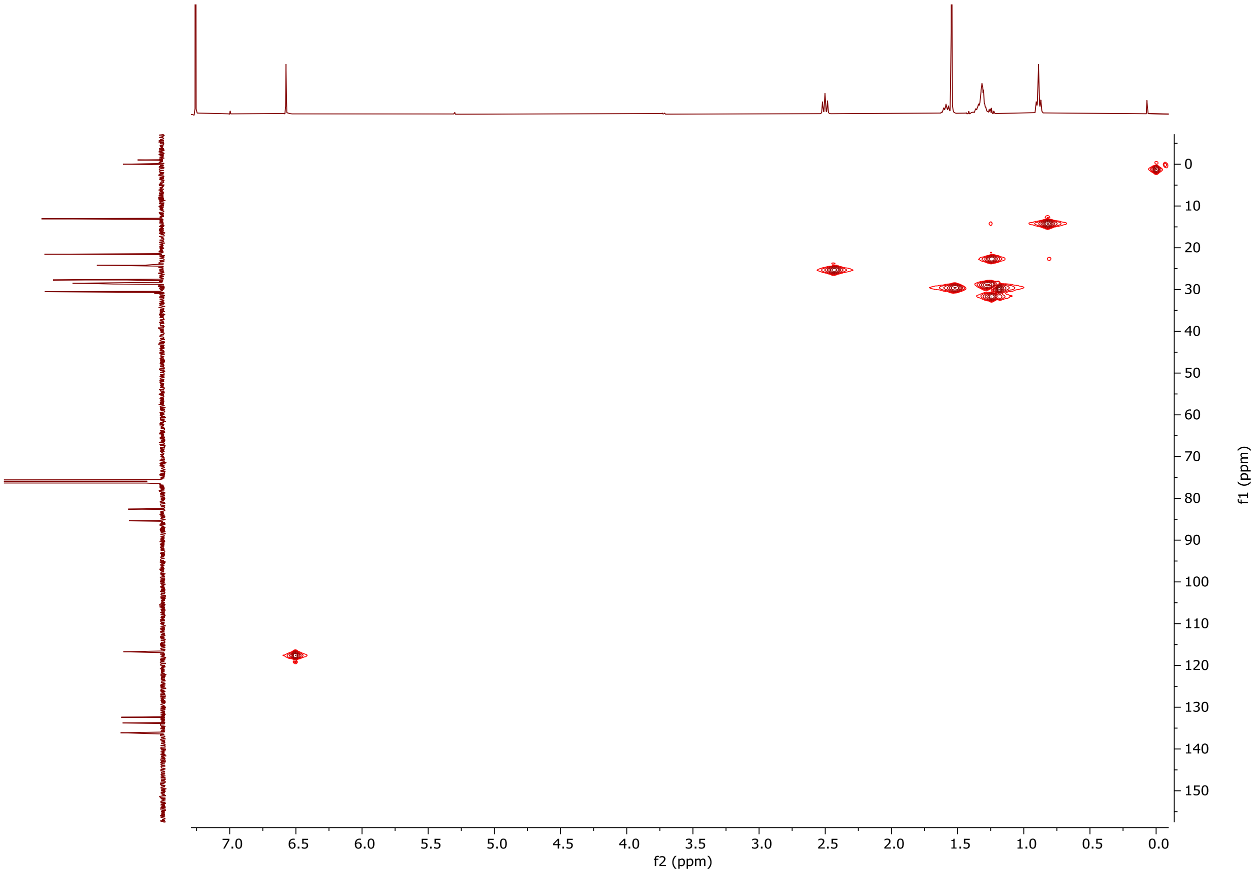

Supplementary Figure 32. HSQC spectrum of **C8** in $CDCl_3$ measured at 298 K.

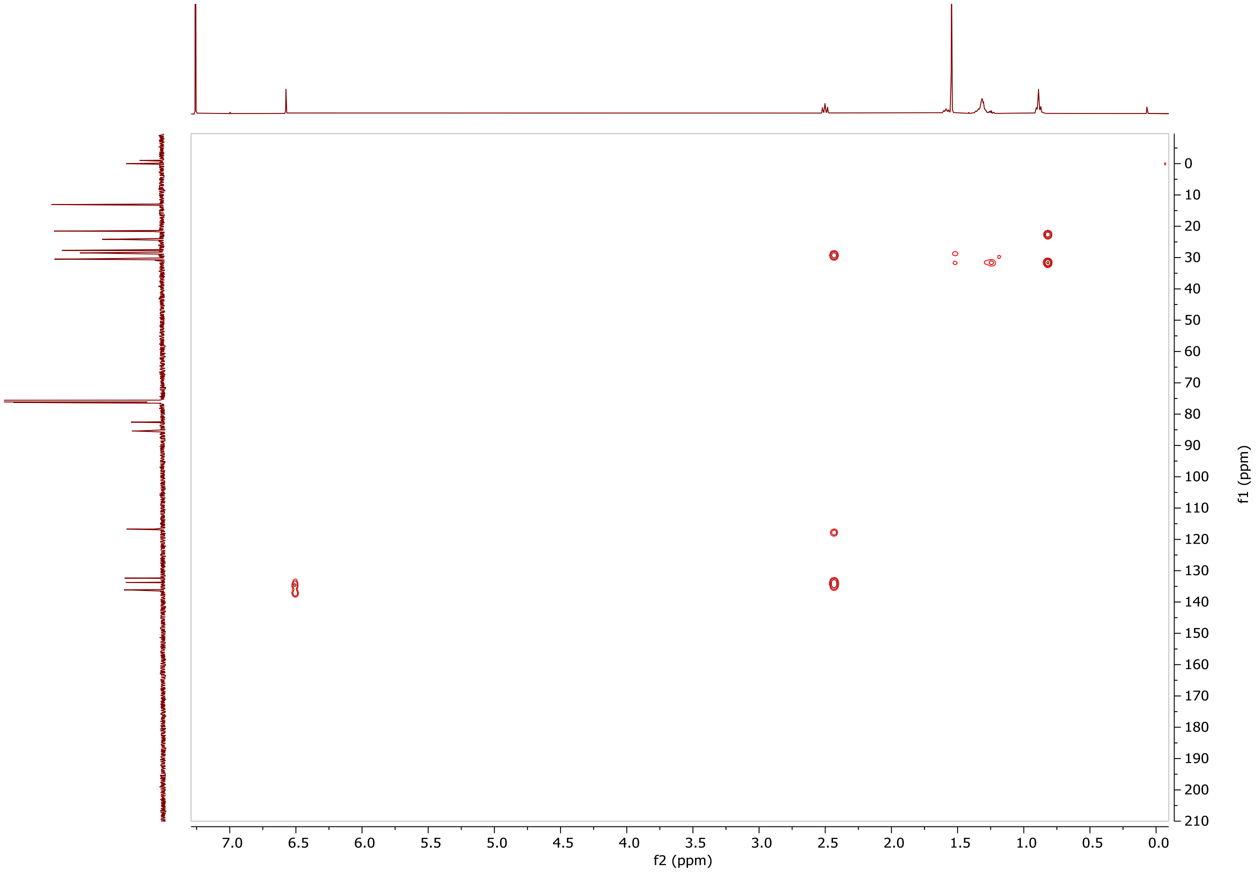

Supplementary Figure 33. HMBC spectrum of **C8** in $CDCl_3$ measured at 298 K.

S3.2 MALDI-TOF

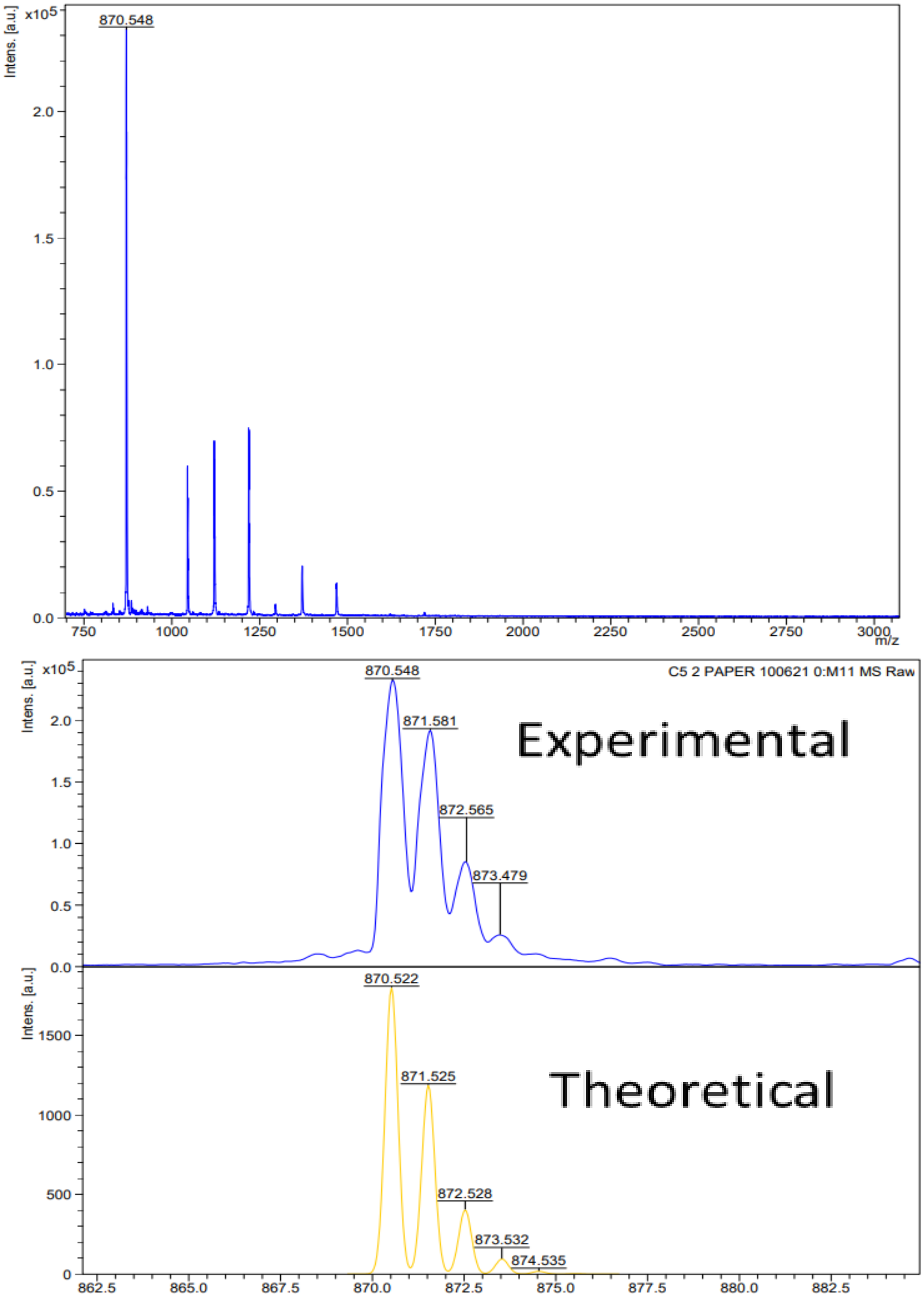

Supplementary Figure 34. MALDI-TOF spectrum of **C5**. (Top) full spectra and (bottom) excerpt of target region.

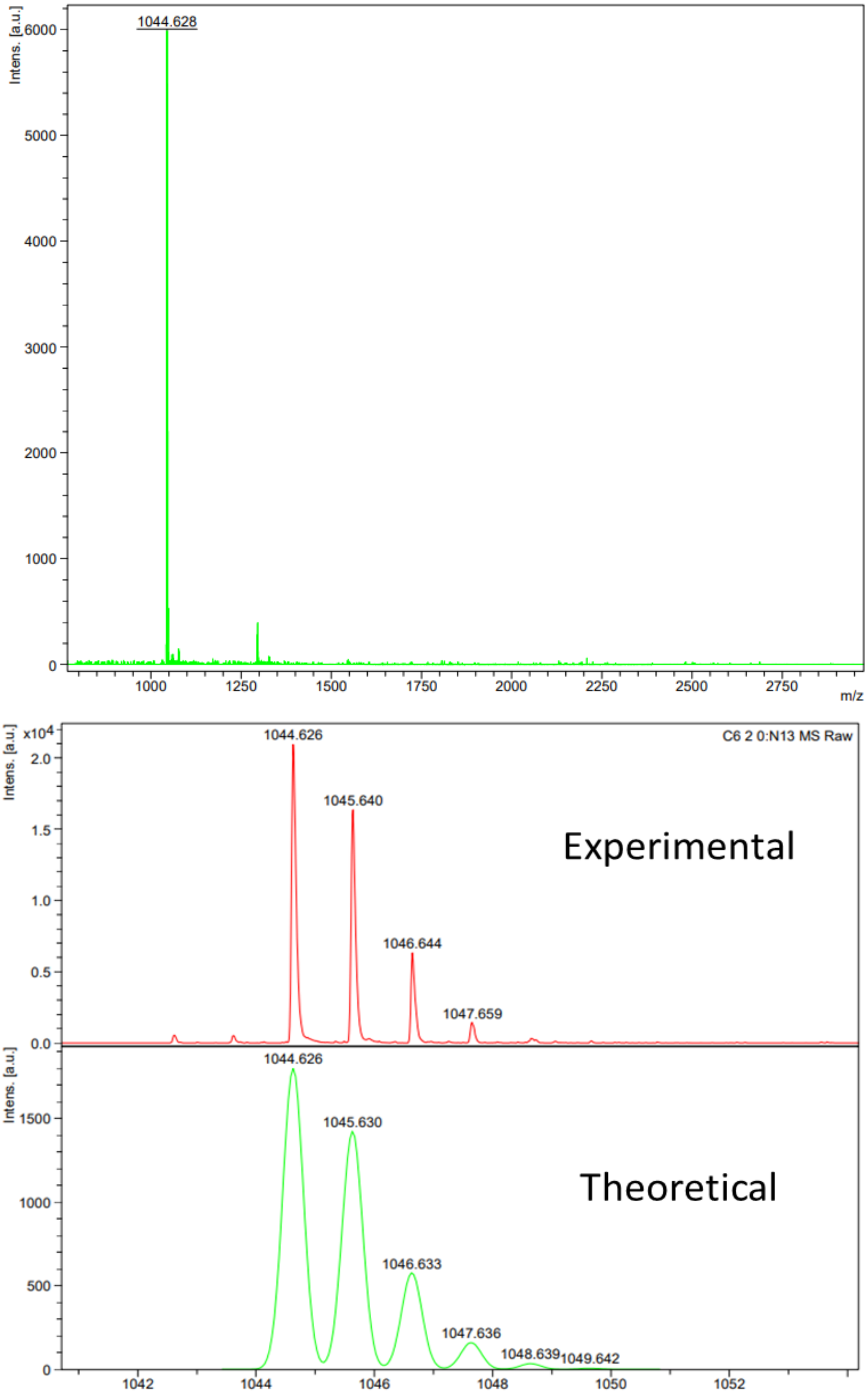

Supplementary Figure 35. MALDI-TOF spectrum of **C6**. (Top) full spectra and (bottom) excerpt of target region.

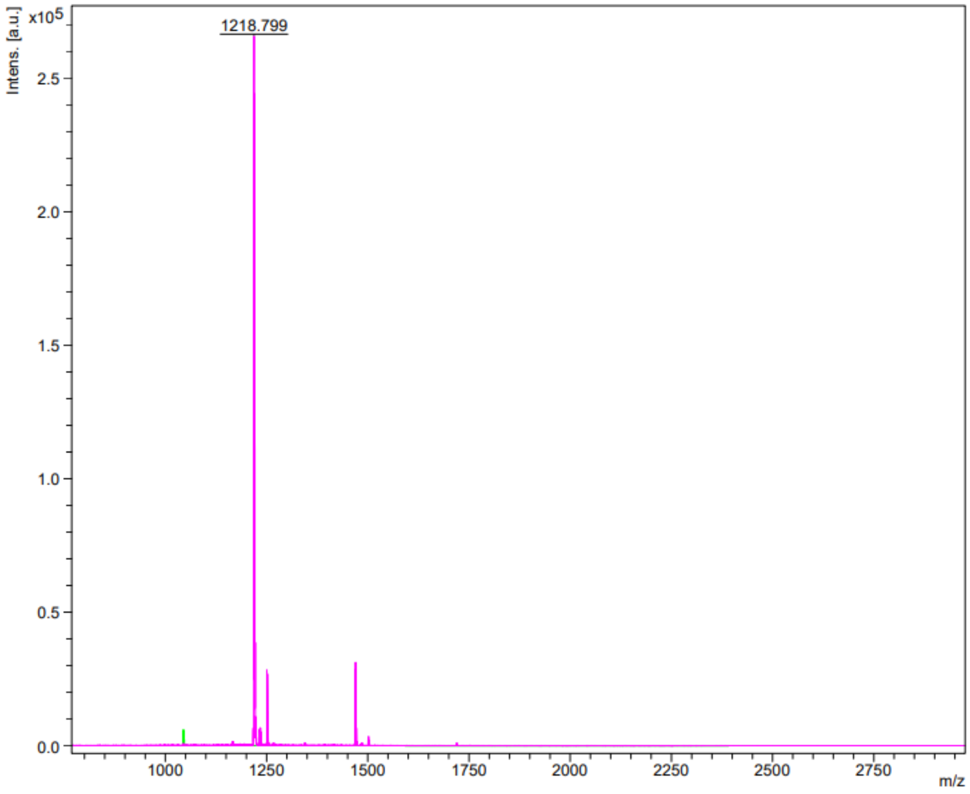

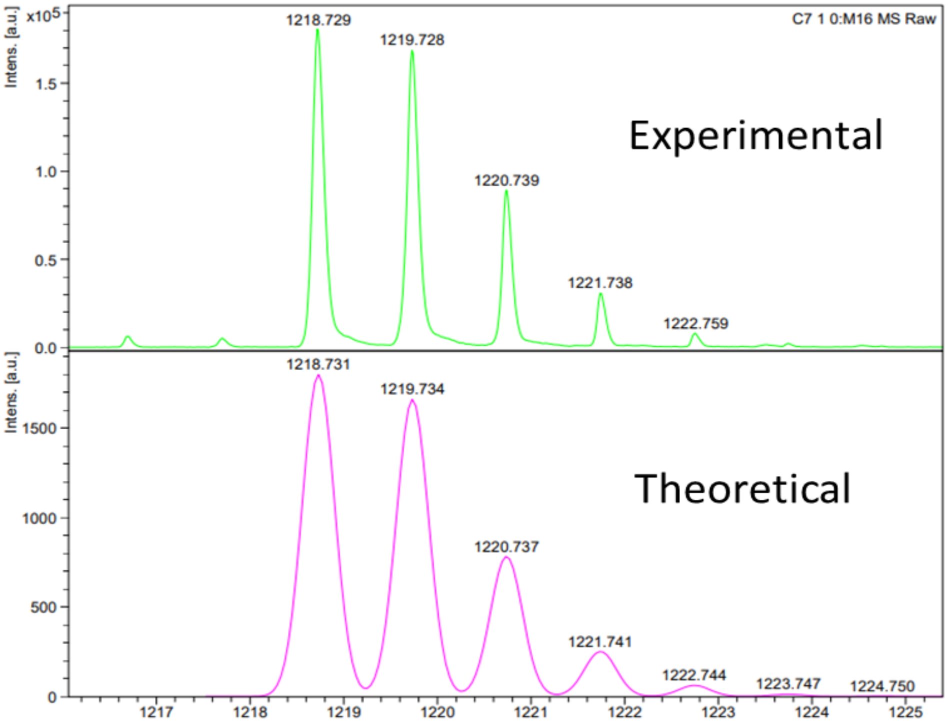

Supplementary Figure 36. MALDI-TOF spectrum of **C7**. (Top) full spectra and (bottom) excerpt of target region.

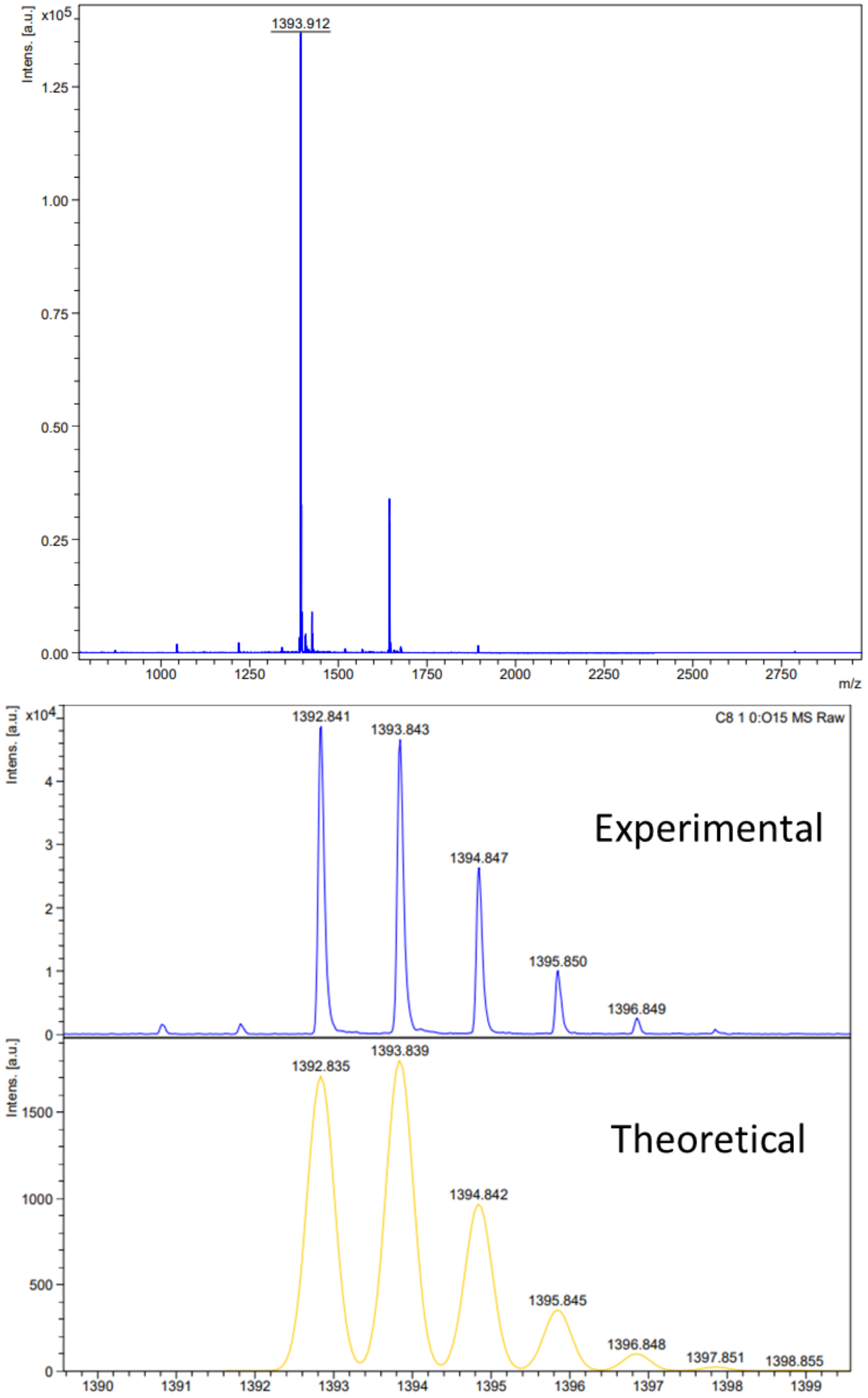

Supplementary Figure 37. MALDI-TOF spectrum of **C8**. (Top) full spectra and (bottom) excerpt of target region

S3.3. Experimental Absorption, Emission, and Excitation Spectra

a) b)

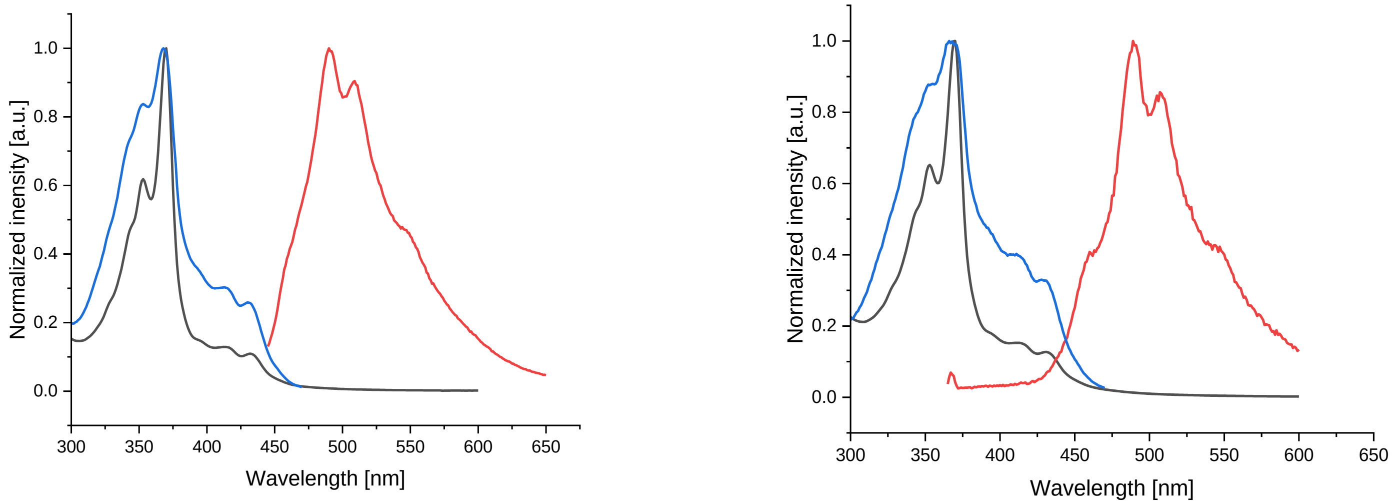

Supplementary Figure 38. Normalized absorption (black), emission (red, excited at 369 nm) and excitation (blue, emission at 490 nm) spectra of **C5** in (a) $CHCl_3$ and (b) toluene at 298K.

a) b)

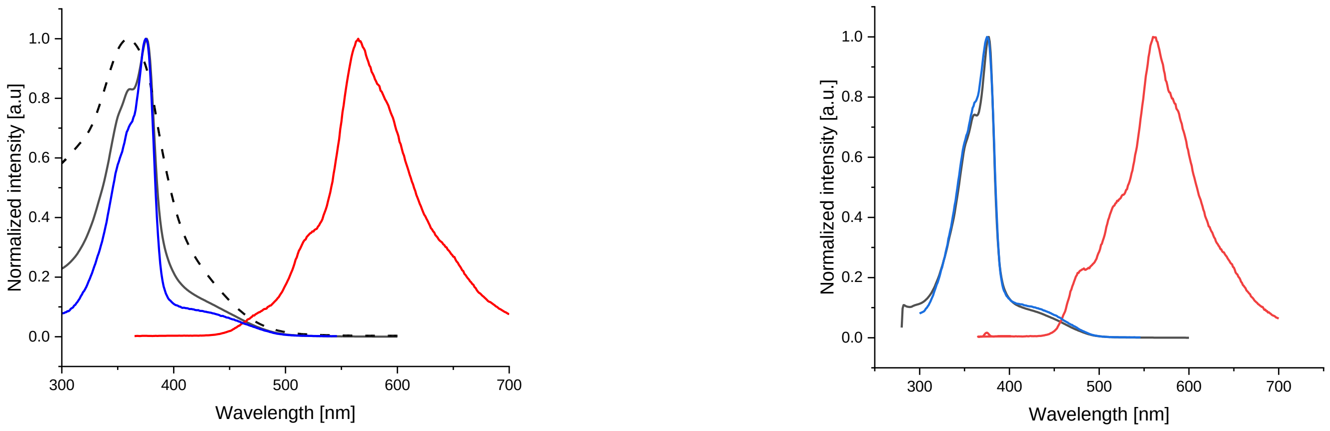

Supplementary Figure 39. Normalized absorption (black 0.4 mM, dash black 0.01 mM), emission (red, excited at 360 nm and 376nm, and excitation (blue, emission at 566 nm) spectra of **C6** in (a) $CHCl_3$ and (b) toluene at 298K.

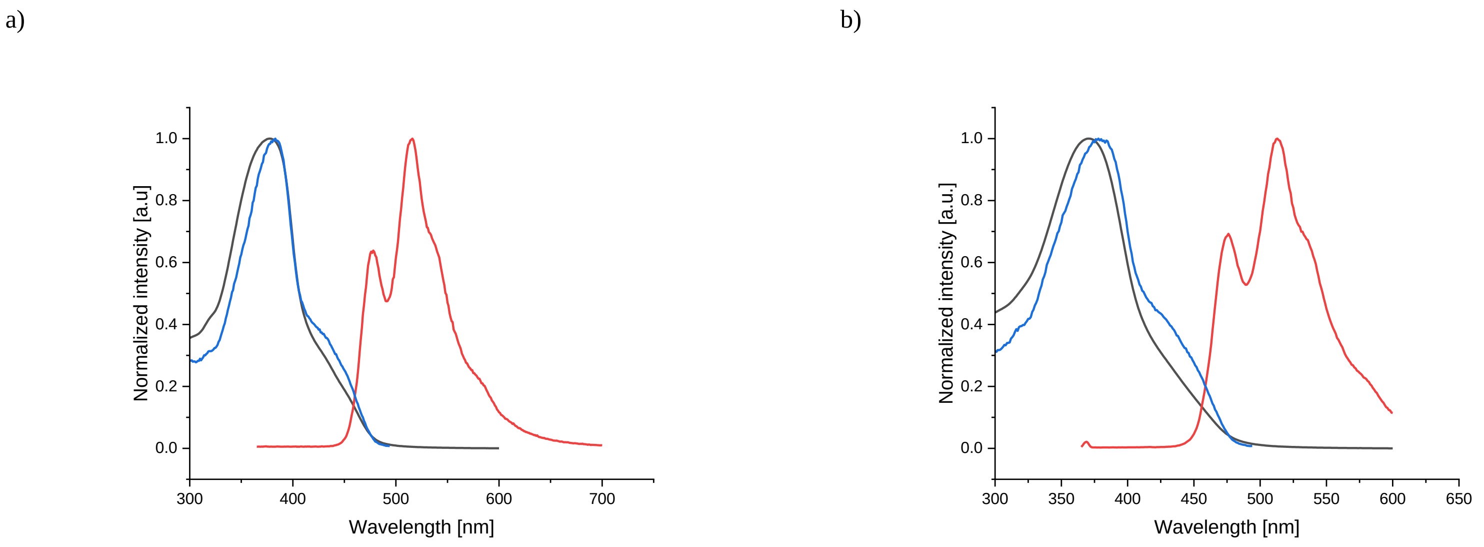

Supplementary Figure 40. Normalized absorption (black), emission (red, excited at 380 nm in $CHCl_3$ and at 370 nm in toluene) and excitation (blue, emission at 514 nm) spectra of **C7** in (a) $CHCl_3$ and (b) toluene at 298K.

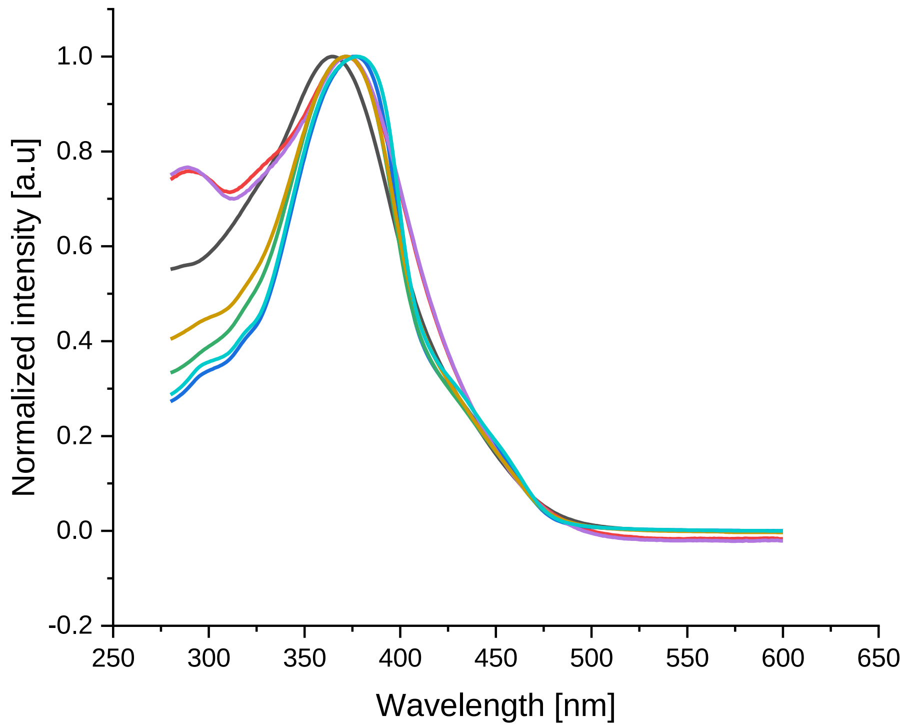

Supplementary Figure 41. Normalized absorption of **C7** in different concentrations in $CHCl_3$ at 298K.

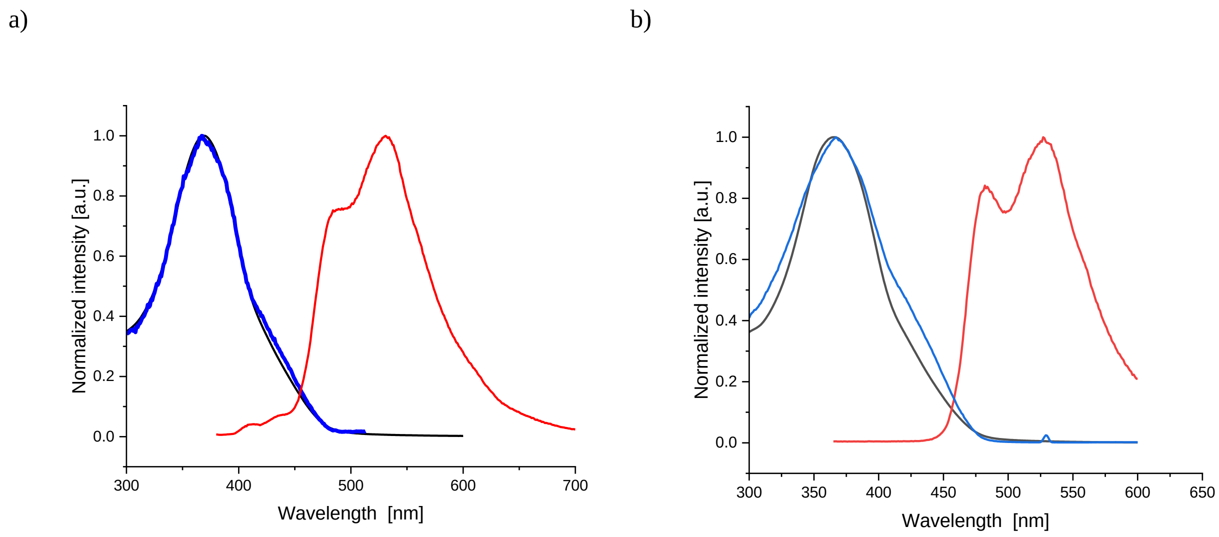

Supplementary Figure 42. Normalized absorption (black), emission (red, excited at 370 nm in $CHCl_3$ and at 365 nm in toluene) and excitation (blue, emission at 533 nm in $CHCl_3$ and at 528 nm in toluene) spectra of **C8** in (a) $CHCl_3$ and (b) toluene at 298K.

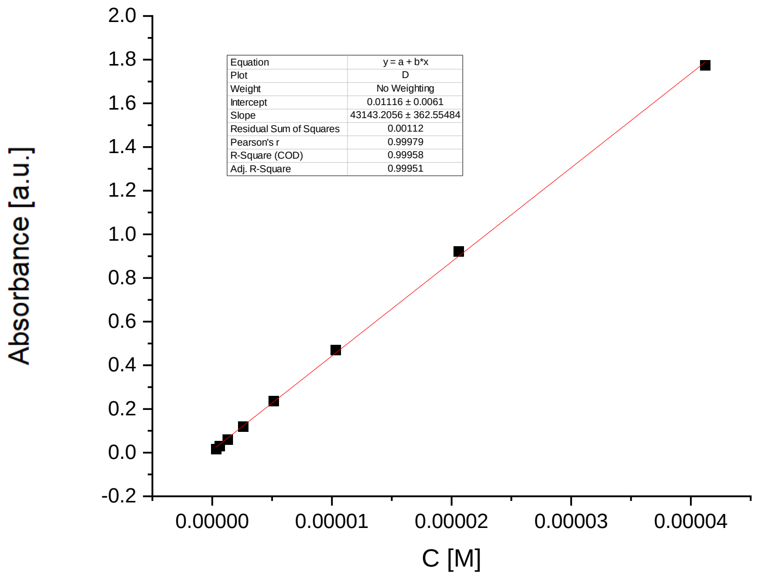

Supplementary Figure 43. Linear fit used to calculate the molar absorption coefficient of **C8** at 365 nm (λ_{max}) in toluene.

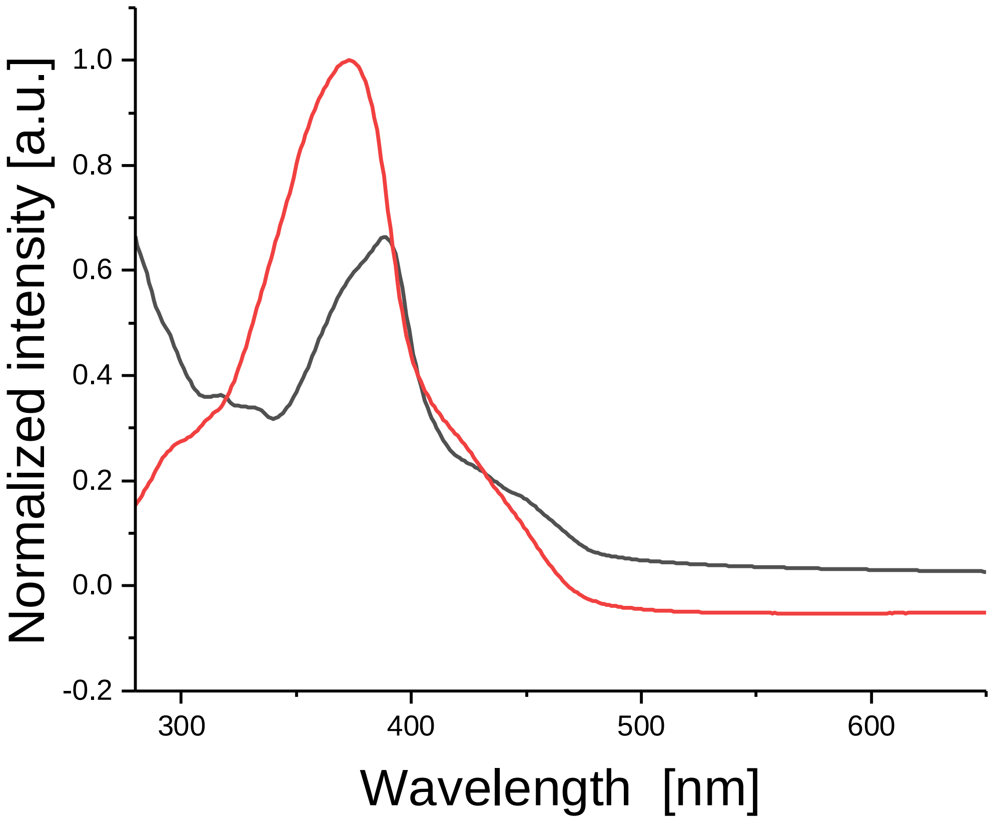

Supplementary Figure 44. UV-Vis spectrum of **C7** in EPA (ethanol:isopentane:diethylether 2:5:5) at 300K (black) and 130K (red).

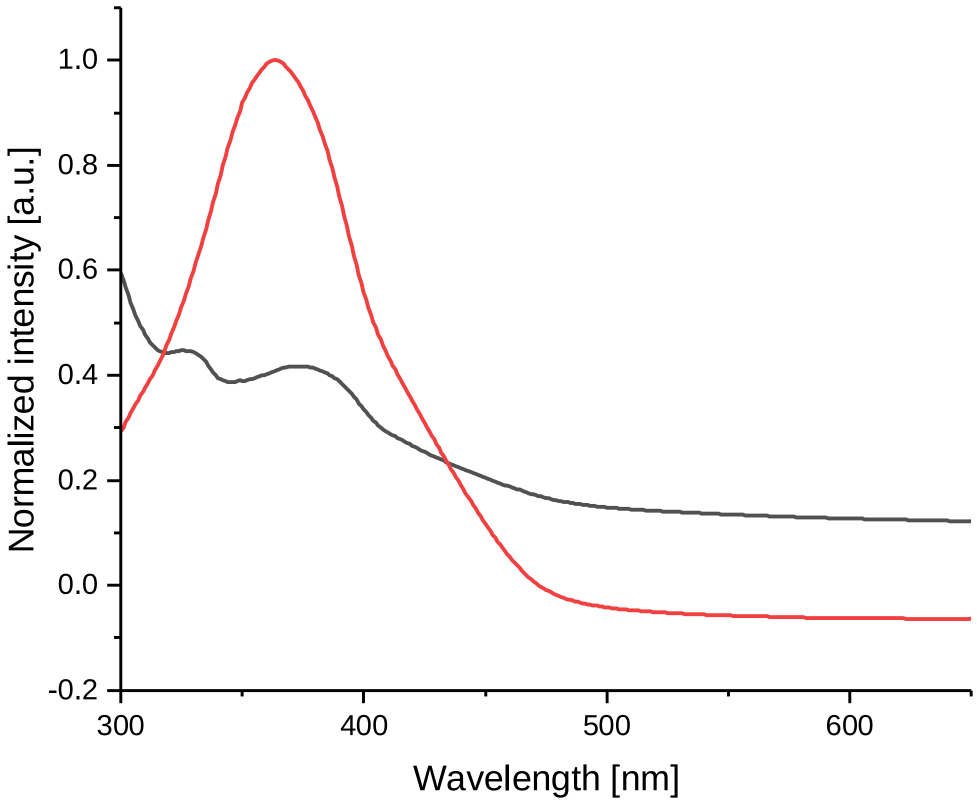

Supplementary Figure 45. UV-Vis spectrum of **C8** in EPA (ethanol:isopentane:diethylether 2:5:5) at 300K (black) and 130K (red).

Supplementary Table2. Summary of photophysical properties of **Cm**. (a) values measured in $CHCl_3$ and (b) values measured in toluene at 298K.

Molecule	λ_{max} *abs* (*nm*)	λ_{max} *em* (*nm*)	ϵ	$\emptyset_{solution}$ (%)^{a}	τ_f (*ns*)^{a}
**C5**	369 ^{a,b}	490 ^{a,b}	-	6	2.5
**C6**	360 ^{a,b}, 376 ^{a,b}	566^{a}	443443.52^{b}, 64000.31^{b}	7	3.5
**C7**	380^{a}, 370^{b}	514 ^{a,b}	43143.20^{b}	35	4
**C8**	370 ^{a}, 365^{b}	533^{a}, 528^{b}	53262.16^{b}	37	4.3

Chemical Oxidation of Cm.

In a glovebox consisting of N_2 atmosphere, dichloroethane solution of $NOSBF_6$ was added to a dichloroethane solution containing the macrocycle stepwise until a change in color was observed. The solution was then placed in an air tight cuvette, and the UV-Vis-NIR absorption spectra was measured. While C8 and C6 were sufficiently stable in the glovebox, C7 and C5 quickly decomposed, yielding a black solution which does not show the expected cation or dication peaks.

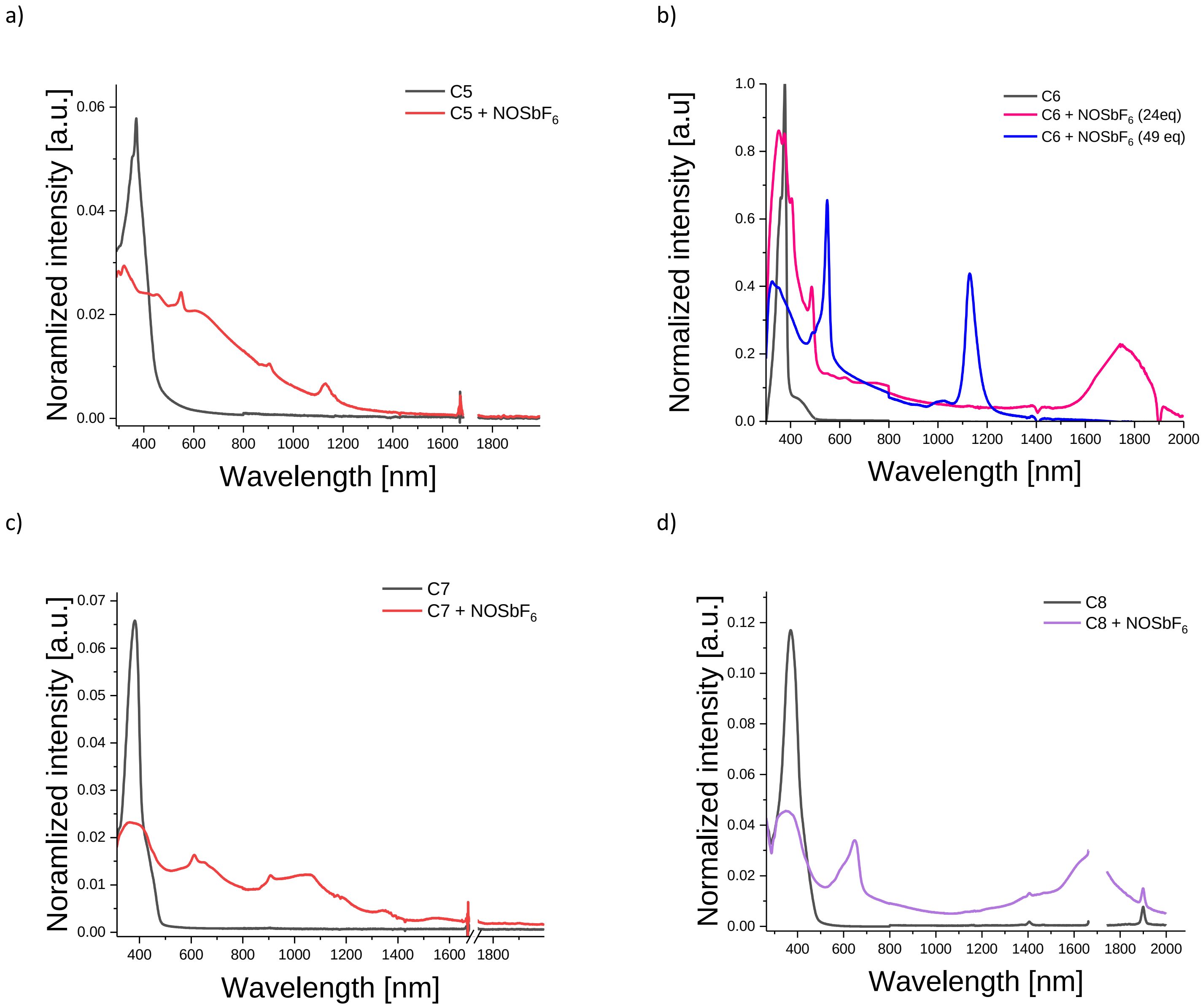

Supplementary Figure 46. UV-Vis-NIR absorption spectra of (a) **C5**, (b) **C6**, (c) **C7** and (d) **C8** upon addition of NoSbF6 as oxidant, measured in dichloroethane..

S4. Electrochemistry

For electrochemical measurements, dichloromethane containing 0.1 M tetra-n-butylammonium perchlorate (TBAPC) was used as a solvent. Ag/AgCl was used as a reference electrode by dipping a silver wire in an aqueous solution of $FeCl_3$ and HCl. Platinum-disk and platinum-wire electrodes were applied as working and counter electrodes, respectively. All electrochemical measurements were externally calibrated against the $E_{1/2}$ of the Fc/Fc^+ redox couple.

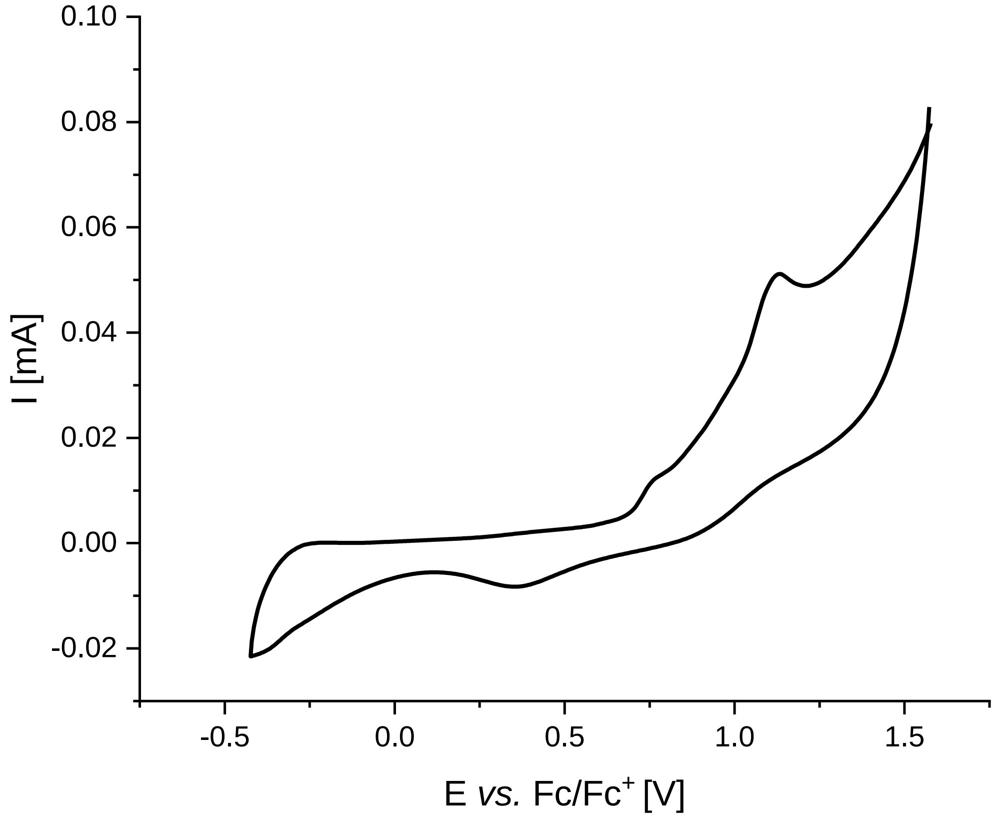

Supplementary Figure 47. Cyclic voltammetry of **C5** in dichloromethane as solvent and 0.1 M TBAPC as electrolyte, referenced against the Fc/Fc^+ redox couple (scan rate 100 mV/s).

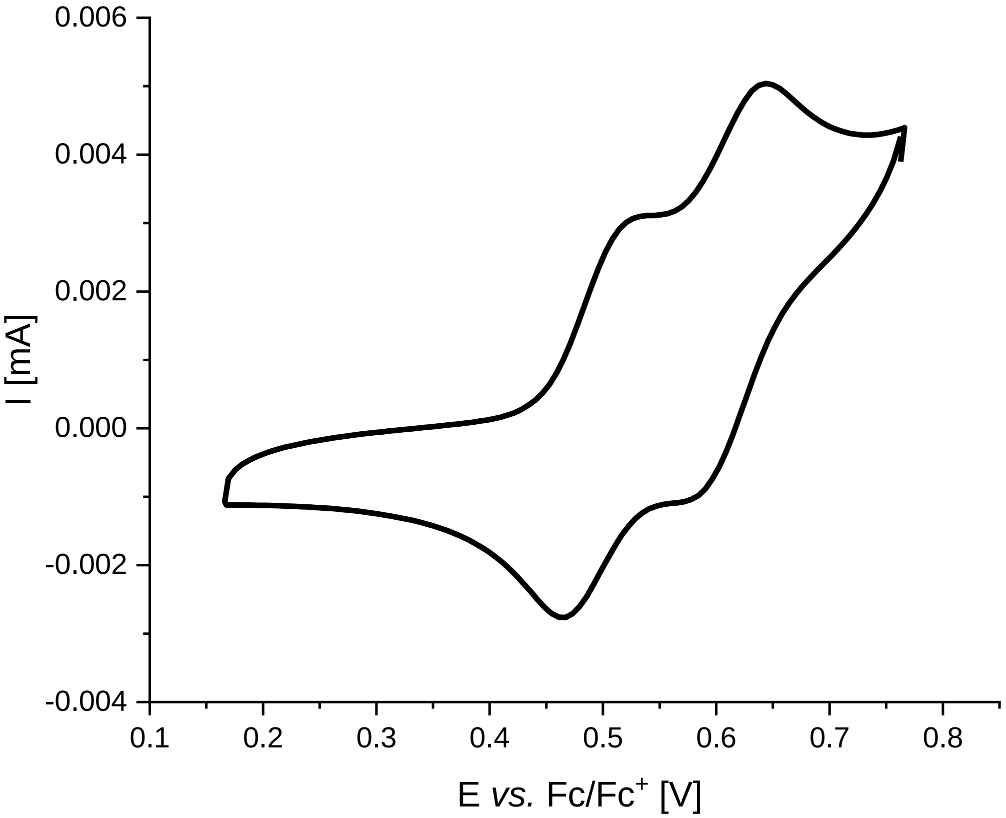

.

Supplementary Figure 48. Cyclic voltammetry of **C6** in dichloromethane as solvent and 0.1 M TBAPC as electrolyte, referenced against the Fc/Fc^+ redox couple (scan rate 100 mV/s).

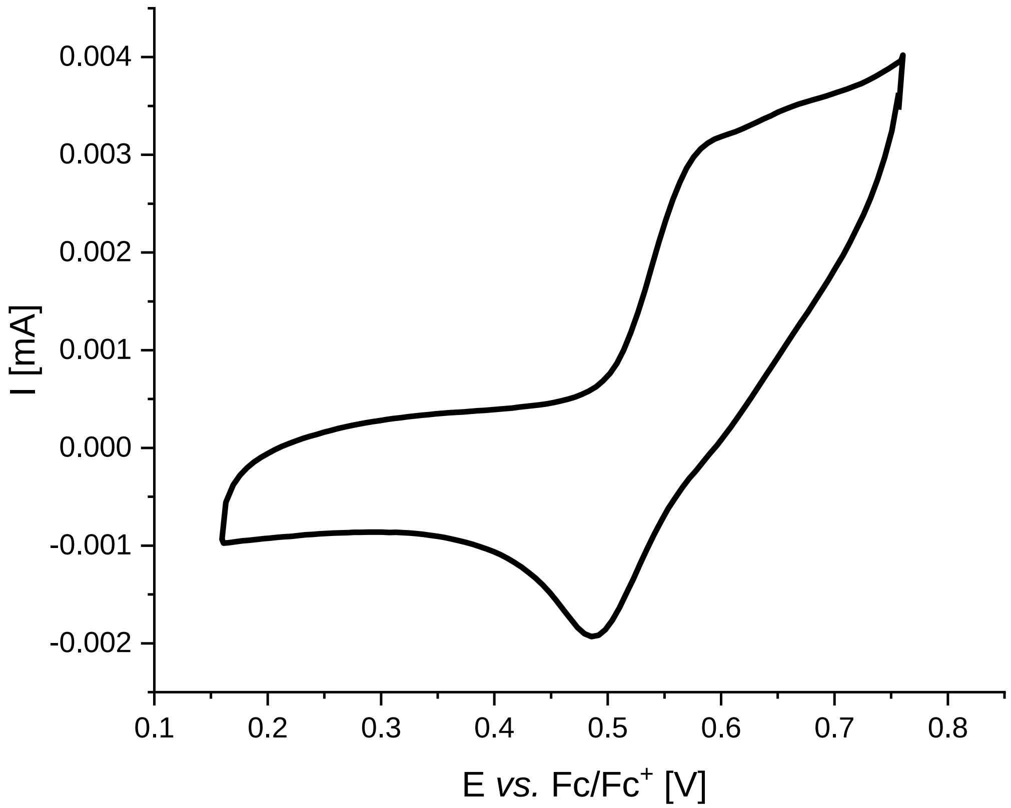

Supplementary Figure 49. Cyclic voltammetry of **C7** in dichloromethane as solvent and 0.1 M TBAPC as electrolyte, referenced against the Fc/Fc^{+} redox couple (scan rate 100 mV/s).

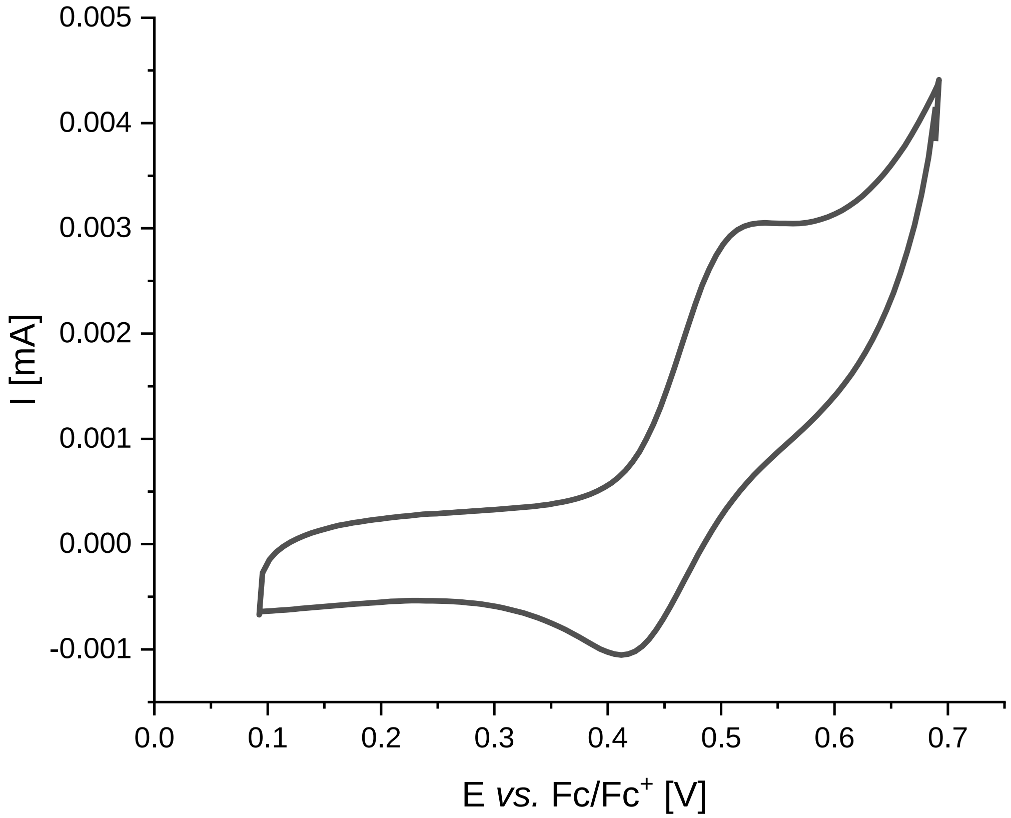

Supplementary Figure 50. Cyclic voltammetry of **C8** in dichloromethane as solvent and 0.1 M TBAPC as electrolyte, referenced against the Fc/Fc^{+} redox couple (scan rate 100 mV/s).

S5. Computational Results

All calculations were carried out with the Gaussian 16 series of programs[2] using density function theory (DFT). Becke's three-parameter exchange functional combined with the Lee-Yang-Parr correlation functional (B3LYP) and with the 6-311G(d) basis set was used for all stationary points. No symmetry restrictions were applied in any of the optimal geometries presented. The optimal geometries for all structures were confirmed as minima by frequency calculations. No negative frequencies were found for any stationary points presented in this work.

S5.1. Absolute Energies and Comparison of Different Functionals

In order to find most appropriate functional to represent the NICS values, as well as the orbital energy levels, we have compared the calculated values experimental data, obtained from electrochemistry (correlation between the oxidation potentials and HOMO levels), absorption and emission spectra (optical gaps), and ^{1}H-NMR (chemical shift). In our comparison, we have taken into account the over-delocalization of functionals with small Hartree- Fock exchange, in particular B3LYP,[3] as well as previous discussion regarding the functionals applied for NICS of long conjugated systems. We calculated the absolute and FMO energies of **Cm** (n = 5-8) using several different functionals: B3LYP, CAM-B3LYP, ωB97XD, M06 and M06-2X, all using the 6-311G(d) basis-set. We found that M06-2X was yielded the best match with the experimental chemical shifts, in addition with the best match with the observed trend between macrocycles. For orbital energies, M06 (with 27% HF exchange) gave the best match between the HOMO energy levels and the observed oxidation potentials. We note that the all functionals yielded the same alternating trend between even and odd-membered macrocycles.

Supplementary Table 3. Absolute energies (Hartree) of **Cm** calculated using the B3LYP, CAM-B3LYP, ωB97XD and M06 functional with the 6-311G(d) basis set.

	B3LYP	CAM-B3LYP	ωB97XD	M06	M06-2X
**C5**	-1721.923461	-1721.024507	-1721.284037	-1720.7120379	-1721.238814
**C6**	-2066.3190841	-2065.2413214	-2065.5530110	-2064.8662738	-2065.498849
**C7**	-2410.7102623	-2409.4519186	-2409.8159608	-2409.0154868	-2409.752888
**C8**	-2755.0969106	-2753.6588605	-2754.0755971	-2753.1606912	-2754.003459

Supplementary Table 4. Energies for select MOs of **Cm** (n=5–8) using different functionals using the 6-311G(d) basis set. The energies shown in the table are for the α orbitals and were identical to the energies of the corresponding ß orbitals.

	Functional \ MO	Energy (eV)					
		HOMO-2	HOMO-1	HOMO	LUMO	LUMO+1	LUMO+2
**C5**	B3LYP	-6.45	-5.11	-5.11	-2.08	-2.08	-0.83
	CAM-B3LYP	-7.78	-6.26	-6.26	-1.05	-1.05	0.36
	ωB97XD	-8.34	-6.77	-6.77	-0.51	-0.51	0.94
	M06	-6.76	-5.41	-5.41	-1.99	-1.99	-0.72
	M06-2X	-7.73	-6.21	-6.21	-1.35	-1.35	0.06
**C6**	B3LYP	-5.53	-5.53	-4.81	-2.39	-1.69	-1.69

	CAM-B3LYP	-6.72	-6.72	-5.95	-1.36	-0.62	-0.62
	ωB97XD	-7.25	-7.25	-6.45	-0.82	-0.07	-0.07
	M06	-5.83	-5.83	-5.12	-2.28	-1.60	-1.60
	M06-2X	-6.68	-6.68	-5.90	-1.66	-0.93	-0.93
**C7**	B3LYP	-5.86	-4.96	-4.96	-2.25	-2.25	-1.39
	CAM-B3LYP	-7.10	-6.10	-6.10	-1.23	-1.23	-0.29
	ωB97XD	-7.64	-6.61	-6.61	-0.70	-0.70	0.28
	M06	-6.17	-5.27	-5.27	-2.16	-2.16	-1.30
	M06-2X	-7.05	-6.06	-6.06	-1.54	-1.54	-0.59
**C8**	B3LYP	-5.26	-5.26	-4.79	-2.44	-1.98	-1.98
	CAM-B3LYP	-6.42	-6.42	-5.93	-1.42	-0.95	-0.95
	ωB97XD	-6.93	-6.93	-6.43	-0.89	-0.41	-0.41
	M06	-5.57	-5.57	-5.11	-2.34	-1.89	-1.89
	M06-2X	-6.37	-6.37	-5.88	-1.72	-1.25	-1.25

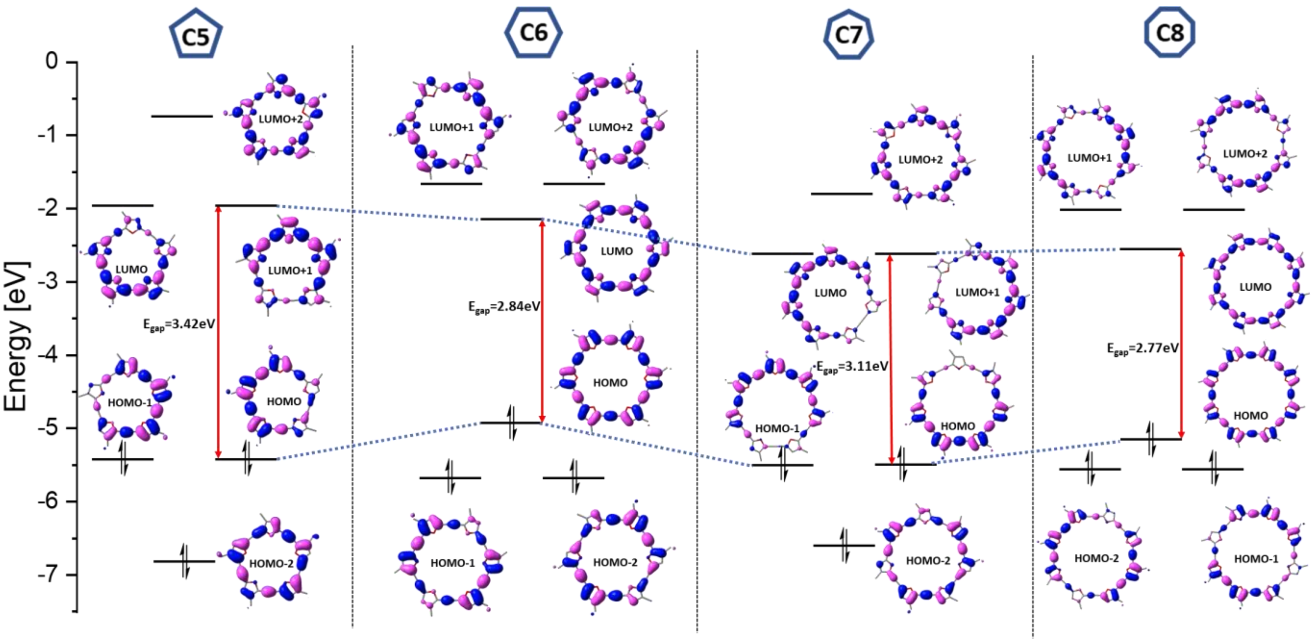

Supplementary Figure 51. MO diagram for (a) **C5**, (b) **C6**, (c) **C7** and (d) **C8** at M06/6-311G(d) level.

a)

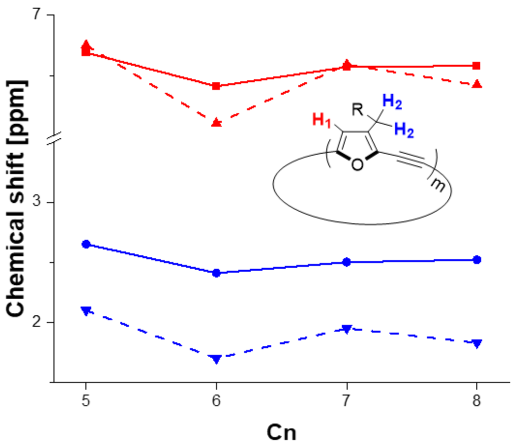

b)

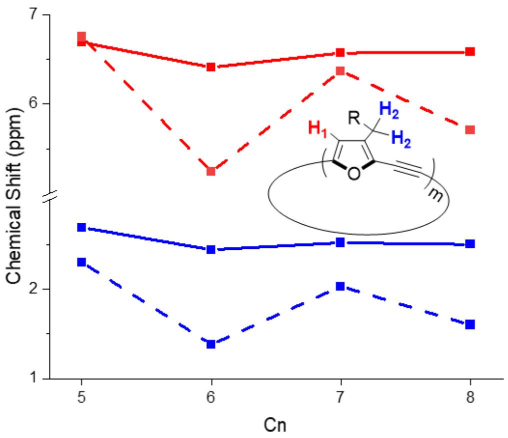

c)

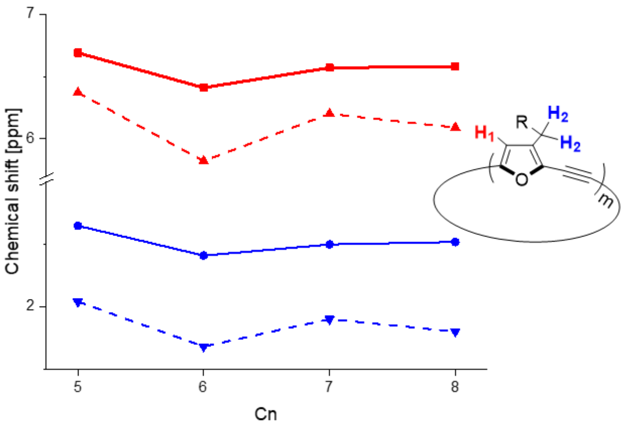

Supplementary Figure 52. Experimental and calculated (solid and dashed traces, respectively) ^{1}H-NMR of the H_1 and H_2 protons (red and blue, respectively) in each **Cm**, calculated using in (a) M06-2X, (b) ωB97XD and (c) CAM-B3LYP functionals with the 6-311G(d) basis set.

S5.2ACID plots:

Anisotropy of the current (induced) density (ACID)[4] plots use the current density (yellow surface) and the current density vectors (green arrows) as a method of quantifying π-conjugation and, if present, paratropic and diatropic ring currents, where counter-clockwise current vectors indicate a paratropic ring current (antiaromatic system), and clockwise vectors indicate a diatropic current (aromatic system). All plots were calculated in ωB97XD functional with the 6-311G(d) basis set.

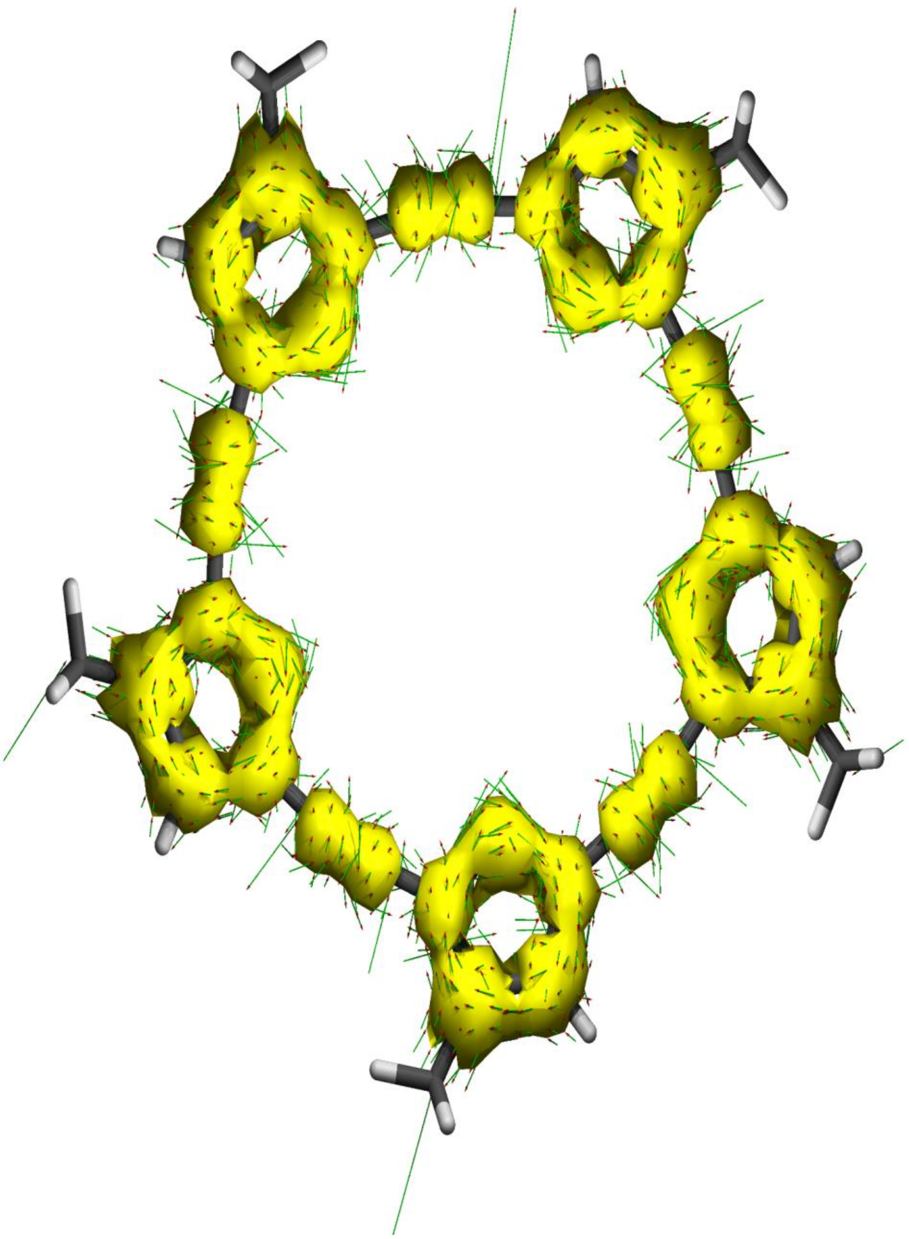

Supplementary Figure 53. ACID plot for **C5**.

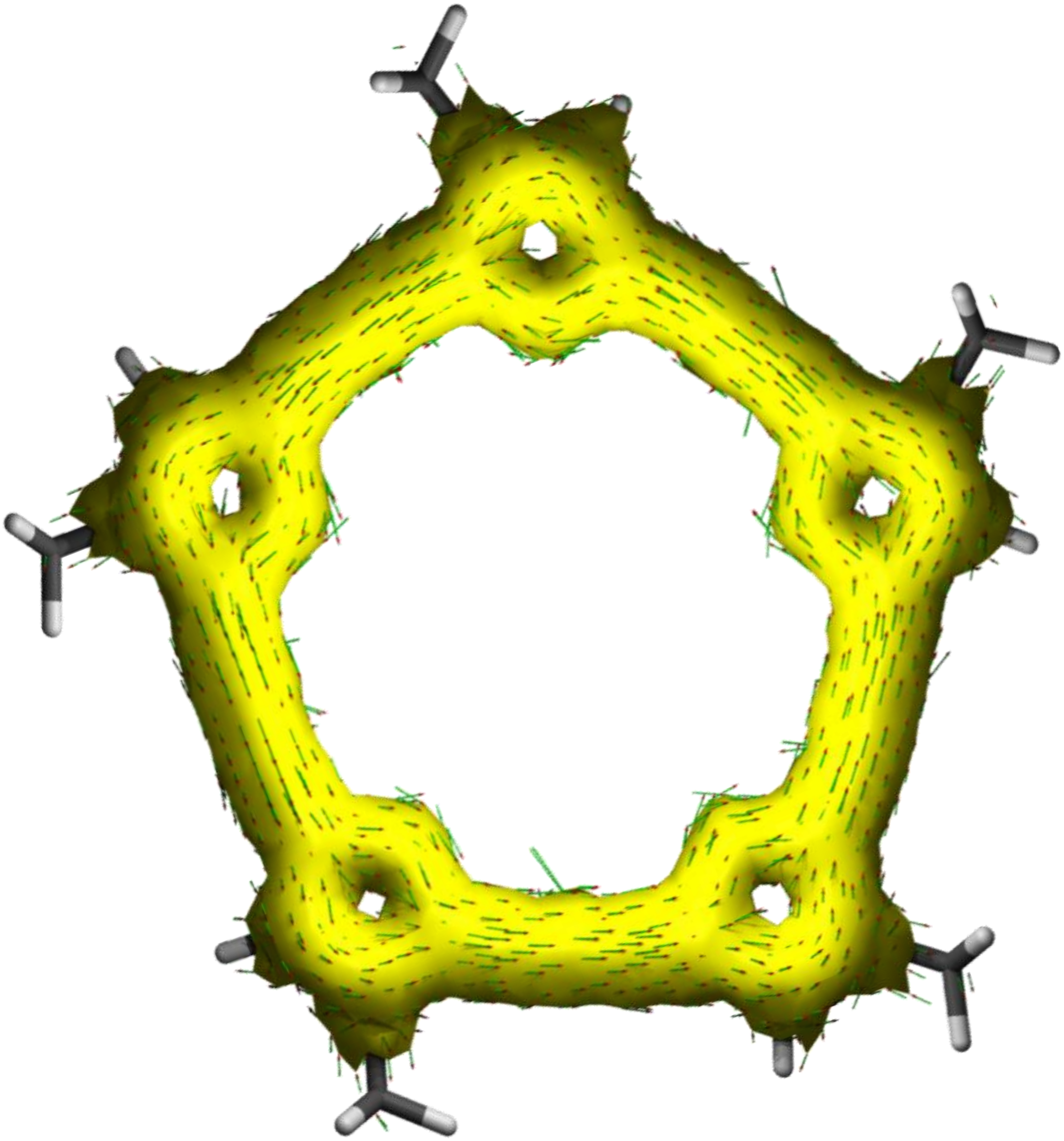

Supplementary Figure 54. ACID plot for **C5**$^{+2}$.

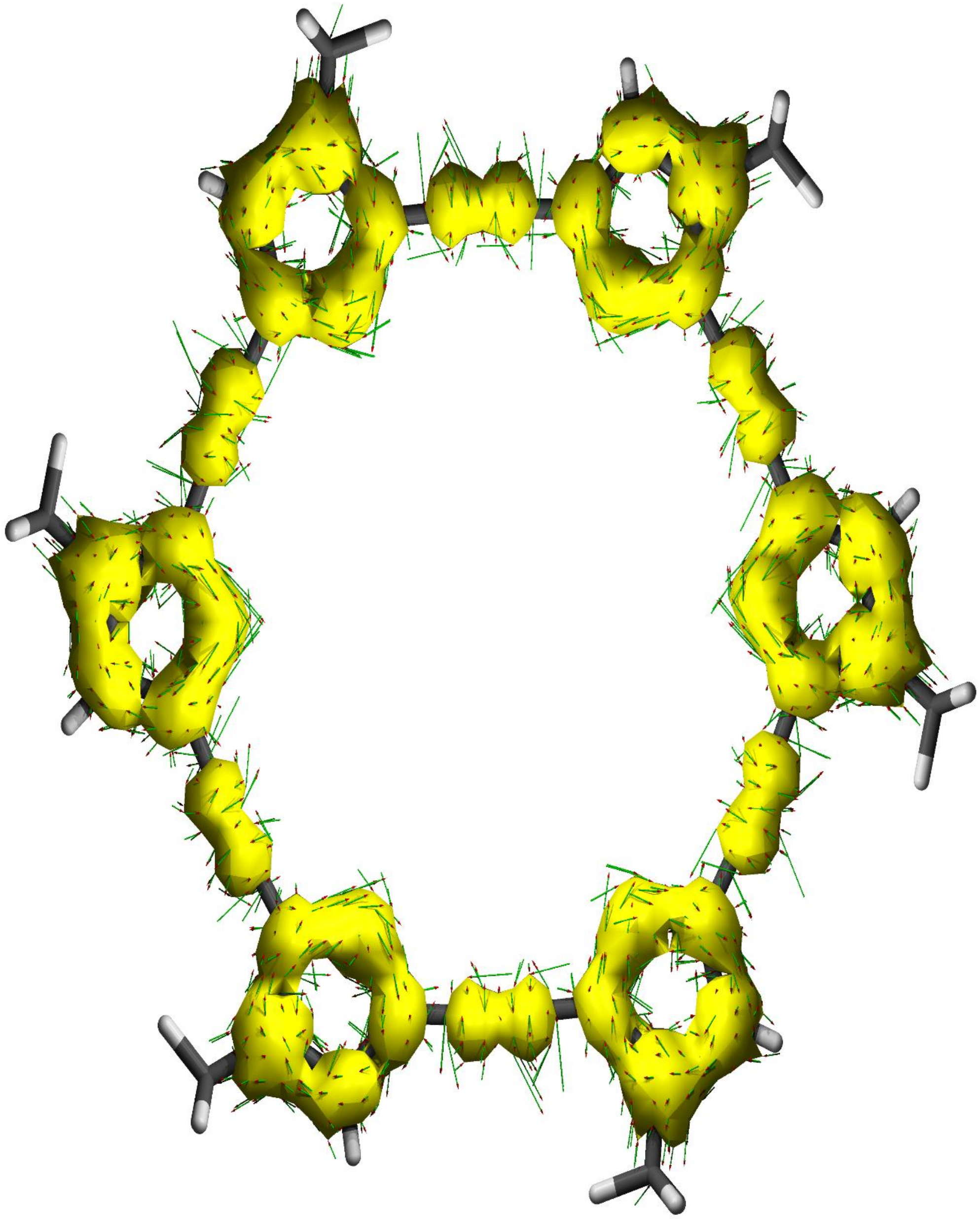

Supplementary Figure 55. ACID plot for **C6.**

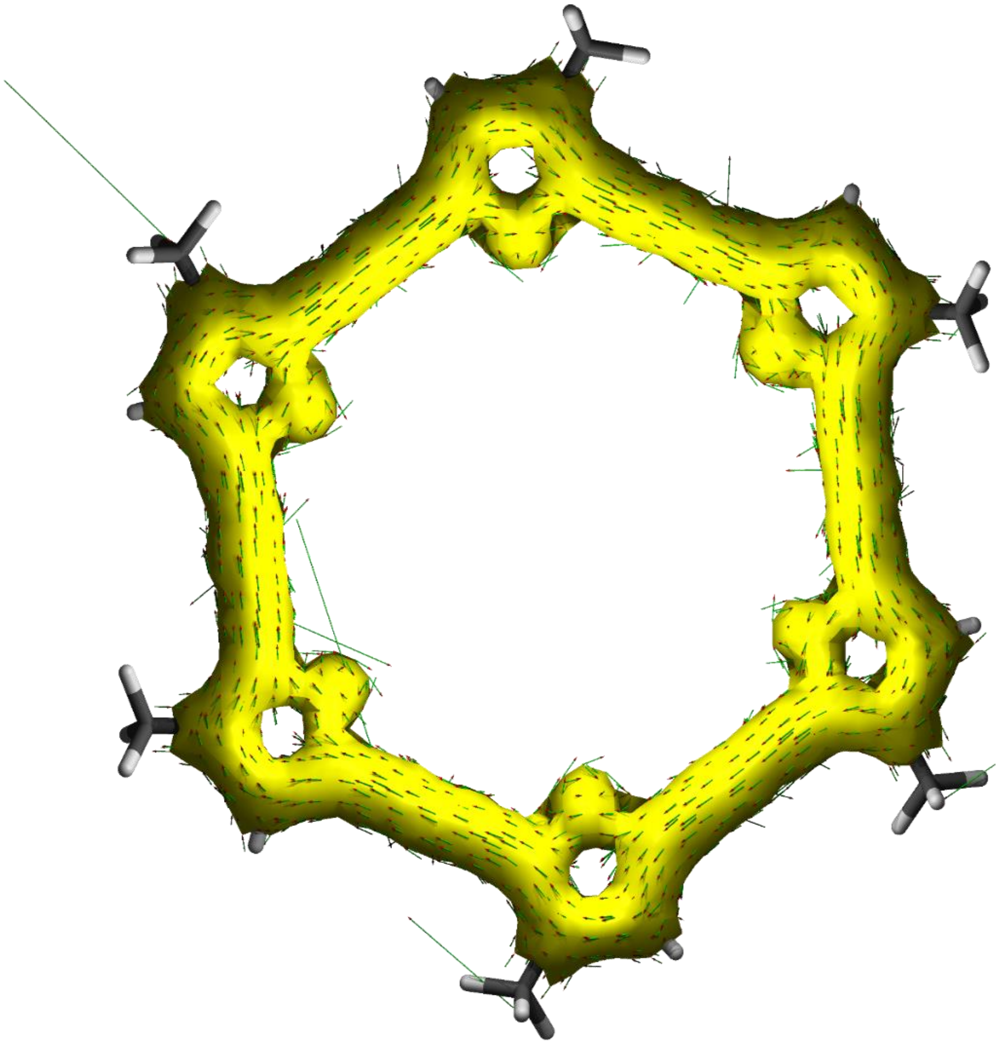

Supplementary Figure 56. ACID plot for **C6**$^{+2}$.

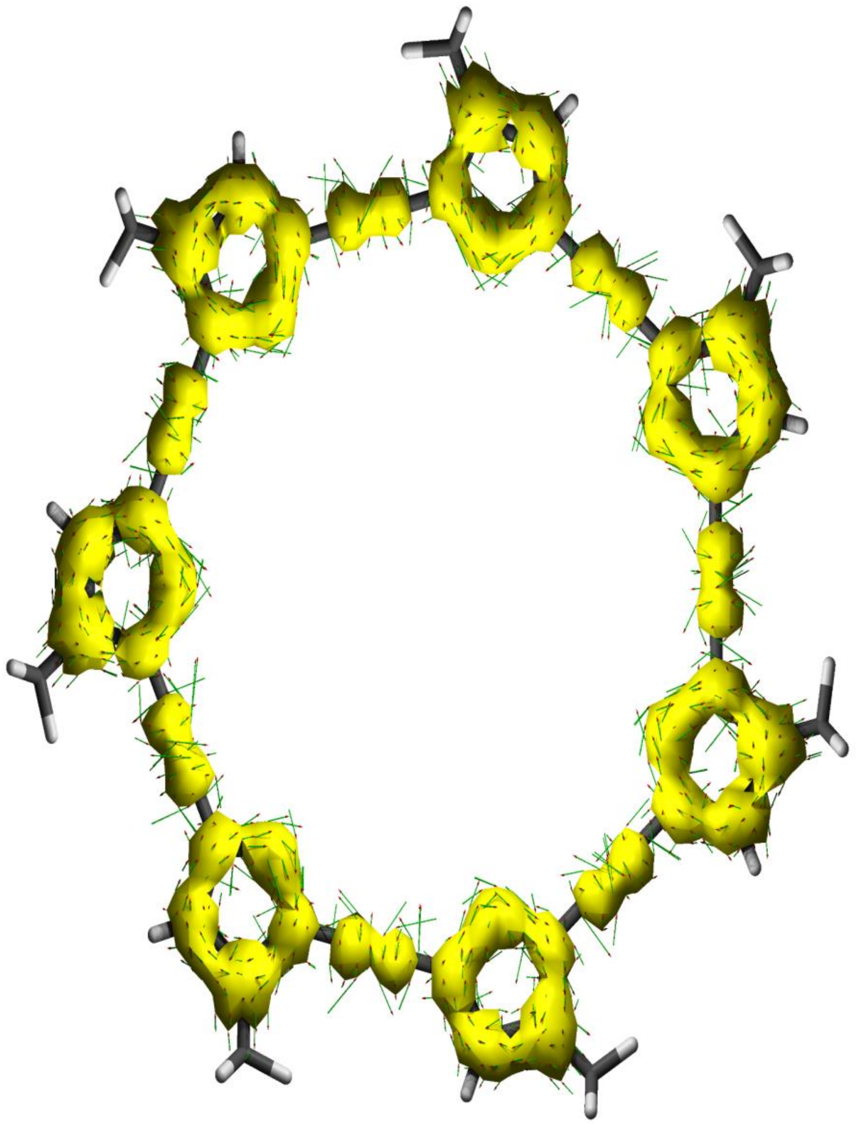

Supplementary Figure 57. ACID plot for **C7**.

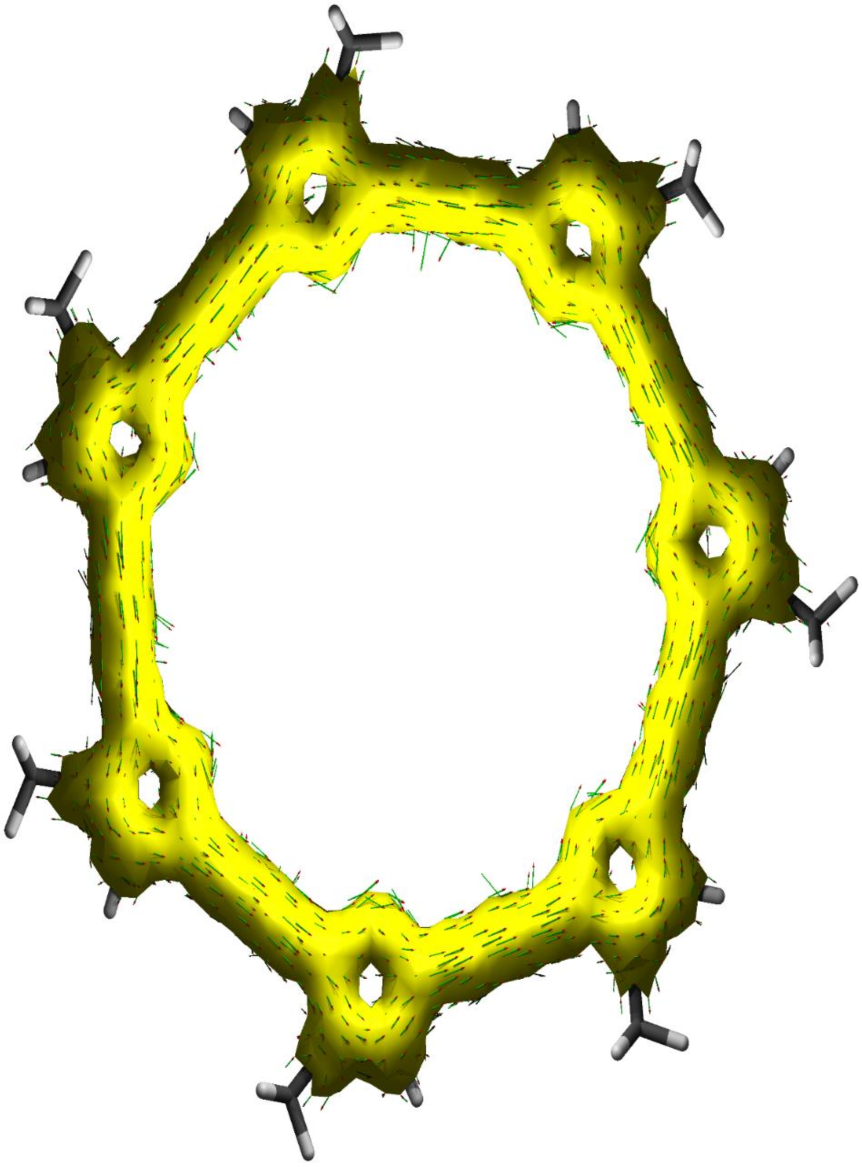

Supplementary Figure 58. ACID plot for **C7^{+2}**.

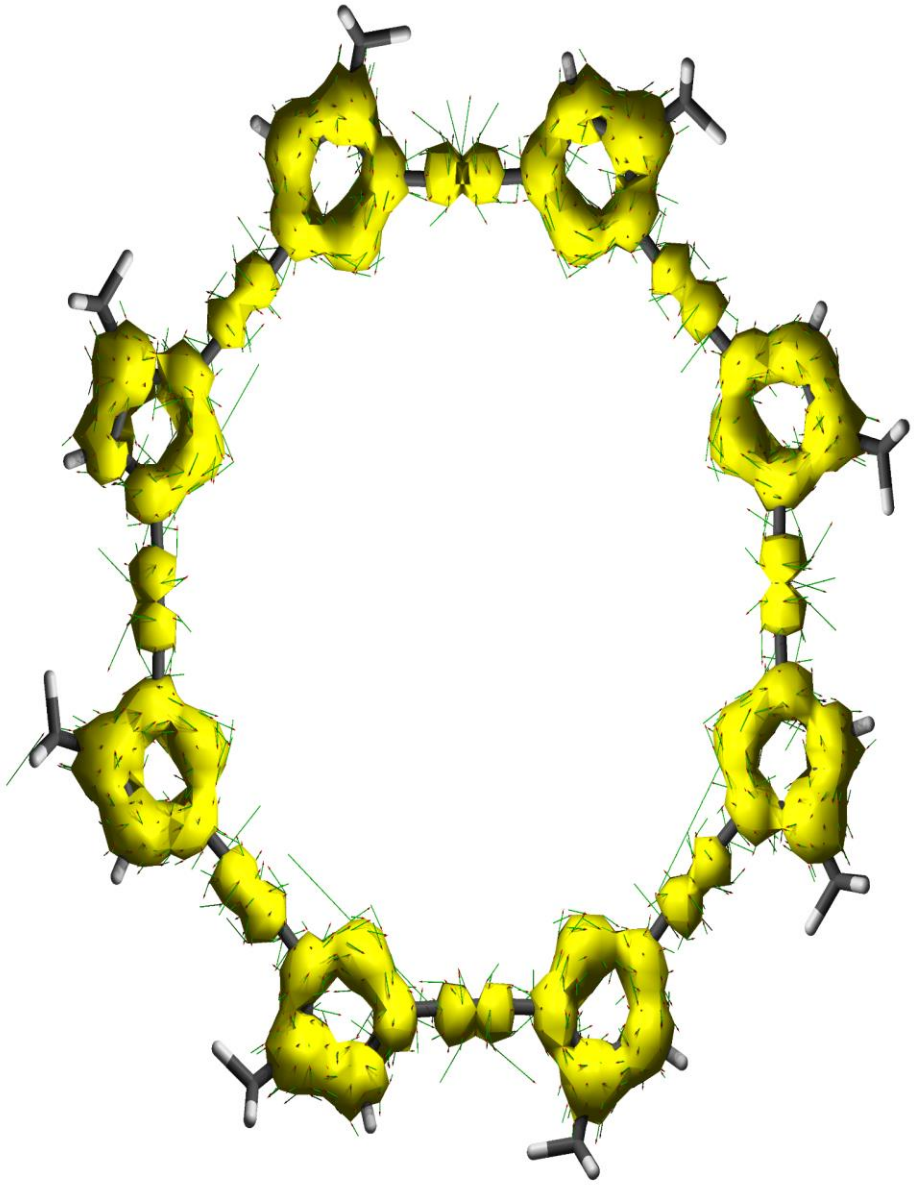

Supplementary Figure 59. ACID plot for **C8**.

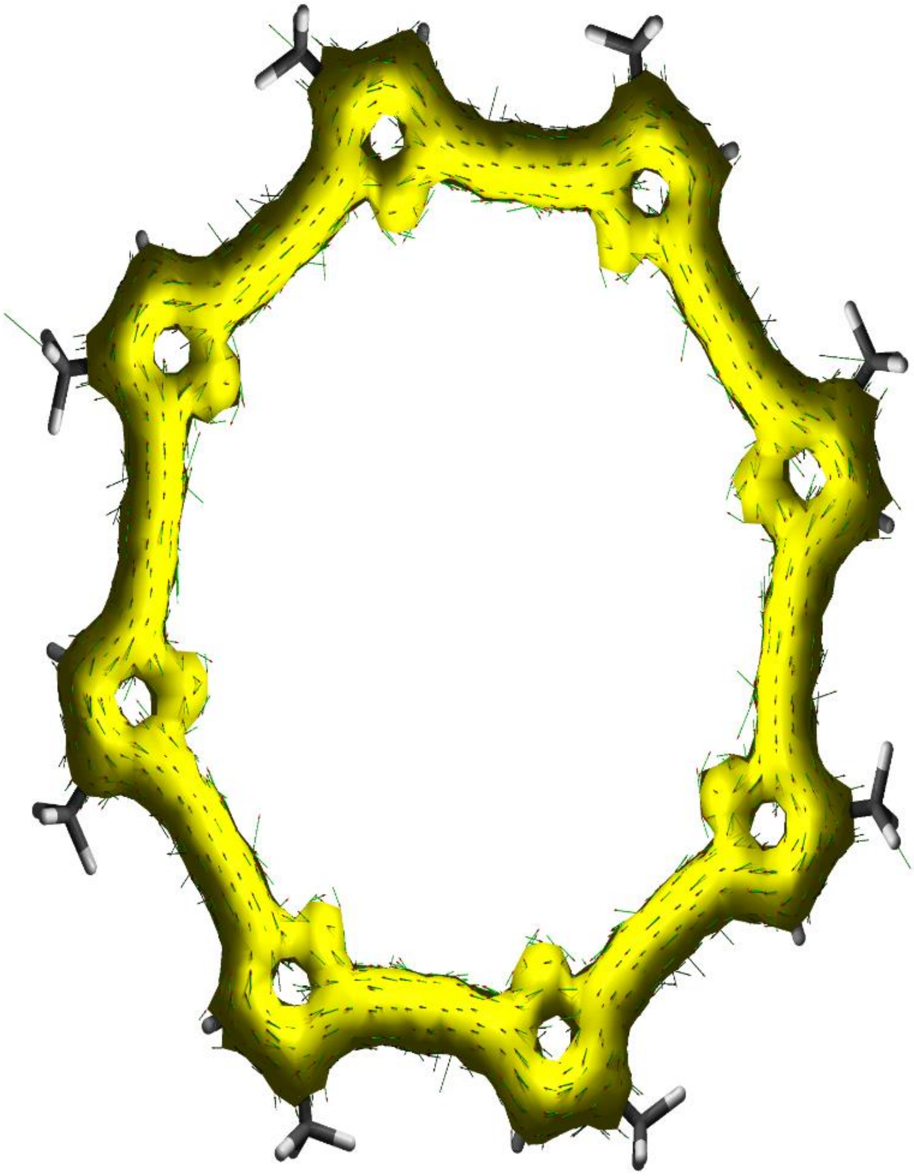

Supplementary Figure 60. ACID plot for $\mathbf{C8}^{+2}$.

S5.3. NICS

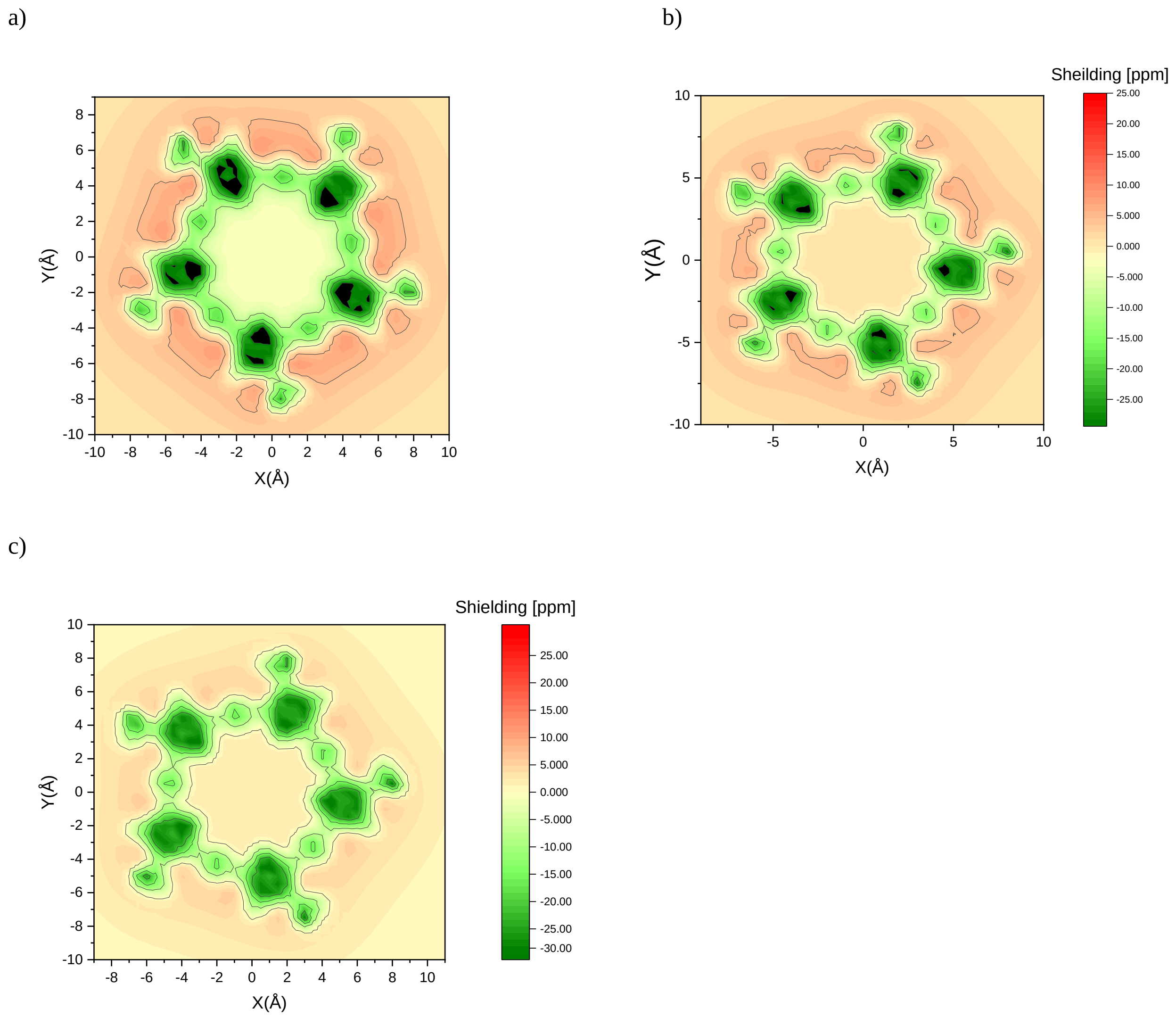

Supplementary Figure 61. $NICS_{zz}$ map on the Z = 1 plane of **C5** calculated using (a) B3LYP, (b) M06-2X and (c) ωB97XD functionals with the 6-311G(d) basis set.

a)

b)

c)

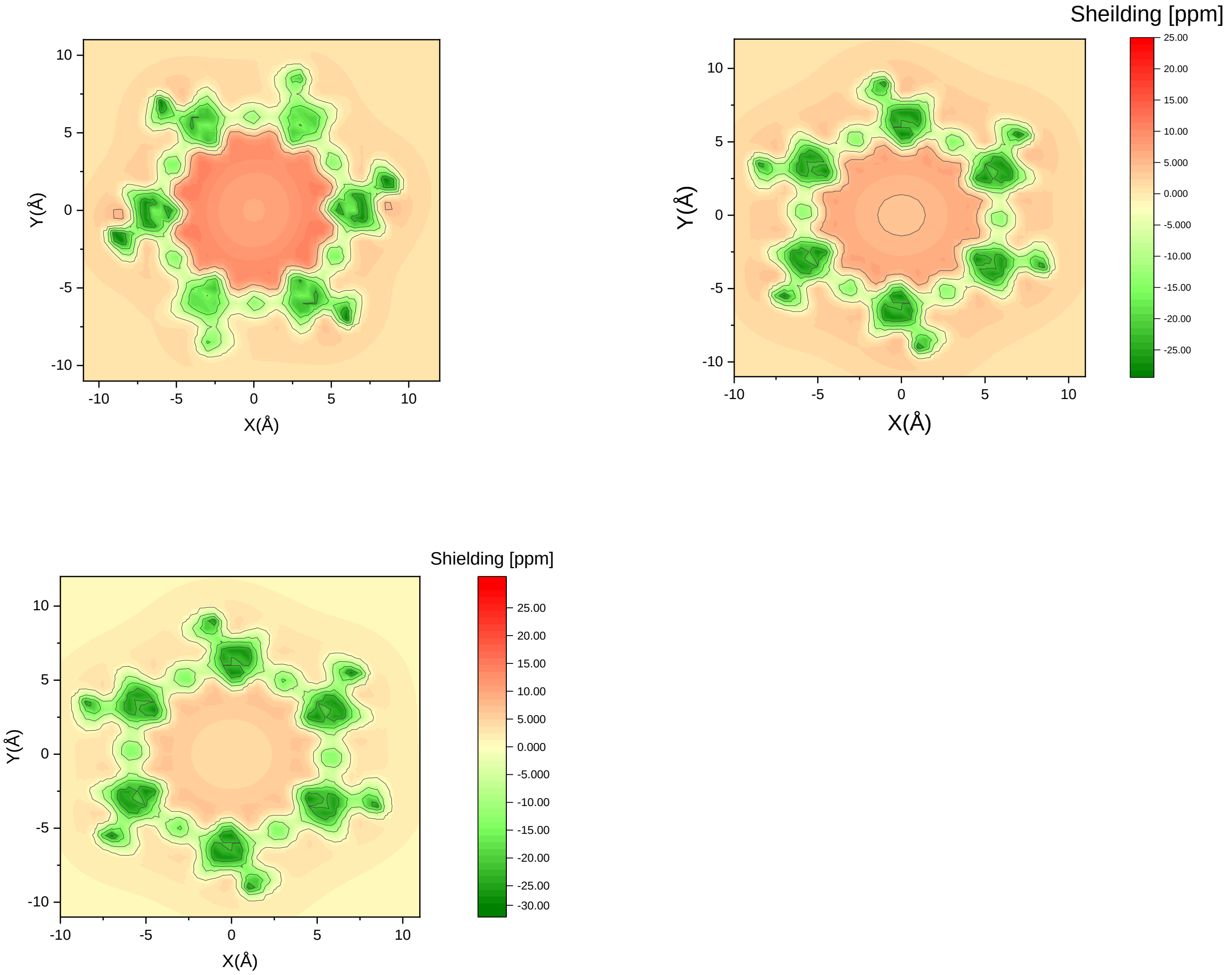

Supplementary Figure 62. NICS$_{zz}$ map on the Z = 1 plane of **C6** calculated using (a) B3LYP, (b) M06-2X and (c) ωB97XD functionals with the 6-311G(d) basis set.

a)

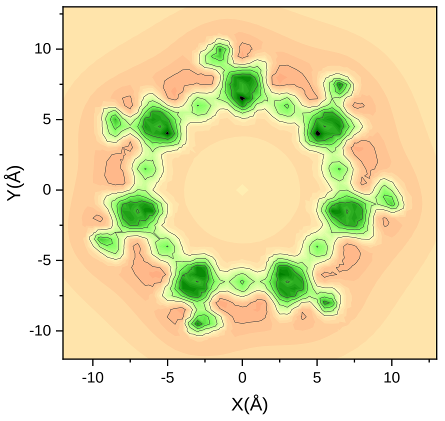

b)

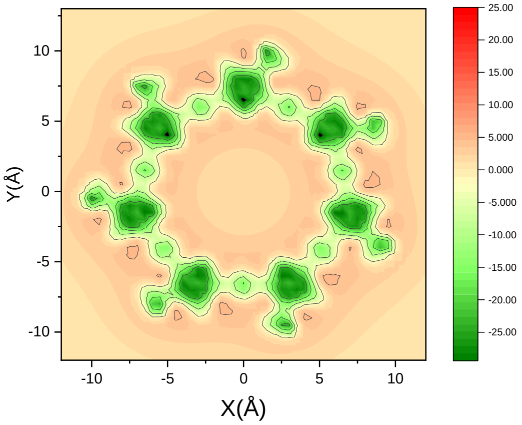

c)

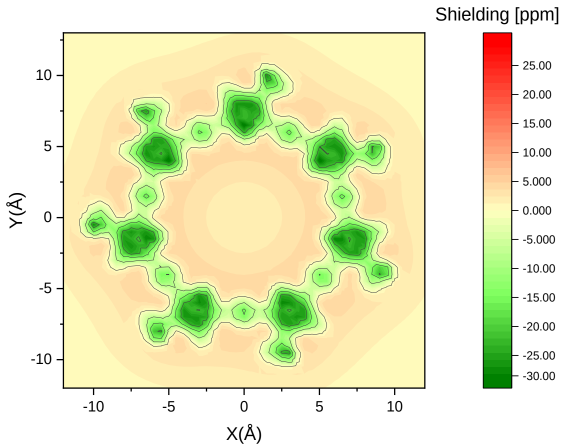

.

Supplementary Figure 63. $NICS_{zz}$ map on the Z = 1 plane of **C7** calculated using the (a) B3LYP, (b) M06-2X and (c) ωB97XD functionals with the 6-311G(d) basis set.

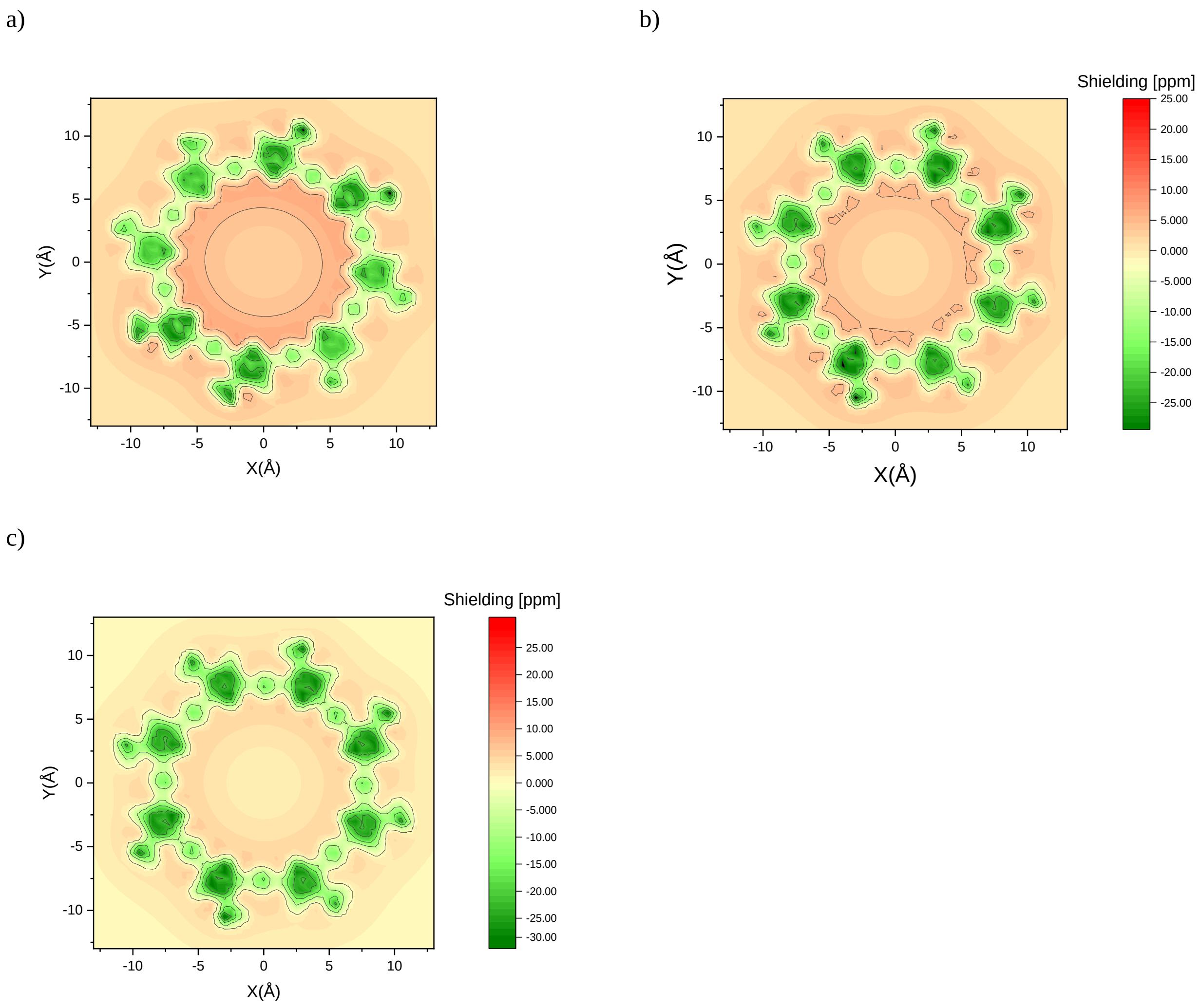

Supplementary Figure 64. $NICS_{zz}$ map on the Z = 1 plane of **C8** calculated using (a) B3LYP, (b) M06-2X and (c) ωB97XD functionals with the 6-311G(d) basis set.

a)

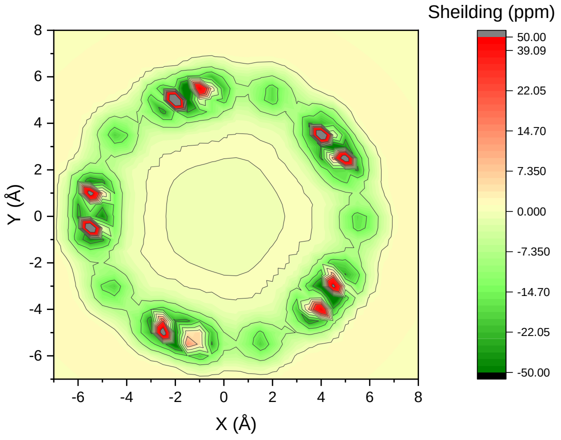
Sheilding (ppm)
X (Å)
Y (Å)

b)

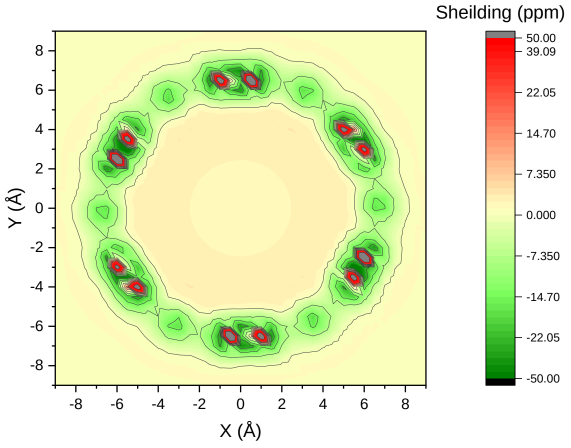
Sheilding (ppm)
X (Å)
Y (Å)

c)

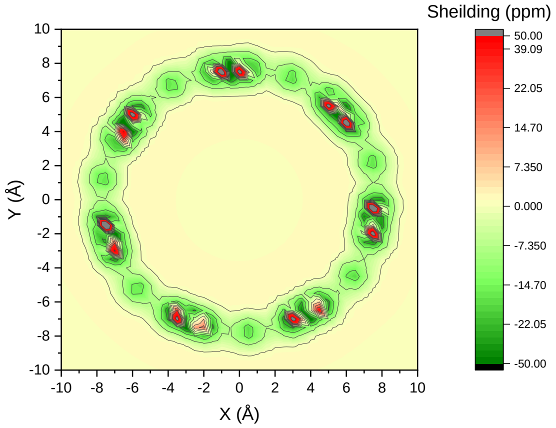
Sheilding (ppm)
X (Å)
Y (Å)

d)

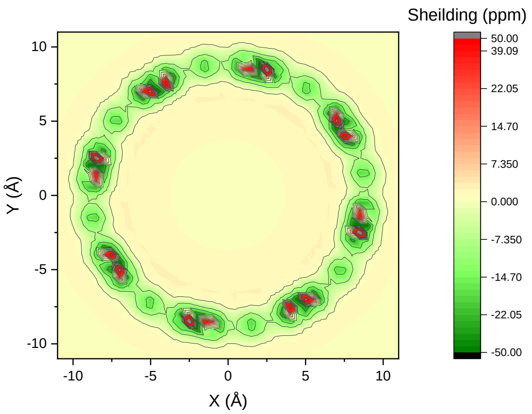
Sheilding (ppm)
X (Å)
Y (Å)

e)

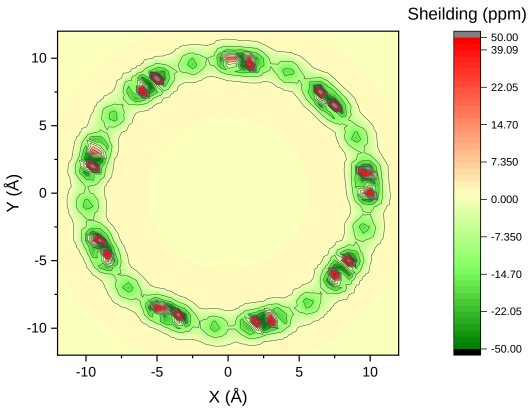
Sheilding (ppm)
X (Å)
Y (Å)

f)

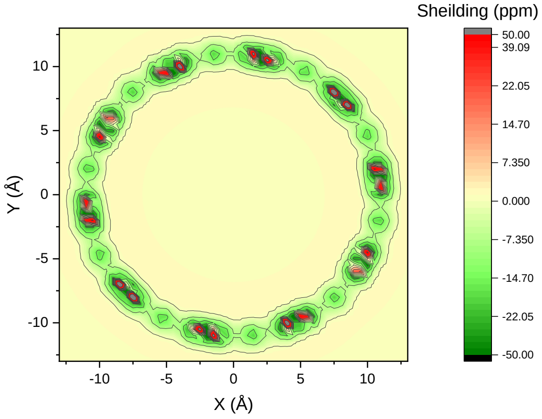
Sheilding (ppm)
X (Å)
Y (Å)

g)

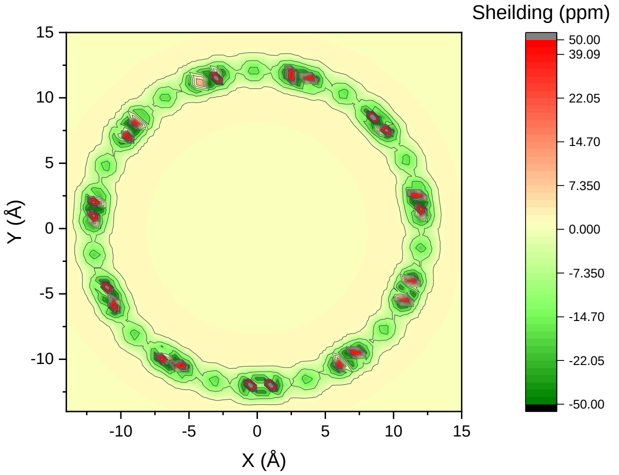

h)

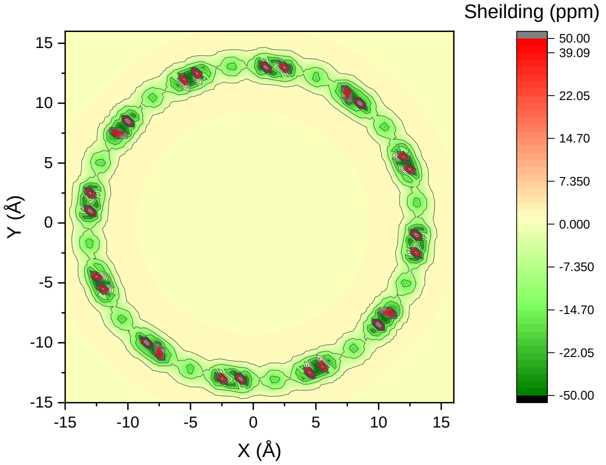

Supplementary Figure 65. NICS$_{zz}$ map on the Z = 1 plane of **[n]-CPPA** calculated using M06-2X and functional with the 6-311G(d) basis set. a) **[5]-CPPA,** b) **[6]- CPPA,** c) **[7]- CPPA,** d) **[8]- CPPA,** e) **[9]- CPPA,** f) **[10]- CPPA,** g) **[11]- CPPA and** h) **[12]- CPPA.**

Supplementary Table 5. NICS(1) values for **Cm**, **[m]-CPPA** and **[m]-TA** (thiophene-acetylene cycles, see Chart 1) in their ground state calculated using the B3LYP, CAM-B3LYP, ωB97XD and M06-2X functionals using the 6-311G(d) basis set.

	n	B3LYP	CAM-B3LYP	ωB97XD	M06-2X
**Cm**	5	-11.0804	-10.16723	-10.11413	-10.85469
	6	-7.54073	-8.83443	-9.03255	-9.32466
	7	-9.71724	-9.46938	-9.51686	-10.17959
	8	-7.49404	-9.02036	-9.25049	-9.60427
	9	-9.41307	-9.28883	-9.43307	-9.98024
	10	-8.71701	-9.22164	-9.37833	-9.9241
	11	-8.94005	-9.23345	-9.40625	-9.92679
	12	-8.84186	-9.24131	-9.33269	-9.91854
**[m]CPPA**	5	-10.3089	-10.16723	-10.44545	-10.79455
	6	-9.2407	-8.83443	-10.1119	-10.1864
	7	-9.7093	-9.46938	-10.2065	-10.45475
	8	-9.54865	-9.02036	-10.14815	-10.4102
	9	-9.6176	-9.28883	-10.16115	-10.41455
	10	-9.5528	-9.22164	-10.197	-10.37645
	11	-9.5384	-9.23345	-10.25675	-10.3865
	12	-9.5693	-9.24131	-10.1694	-10.371
**[m]-TA**	5	-11.0541	-10.19732	-10.01	-10.96492
	6	-7.63986	-8.94892	-9.03748	-9.53317
	7	-9.8828	-9.67527	-9.55793	-10.41529
	8	-8.66753	-9.458	-9.41803	-10.00704
	9	-9.41307	-9.59878	-9.40906	-10.39368
	10	-9.00668	-9.56772	-9.34977	-10.33016
	11	-9.26631	-9.61102	-9.35692	-10.4459
	12	-9.19425	-9.64454	-9.35965	-10.5415

a)

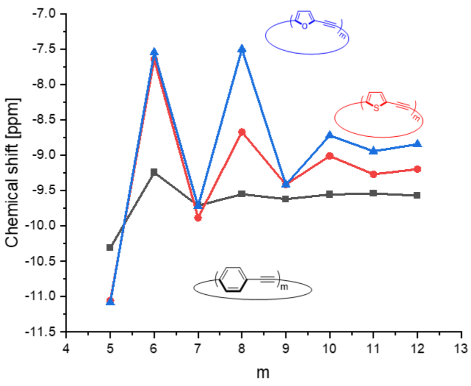

b)

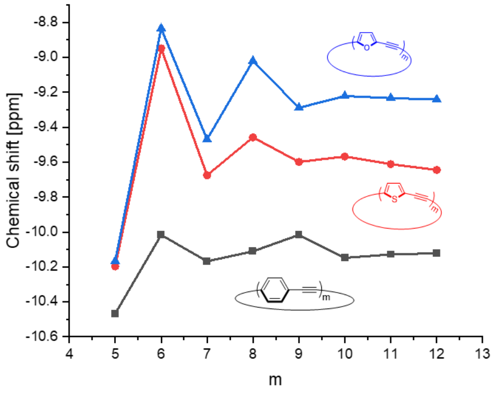

c)

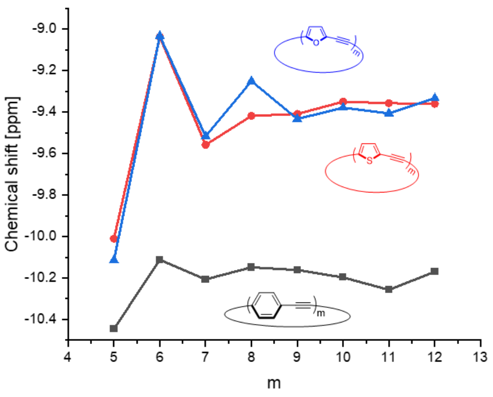

d)

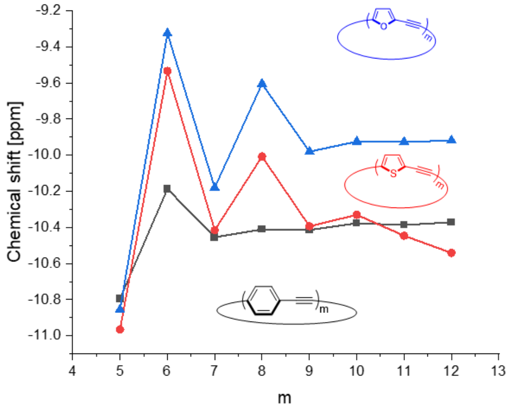

Supplementary Figure 66. NICS(1)values averaged for all furan subunits in neutral **Cm**, **[m]-TA** and **[m]-CPPA** calculated using (a) B3LYP, (b) CAM-B3LYP, (c) ωB97XD and (d) M06-2X functional with the 6-311G(d) basis set.

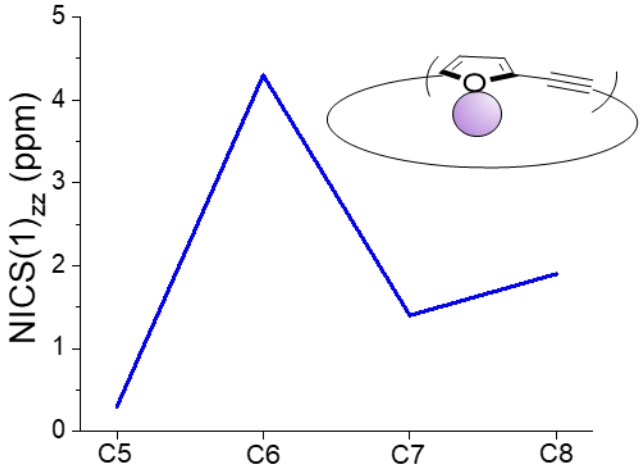

Supplementary Figure 67. NICS(1)zz values for **C*m*** (**C5**–**C8**), taken from the center of each macrocycle.

S.5.4 Visualization of Chemical Shielding Tensors (VIST)

Visualization of Chemical shielding tensors were computed using TheoDORE 2.4 wavefunction analysis package available and visual molecular dynamic applied for graphic representation according to the work reported by Glöcklhofer and Plasser.[5]

a)

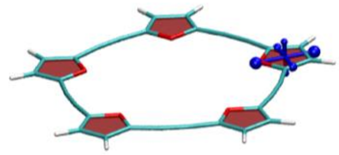

b)

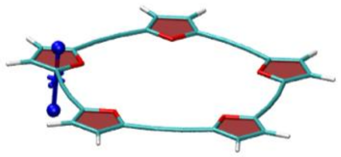

c)

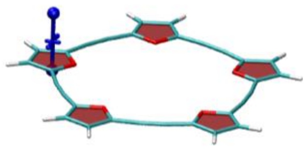

d)

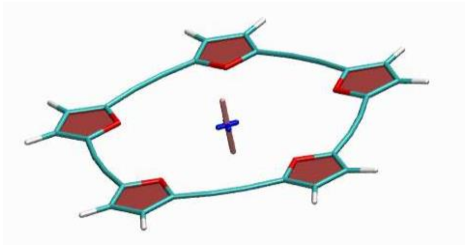

Supplementary **Figure 68. 3D visualization of the chemical shielding tensors (VIST) in C5.**

a)

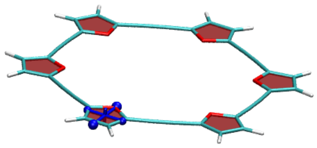

b)

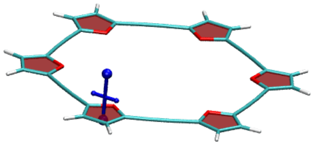

c)

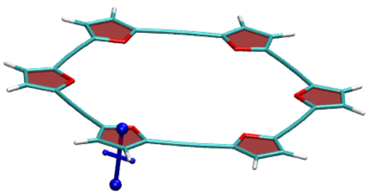

d)

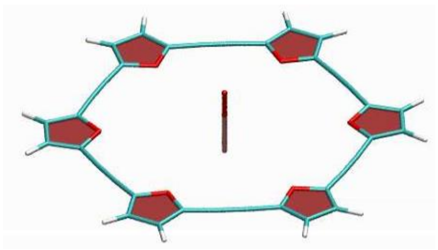

Supplementary **Figure 69. 3D visualization of the chemical shielding tensors (VIST) in C6.**

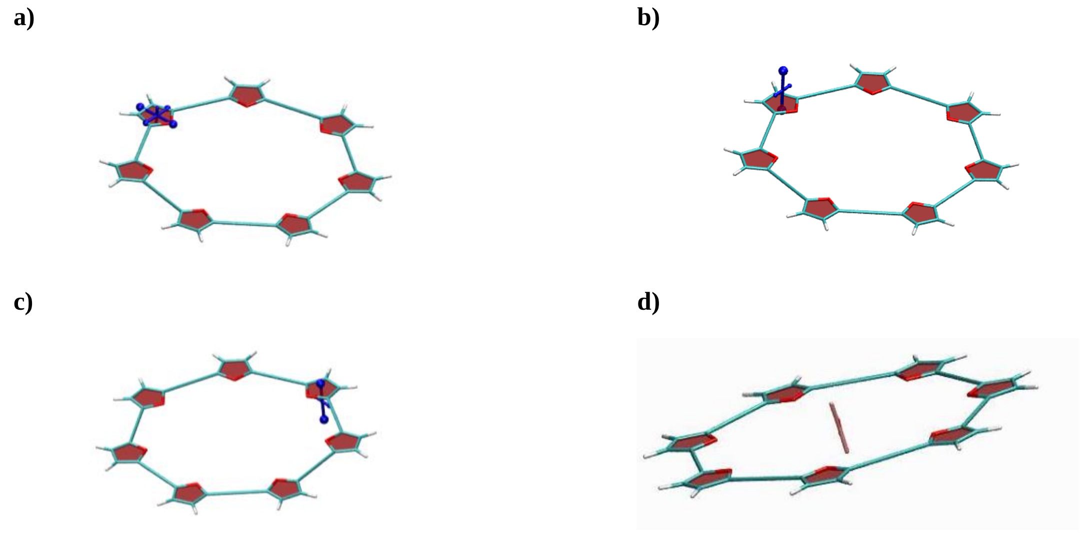

Supplementary **Figure 70. 3D visualization of the chemical shielding tensors (VIST) in C7.**

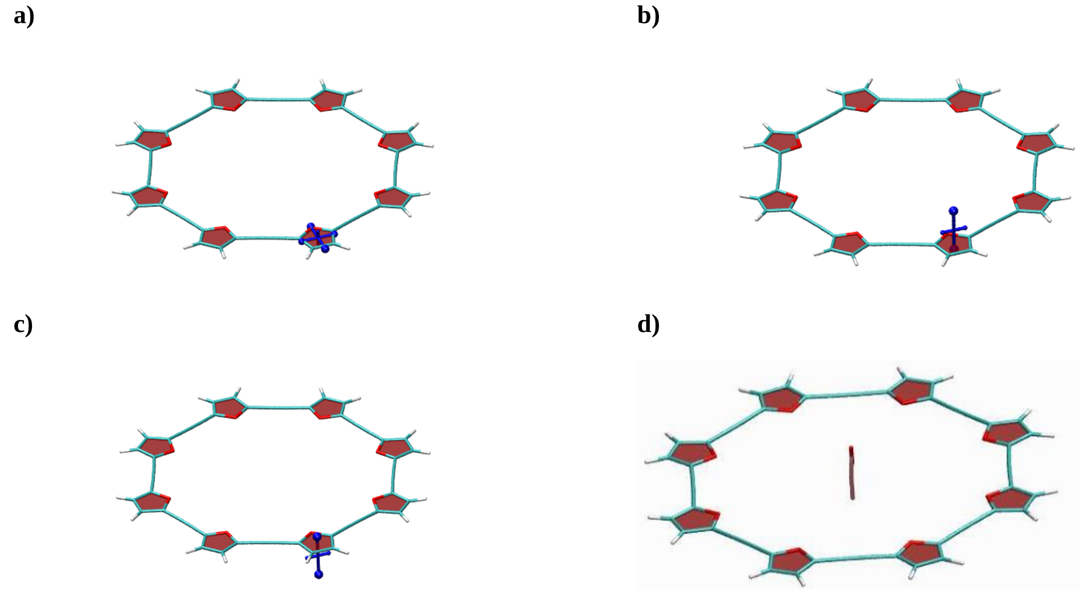

Supplementary **Figure 71. 3D visualization of the chemical shielding tensors (VIST) in C8.**

S5.4. Strain Energies

The ring strain energies were calculated as the difference between the total energy of each macrocycle and the energies of the repeating units that composed them. The energy of a single repeat unit was calculated using periodic boundary conditions (PBC) for a dimer. The energy of a single repeating unit is defined as half the energy calculated using the PBC.
The strain energies were calculated according to the following equation:

$$E_{strain} = E_{C_n} - E_{monomer} \times n$$

Where E_{Cm} is the calculated energy of **Cm**, $E_{monomer}$ is the calculated value of one repeat unit as detailed above and *n* is the number of monomers.

Supplementary Table 6. Calculated strain energies for **Cm** (n = 5-8) per repeat unit

**Molecule**	**Overall Strain Energy (kcal mol^{-1})**	**Strain Energy Per Repeat Unit (kcal mol^{-1})**
**C5**	10.16	2.03
**C6**	5.22	0.87
**C7**	3.10	0.44
**C8**	3.88	0.48

S5.5. TD spectra

Supplementary Table 7. All TD-DFT calculation were performed at the CAM-B3LYP/6-311G-(d) level to overcome the over delocalization in B3LYP functional. Emission were calculated form the optimized structure of the first singlet excited state (S_1).

Molecule	Calculated emission ($S_1 \rightarrow S_0$) [nm]
C5	478
C6	544
C7	494
C8	505

S6. X-Ray Crystallography

Supplementary Table 8. Crystallographic data and refinement parameters for crystalline **Cm**.

**Identification code**	**C6**	**C7**
Empirical formula	$C_{72}H_{84}O_6$	$C_{84}H_{88}O_7$
Formula weight	1045.39	1209.54
Temperature/K	149.98(11)	149.98(10)
Crystal system	triclinic	monoclinic
Space group	P-1	C2/c
a/Å	11.2832(11)	25.814(3)
b/Å	12.4309(10)	23.0474(15)
c/Å	13.0840(9)	27.536(3)
α/°	109.323(7)	90
β/°	110.863(8)	115.427(13)
γ/°	100.650(7)	90
Volume/Å^3	1519.9(2)	14796(3)
Z	1	8
ρ_{calc}g/cm^3	1.142	1.086
μ/mm^{-1}	0.071	0.068
F(000)	564.0	5184.0
Crystal size/mm^3	0.16 × 0.07 × 0.03	0.28 × 0.25 × 0.05
Radiation	Mo Kα (λ = 0.71073)	Mo Kα (λ = 0.71073)
2Θ range for data collection/°	4.11 to 51.996	4.74 to 62.084
Index ranges	$-13 \le h \le 13$, $-15 \le k \le 14$, $-16 \le l \le 16$	$-35 \le h \le 32$, $-32 \le k \le 28$, $-34 \le l \le 35$
Reflections collected	13234	42716
Independent reflections	5892 [R_{int} = 0.0575, R_{sigma} = 0.0751]	17622 [R_{int} = 0.0619, R_{sigma} = 0.0976]
Data/restraints/parameters	5892/0/355	17622/0/827

Goodness-of-fit on F^2	1.042	1.118
Final R indexes [I>=2σ (I)]	R_1 = 0.0770, wR_2 = 0.2078	R_1 = 0.1254, wR_2 = 0.3549
Final R indexes [all data]	R_1 = 0.1335, wR_2 = 0.2320	R_1 = 0.2882, wR_2 = 0.4403
Largest diff. peak/hole / e $Å^{-3}$	0.56/-0.25	0.39/-0.27
CCDC Number	2191813	2191812

S6.1. Calculation of the Interplanar Angles

The interplanar angles were calculated as the average of the angles between the plains of each furan ring and its neighbors (as demonstrated in Supplementary Figure 73). This method was chosen due to the curvature of the molecule, which rendered the calculation of dihedral angles inaccurate. Calculated this way, the interplanar angles are 26.4±4.9 and 12.4±4.4 for **C6** and **C7** respectively.

a) b)

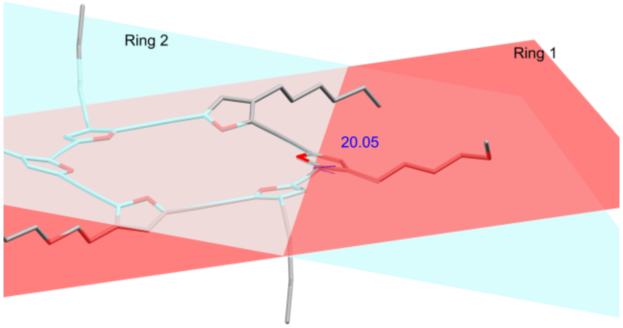

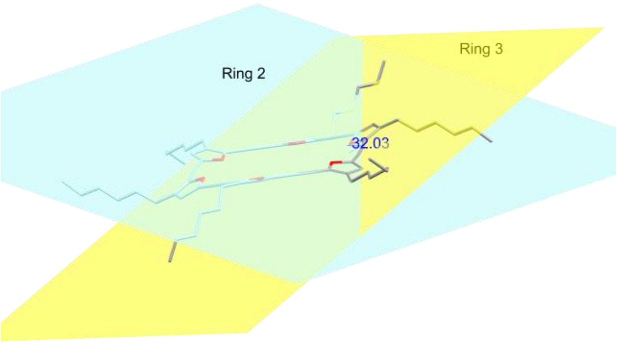

Supplementary Figure 72. Interplanar angles between (a) rings 1 and 2 and (b) rings 2 and 3, taken from the crystal structure of **C6**.

References

(1) Qiu, Y.; Fortney, A.; Tsai, C.-H.; Baker, M. A.; Gil, R. R.; Kowalewski, T.; Noonan, K. J. T. Synthesis of Polyfuran and Thiophene-Furan Alternating Copolymers Using Catalyst-Transfer Polycondensation. *ACS Macro Lett.* **2016**, *5* (3), 332–336. https://doi.org/10.1021/acsmacrolett.5b00666.

(2) Frisch, M. J.; Trucks, G. W.; Schlegel, H. B.; Scuseria, G. E.; Robb, M. A.; Cheeseman, J. R.; Scalmani, G.; Barone, V.; Petersson, G. A.; Nakatsuji, H.; Li, X.; Caricato, M.; Marenich, A. V.; Bloino, J.; Janesko, B. G.; Gomperts, R.; Mennucci, B.; Hratchian, H. P.; Ortiz, J. V.; Izmaylov, A. F.; Sonnenberg, J. L.; Williams; Ding, F.; Lipparini, F.; Egidi, F.; Goings, J.; Peng, B.; Petrone, A.; Henderson, T.; Ranasinghe, D.; Zakrzewski, V. G.; Gao, J.; Rega, N.; Zheng, G.; Liang, W.; Hada, M.; Ehara, M.; Toyota, K.; Fukuda, R.; Hasegawa, J.; Ishida, M.; Nakajima, T.; Honda, Y.; Kitao, O.; Nakai, H.; Vreven, T.; Throssell, K.; Montgomery Jr., J. A.; Peralta, J. E.; Ogliaro, F.; Bearpark, M. J.; Heyd, J. J.; Brothers, E. N.; Kudin, K. N.; Staroverov, V. N.; Keith, T. A.; Kobayashi, R.; Normand, J.; Raghavachari, K.; Rendell, A. P.;

Burant, J. C.; Iyengar, S. S.; Tomasi, J.; Cossi, M.; Millam, J. M.; Klene, M.; Adamo, C.; Cammi, R.; Ochterski, J. W.; Martin, R. L.; Morokuma, K.; Farkas, O.; Foresman, J. B.; Fox, D. J. Gaussian 16 Rev. C.01, 2016.

(3) Casademont-Reig, I.; Guerrero-Avilés, R.; Ramos-Cordoba, E.; Torrent-Sucarrat, M.; Matito, E. How Aromatic Are Molecular Nanorings? The Case of a Six-Porphyrin Nanoring**. *Angew. Chem. Int. Ed.* **2021**, *60* (45), 24080–24088. https://doi.org/10.1002/anie.202108997.

(4) Geuenich, D.; Hess, K.; Köhler, F.; Herges, R. Anisotropy of the Induced Current Density (ACID), a General Method To Quantify and Visualize Electronic Delocalization. *Chem. Rev.* **2005**, *105* (10), 3758–3772. https://doi.org/10.1021/cr0300901.

(5) Plasser, F.; Glöcklhofer, F. Visualisation of Chemical Shielding Tensors (VIST) to Elucidate Aromaticity and Antiaromaticity**. *Eur. J. Org. Chem.* **2021**, *2021* (17), 2529–2539. https://doi.org/10.1002/ejoc.202100352.
