# Supplementary material for: Alternating behavior in furan-acetylene macrocycles reveals the size-dependency of Hückel’s rule in neutral molecules
Source: Commun Chem. 2023 May 27;6:100. doi: 10.1038/s42004-023-00902-9 (PMC10224948; doi:10.1038/s42004-023-00902-9)
Supplement: Supplementary file 2 — Description of Additional Supplementary Files [file 42004_2023_902_MOESM2_ESM.pdf]

Description of Additional Supplementary Files

File name: Supplementary Data 1

Description: Cartesian coordinates of calculated structures.

File name: Supplementary Data 2

Description: Crystallographic information file for C6.

File name: Supplementary Data 3

Description: Crystallographic information file for C7.
